# Revision of the genus *Menevia* Schaus, 1928 (Lepidoptera, Mimallonoidea, Mimallonidae) with the description of 11 new species

**DOI:** 10.3897/zookeys.566.6982

**Published:** 2016-02-18

**Authors:** Ryan A. St. Laurent, Jason J. Dombroskie

**Affiliations:** 1 Cornell University, Comstock Hall, Department of Entomology, Ithaca, NY 14853-2601 USA

**Keywords:** *Mimallo*, Neotropical, neotype, *Pamea*

## Abstract

The Neotropical genus *Menevia* Schaus, 1928 is revised to include 18 species, 11 of which are new. Two species, *Menevia ostia* **comb. n.** and *Menevia parostia* **comb. n.** are transferred from *Pamea* Walker, 1855 to *Menevia*. Four species-groups are diagnosed for the first time based on external characters and male genitalia morphology. The following new species are described: *Menevia rosea* **sp. n.**, *Menevia torvamessoria* **sp. n.**, *Menevia magna* **sp. n.**, *Menevia menapia* **sp. n.**, *Menevia mielkei* **sp. n.**, *Menevia australis* **sp. n.**, *Menevia vulgaris* **sp. n.**, *Menevia franclemonti* **sp. n.**, *Menevia vulgaricula* **sp. n.**, *Menevia cordillera* **sp. n.**, and *Menevia delphinus* **sp. n.**. A neotype is designated for *Mimallo plagiata* Walker, 1855, which has since been placed in *Menevia*. *Mimallo saturata* Walker, 1855 is interpreted to be a *nomen dubium*.

## Introduction

Until recently, very little revisionary work has been done with the family Mimallonidae. Schaus (1928) was the last to revise the family completely, describing most of the genera that are currently recognized. In addition to organizing the family by erecting numerous genera, Schaus (1928) also separated Mimallonidae into two subfamilies. This subfamily arrangement, however, has been deemed by most contemporary authors (Pearson 1951, 1984, Franclemont 1973, Herbin 2012, St Laurent and Dombroskie 2015, but see Becker 1996) not to reflect their phylogeny. We continue to recognize the lack of a clear subfamily arrangement awaiting a higher-level treatment of the family.

Adding to the poor understanding of the higher-level arrangement of the family are a few weakly diagnosed genera currently persisting as catch-alls to subsume numerous recently described species (Herbin 2012, Herbin and Mielke 2014, Herbin 2015). The present taxonomic treatment examines one of the more morphologically distinct genera, *Menevia* Schaus, 1928, and provides autapomorphies for the genus so that new species described herein can be accurately placed.

The genus *Menevia* currently consists of five Central and South American species: *Menevia plagiata* (Walker, 1855), *Menevia lantona* (Schaus, 1905), *Menevia lucara* (Schaus, 1905), *Menevia alurca* Herbin & Mielke, 2014, and *Menevia pallida* Herbin & Mielke, 2014. No synonyms or other names have been formerly assigned to *Menevia*. We diagnose the genus *Menevia* based on external characters, re-describe all five currently known species, describe each previously undescribed female, describe 11 additional new species, and move two species currently assigned to the genus *Pamea* Walker, 1855 into *Menevia*.

## Methods

Dissections were performed as described by Lafontaine (1987) except when same-day analysis was required, whereby abdomens were heated in a 10% KOH solution for 20–30 minutes. Not all genitalia were prepared on slides to allow for three-dimensional analysis of the complex male genitalia. Genitalia and abdomens, when not slide mounted, are preserved in glycerol filled microvials. Morphological, including genitalia, terminology follows Lemaire and Minet (1999).

Costa Rican material of *Menevia ostia* was obtained from the biodiversity inventory of the Área de Conservación Guanacaste (ACG) (Janzen et al. 2009, Janzen and Hallwachs 2011). Some specific localities within the ACG that do not appear in gazetteers are local names, but the provided GPS coordinates give the true location. Full information content for all ACG specimens can be found at http://janzen.sas.upenn.edu by searching the voucher code (Janzen and Hallwachs 2009).

The holotypes and one neotype were all dissected or, when present, previously made genitalia preparations were examined.

**Specimens from the following collections were examined:**

AMNH
American Museum of Natural History, New York, New York, USA 


CGCM
 Collection of Carlos G. C. Mielke, Curitiba, Paraná, Brazil 


CMNH
Carnegie Museum of Natural History, Pittsburgh, Pennsylvania, USA 


CNC
 Canadian National Collection of Insects, Arachnids and Nematodes, Ottawa, Ontario, Canada 


CPAC
 Coleção Embrapa Cerrados, Planaltina, Distrito Federal, Brazil 


CUIC
Cornell University Insect Collection, Ithaca, New York, USA 


DZUP
 Collection of Pe. Jesus S. Moure, Departamento de Zoologia, Universidade Federal do Paraná, Curitiba, Paraná, Brazil 


FSCA
 Florida State Collection of Arthropods, Gainesville, Florida, USA 


MEM
 Mississippi Entomological Museum, Mississippi State, Mississippi, USA 


MGCL
 McGuire Center for Lepidoptera & Biodiversity, Gainesville, Florida, USA 


MNHN
Muséum nationale d’Histoire naturelle de Paris, France 


MNHU
Museum für Naturkunde der Humboldt-Universität zu Berlin, Germany 


NHMUK
Natural History Museum [formerly British Museum (Natural History)], London, U.K. 


OM
 Collection of Olaf Hermann Hendrik Mielke, Curitiba, Paraná, Brazil 


RAS
 Research collection of Ryan A. St. Laurent, Ithaca, New York, USA 


USNM
 National Museum of Natural History [formerly United States National Museum], Washington D.C., USA 


The symbol ‡ will be used to represent unavailable names in the text (Fletcher and Nye 1982).

Figures were manipulated with Adobe Photoshop CS4 (Adobe 2008). Male genitalia are figured in natural color with CS4 “auto color” used to improve white backgrounds. Female genitalia were treated with “auto tone” in CS4 to darken characters; insets, however, are manipulated only with “auto color.” Most adult specimens were photographed in natural light with an Apple iPhone 5S, additional adult photos were provided by CGCM and NHMUK. Genitalia were photographed with a Macroscopic Solutions Macropod Pro and Canon EOS 6D DSLR camera body using the Macro Photo MP-E 65mm f/2.8 1–5× Manual Focus Lens for EOS. Thirty (3×) photographs were taken of each specimen in ethanol under glass, and stacked using Zerene Stacking Software. Maps were created with SimpleMappr (Shorthouse 2010) and edited with CS4. All geographical coordinates are approximate, and are based on the localities provided on specimen labels. GPS data were acquired with Google Earth.

## Results and discussion

### Menevia

Taxon classificationAnimaliaLepidopteraMimallonidae

Schaus, 1928

#### Type species.

*Cicinnus lantona* Schaus, 1905; Schaus 1928: 665, by original designation.

#### Diagnosis.

**Figure 1.**
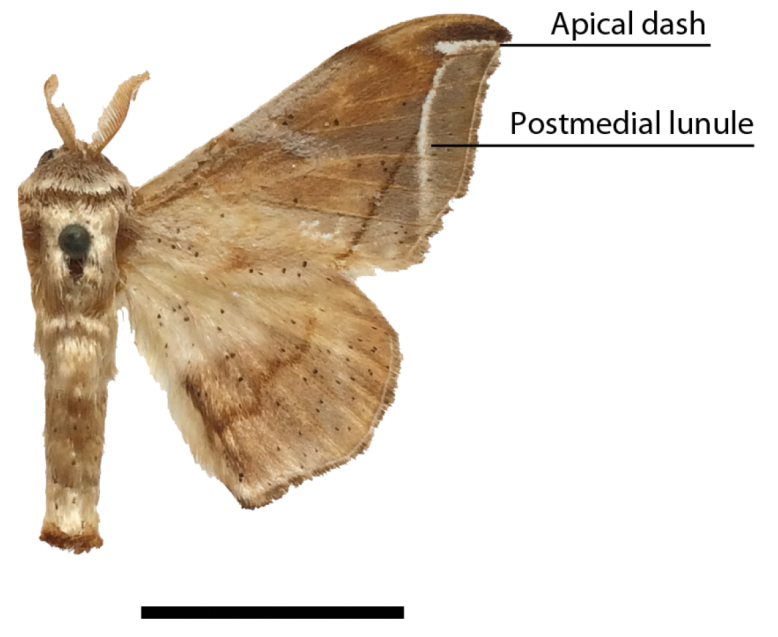
Important forewing characters. *Menevia lantona*, male, Suriname, Moengo, Boven Cottica River (CUIC). Scale bar = 1 cm.

**Figure 2.**
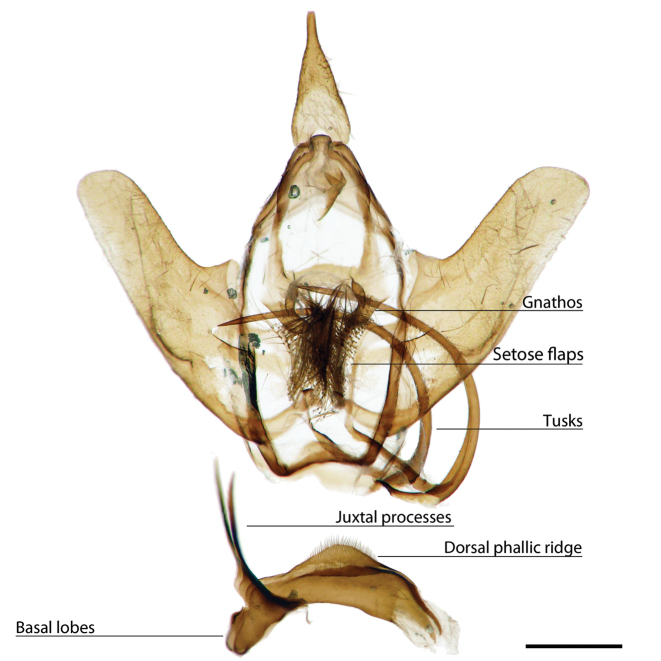
Important male genitalia characters. *Menevia mielkei*, holotype male, Brazil, Minas Gerais, Estação Biológica de Caratinga, Caratinga [CGCM diss.: OM 61.563] (DZUP). Photo courtesy of CGCM. Scale bar = 1 mm.

*Menevia* can be recognized by the contrast between the usually gray submarginal area and the darker gray, brown, yellowish, or rarely pink medial area of the forewing and the presence of a white apical dash. This apical dash becomes the “postmedial lunule” (see Fig. 1), which in most species-groups consists of a variably distinct white line originating from the apical dash. The white mark follows the postmedial line from near the apex to one quarter or to one half the length of the postmedial line until sweeping outward toward the wing margin, either at an acute angle or nearly perpendicular to the postmedial line. In the *plagiata* species-group only does the postmedial lunule not sweep outward from the postmedial line, but follows it as a white outlining band that may be interrupted. In other genera where markings similar to the postmedial lunule exist, they are not white and contrasting against a gray submarginal area. Finally, the male genitalia are also unique among Mimallonidae. They are complex structures (see Fig. 2) with a pair of distinct, elongated tusks pointing outwards, originating from the modified transtilla that itself extends inward into the body from attachments on either side of the inner costal apopdemes of the valves. The elongated tusks pass outward between a pair of weakly sclerotized setose flaps. The juxta is fused to the phallus, encircling it, and is without clear form except for a pair of juxtal processes, which curve toward the distal end of the phallus and are superior to it. Phallic shape is diverse, but always recognizable by the presence of the attached juxtal processes dorsally. The phallus is longitudinally rolled, roughly forming a “U” in cross section, and is open lengthwise along the dorsum where the edges of the rolled phallic structure do not meet. The dorsal, left edge of the phallus is usually uneven, with extensive ridges or protuberances of varying size and shape.

#### Description.

**Male.** *Head*: Small, scales on frons swept ventrad, either the same color as vertex or darker ventrally, eyes large comprising about half to two-thirds of head area, eyes usually bordered posteriorly by darker collar of scales reaching labial palpi, labial palpi small, segments variably defined ventrally depending on thickness of vestiture, incrementally smaller in length distally, dorsally and laterally with darker scales contrasting with overall lighter coloration of head. Antenna bipectinate to tip, scape and pedicel tufted. Ocelli and chaetosemata absent. *Thorax*: Tan, gray, or straw colored. Densely covered in scales of varying widths with interspersed darker petiolate scales, scales of prothoracic collar finer, lighter, overlapping scales of mesothorax. *Legs*: Vestiture thick, scales long, especially on femur and tibia, coloration as for thorax, petiolate scales present. Tibial spurs often scaled, about one fourth length of tibia, apex may be somewhat hooked. *Forewing dorsum*: Forewing length: 14–28 mm. Triangular, outer margins concave on apical half, apex usually falcate. Ground color yellow, brown, gray, or gray-brown, lightly or moderately speckled by dark petiolate scales. Discal spot absent or faintly marked by light gray, no hyaline patches present. Dark postmedial line always present, either straight or with slight undulations. Gray submarginal area usually contrasting with medial area, submarginal area with a variably distinct white line originating from apical dash, white mark follows postmedial line from apex to one quarter to one half the length of postmedial line until sweeping outward toward wing margin, either at an acute angle or nearly perpendicular to postmedial line. In some species, white line wider, forming a complete or interrupted band following postmedial line, not sweeping toward wing margin. Antemedial line, if present, faint and undulated. *Forewing venter*: As in forewing dorsum but postmedial line may be fainter, antemedial line absent, discal spots may be much darker. *Hindwing dorsum*: Rounded or subtriangular, anal angle often accentuated, similar coloration and patterning as forewings, vague postmedial lunules present but undulated or sharply zigzagged, never dramatically swept to wing margin, antemedial line absent. *Hindwing venter*: Following similar pattern as forewing venter, usually lighter, frenulum with single bristle. *Wing venation*: As for *Cicinnus melsheimeri* (Harris, 1841) in Franclemont (1973) but M_2_ originating closer to M_3_ near posterior, distal corner of cell. *Abdomen*: Short, subtriangular, reaching just barely beyond anal margin of hindwing, depth equal to that of thorax, truncated to slightly upturned posterior tip, coloration a continuation of thoracic color, which varies from yellowish to brown, generally matching the ground color of wings. Longitudinal midventral stripe present or absent. *Genitalia*: Complex; tegumen variable in shape, subtriangular or broader and more rectangular or ovoid, often constricted near base of gnathos. Vinculum usually quadrate ventrally, variable in thickness. Transtilla as rectangular frame extending backward from attachments on either side of the inner costal apopdemes of valves, elongated tusks extend outward originating from complex transtilla, passing between pair of setae covered flaps. Valves simple, narrow or broad, triangular or ovoid, with or without projections from saccular edge and with or without similar mesal costal projections, projections of left valve usually larger than those of right valve. Setae covered uncus teardrop or bottle-shaped, or acutely triangular, extended apically to sharp, rounded, or quadrate tip. Gnathos as two prominent outward facing or upturned extensions, variable in shape, thickness. Gnathos extensions thick and boxing glove shaped, flattened and triangular, subtriangular, cupped, or ovoid. Anal tube lightly sclerotized, with apex nearly reaching base of uncus. Juxta fused to phallus, enveloping proximal quarter of phallus, pair of juxtal processes curve toward the distal end of phallus and are superior to it. Juxtal processes variable in length, from roughly three quarters the length of the phallus to slightly longer. Juxtal processes vary in sclerotization; processes flattened, rounded or sharp distally. Processes always enveloped in membrane, usually covered in fine setae. Base of phallus with paired, elongated, short, or peg-like diverging lobes. Phallus usually complicated, formed by singular structure rolled into a cylindrical shape, dorsal edges of phallus do not meet, gap exists between edges, exposing hollow cavity within containing the vesica. Left edge of rolled phallus variously shaped, either flat and simple or with ridge or protuberance dorsally. Distal tip of phallus separated into two distinct points. Vesica very weak, bag-like or somewhat elongate and tapered apically, cornuti absent. **Female.** Generally similar to male, degree of sexual dimorphism variable. *Head*: As in male, antennae sometimes smaller overall. *Thorax*: As in male. *Legs*. As in in males except tibial spurs sometimes more heavily scaled. *Forewing dorsum*: Forewing length: 15–39 mm. Elongated or subtriangular, outer margins concave near apex, convex near tornus, apex usually falcate. Coloration and markings as in corresponding males of each species. *Forewing venter*: As in forewing dorsum but postmedial line may be fainter, antemedial line absent, discal spots may be much darker than in males. *Hindwing dorsum*: As in males but always more rounded, broader. *Hindwing venter*: Following similar pattern as forewing venter, frenulum present with multiple bristles or highly reduced. *Abdomen*: As in male but stouter, presence of midventral stripe corresponds to presence/absence in conspecific males, sternites of VIII as pair of elongated sclerotized bands of varying width and length. *Genitalia*: Very simple; VIII prominently sclerotized laterally, sometimes with appendicular apophyses dorsolaterally in addition to apophyses anteriores. Tergite of VIII upturned mesally, posteriorly pointed, smoothly curving, or replaced by bulbous sac-like sclerotized structure. Apophyses anteriores slightly shorter or same length as apophyses posteriores. Lamella antevaginalis of varying width and shape, usually forming a semicircle or “V”. Ostium bursae unsclerotized. Ductus bursae usually short. Corpus bursae bag-like, without any sclerotized structures, rarely preserved, appendix bursae elongated. Papillae anales quadrate or slightly elongated, subtriangular, covered in fine setae.

#### Remarks.

The genus *Menevia* consists of four clear species-groups, which are delineated here for the first time. The species-groups are readily differentiated by the adult morphology; namely the degree of sexual dimorphism, size, forewing maculation and shape, ground color, and male genitalia characteristics.

#### Key to species-groups of *Menevia*

**Table:** 

1	Forewing submarginal area with white, curved, lunule originating from apical dash (Fig. 1)	**2**
–	Forewing postmedial lunule replaced by continuous or interrupted white band along exterior edge of postmedial line	***plagiata* species-group**
2	Ground color light tan, yellow, or orange, contrasting with gray submarginal area, postmedial lunule usually very weakly curved toward wing margin, forewings weakly falcate	**3**
–	Ground color mixture of deep red-brown and dark gray, not strongly contrasting with gray submarginal area except for when medial area more red-brown than gray, postmedial lunule sharply curved toward wing margin, forewings falcate	***lucara* species-group**
3	Sexual dimorphism pronounced. Gray submarginal area highly contrasting with yellowish or golden medial area, submarginal area always with small accessory white mark near tornus, hindwing postmedial line mostly straight. Female: forewings broad, nearly ovoid; male: phallus always with dorsal ridge or variously shaped protuberance	***ostia* species-group**
–	Male and female hardly differentiable. Gray submarginal area moderately or sometimes only weakly contrasting with light tan, yellowish, orange, or rarely pinkish medial area, small white accessory mark near tornus absent in most species, hindwing postmedial line usually wavy, especially near anal margin. Male: phallus irregularly shaped, usually without dorsal ridge, but when present, anteriorly situated, triangular	***lantona* species-group**

### *lantona* species-group

The *lantona* species-group, containing the type species *Menevia lantona* by original designation, is the only species-group with very weak sexual dimorphism. This group includes the following species: *Menevia lantona*, *Menevia rosea* sp. n., *Menevia torvamessoria* sp. n., and *Menevia magna* sp. n. The females of two species belonging to this group were available for study, and both are exceptionally reminiscent of the males, with only slightly more elongated forewings and meagerly larger size. Species belonging to the *lantona* species-group are generally small for the genus, containing some of the smallest species of *Menevia*. The shape of the forewings of both males and females are not overly elongated, barely falcate, with weakly curving to almost straight postmedial lunules, and with a light ground color, which is usually yellowish with light shades of brown and tan. One species however, is mostly pink. The flat, triangular, or cupped processes of the gnathos and the usually broad phallus characterize male genitalia of this species-group.

#### Key to *lantona* species-group

**Table:** 

1	Ground color orange, yellow-tan, or dark tan, never with any pink suffusion, dorsum of phallus variable	**2**
–	Ground color suffused with pink, phallus with well-defined dorsal protuberance	***Menevia rosea* sp. n.**
2	Forewing postmedial line straight or convex, faint or very thick and contrasting, abdomen never with midventral stripe. Panama and northern South America	**3**
–	Forewing postmedial line with slight inward kink on inferior half, phallus with triangular dorsal ridge, venter of abdomen with quarter-length or complete midventral stripe. Southeastern Brazil	***Menevia magna* sp. n.**
3	Ground color orange-yellow, forewing postmedial line very dark and contrasting, genitalia with tegumen nearly circular, uncus hooked, phallus pistol shaped and bent mesally, tubular and elongated distally	***Menevia torvamessoria* sp. n.**
–	Ground color light tan, fading to yellow in older specimens, forewing postmedial line usually thin and not highly contrasting against surrounding color, tegumen rectangular or somewhat ovoid, especially when prominently constricted at base of gnathos, uncus barely hooked, phallus irregularly shaped but not bent mesally or tubular distally	***Menevia lantona***

#### Menevia lantona

Taxon classificationAnimaliaLepidopteraMimallonidae

(Schaus, 1905)

Figs 1 , 6–10 , 72 , 92 ; Map 1

Cicinnus lantona Schaus, 1905: 327–328
Cicinnus lantona ; Dyar 1914
Cicinnus lantona ; Lucas 1915
Menevia lantona ; Schaus 1928: fig. ♂ 88f
Menevia lantona ; Forbes 1942: Plate 14, fig. 102
Menevia lantona ; Becker 1996
Menevia lantona ; Herbin and Mielke 2014: figs ♂ 57, 58

##### Type material.

**Holotype**, ♂: **FRENCH GUIANA**: St. Jean, Maroni, F. Guiana/ Collection Wm. Schaus/ *Perophora lantona* type Schaus/ Type No.: 8894 U.S.N.M./ USNM-Mimal: 1122/ St. Laurent diss.: 3-7-15:1/ (USNM) [examined]. No paratypes. Type locality: French Guiana: St. Jean du Maroni.

##### Additional specimens examined.

(65 ♂, 1 ♀ total) **BRAZIL: Amazonas**: 1 ♂, Reserva Ducke, km. 26 Manaus-Itacoatiara Highway: 19.V.1972, E.G., I. & E.A. Munroe, St. Laurent diss.: 3-9-15:1 (CNC). 1 ♂, Codajás: IV.1907, S.M. Klages, Rothschild Bequest, BM 1939-1 (NHMUK). 2 ♂, Fonte Boa: V.1906, IX.1906, S.M. Klages, Rothschild Bequest, BM 1939-1 (NHMUK). **COLOMBIA**: 2 ♂, Muzo, 400–800 m: Coll. Fassl, ex. Joicey Coll. Brit. Mus, 1925–157, BMNH(E) 1378744, St. Laurent diss.: 6-29-15:7 (NHMUK); Dognin Collection, USNM-Mimal: 2569, St. Laurent diss.: 3-7-15:7 (USNM). 1 ♂, East Cordillera, Muzo: A. & E. Fassl, 1911 (MNHU). **ECUADOR**: 2 ♂, Napo, Rio Napo, Biol. Sta. Jatun Satcha, 380–400 m: 12–16.IV.1990, S.J. Weller, P. Batra, & M.J. Ryan, USNM-Mimal: 2727, St. Laurent diss.: 3-7-15:8, 7-7-15:4 (USNM). 1 ♂, Napo, 33 km. N. Tena, 27 km. E. on Loreto Rd., 3600 ft: 2.XI.1998, black light, leg. J.S. Miller, St. Laurent diss.: 7-7-15:3 (AMNH). **FRENCH GUIANA**: 10 ♂, 1 ♀, St. Jean du Maroni: Collection Wm Schaus, one male with label: “Menevia lanthona Schaus topotype”, female with label: USNM-Mimal: 2574 and St. Laurent diss.: 7-8-15:1 (USNM); 16.I.1978, Porion (MNHN); Received from E. LeMoult, Rothschild Bequest, BM 1939-1 (NHMUK); VII–VIII.1904, E. LeMoult, Rothschild Bequest, BM 1939–1 (NHMUK); “1905-14”, Rothschild Bequest, BM 1939-1 (NHMUK). 1 ♂, Pied Saut, Oyapok River: XII.1917, S.M. Klages, C.M. Acc. 6111, St. Laurent diss.: 3-7-15:2 (CMNH). 2 ♂, St. Laurent du Maroni: IV, Dognin Collection, USNM-Mimal: 2571, St. Laurent diss.: 3-7-15:4 (USNM); II.1906, E. LeMoult, Rothschild Bequest, BM 1939–1 (NHMUK). 1 ♂, Regine Rte de l’Est. km 65, el. 100 m: 11.II.1991, at lights, Coll. C. Snyder (AMNH). 1 ♂, Piste Coralie, PK 2: 11.VI.1993, L. & A. Sénécaux (MNHN). 1 ♂, Route de Regina, PK 62, Piste de Coralie, PK 2.2: 24.VII.1998, H. de Toulgoët & J. Navatte (MNHN). 1 ♂, Route de Regina, PK 62, Auberge des Orpailleurs: 23.VII.1998, H. de Toulgoët & J. Navatte, Mus. nat. Hist. nat. don de H. de Toulgoët (MNHN). 1 ♂, Piste de Kaw, PK 7: 4.VIII.1996, H. de Toulgoët & J. Navatte réc. (MNHN). 1 ♂, Piste de Nancibo: 5.XI.1983, UV, J. Minet & D. Dauthuille (MNHN). 2 ♂, Roura, Route de Kaw, Near Fourgassie, N04°38.643', W52°17.988', 280 m: 26.X.2014, 28.X.2014, P. Sammut leg. (CUIC). 2 ♂, Cayenne, Patawa, N04°32.024', W52°07.684', 275 m: 27.X.2014, P. Sammut leg. (CUIC; RAS). **GUYANA**: 1 ♂, Tumatumari, Rio Potaro, Br. Guiana: Ac. 5615 (AMNH). 1 ♂, Tumatumari: XII.1907, S.M. Klages, Rothschild Bequest, BM 1939–1 (NHMUK). **PANAMA**: 2 ♂, La Cabima: V.1911 [1931?], August Busck, USNM-Mimal: 2567, 2568, St. Laurent diss.: 3-7-15:10 (USNM). 1 ♂, La Chorrera: “May 12”, Aug. Busck, USNM-Mimal: 2566, St. Laurent diss.: 3-7-15:11 (USNM). 21 ♂, Barro Colorado Island, Canal Zone: 16.IV.1941, X.1941, 10.X.1941, 19.X.1941, [other data illegible], at light, J. Zetek collector, Nos. 4884, 4884A,B, USNM-Mimal: 2716–2721 (USNM); 28–30.IV.1964, 1–9.V.1964, 10–17.V.1964, W.D. & S.S. Duckworth, USNM-Mimal: 2709–2715, St. Laurent diss.: 3-7-15:13 (USNM); 22.IV.1979, at light, Silberglied/Aiello, USNM-Mimal: 2724, 2725, 2726, St. Laurent diss.: 3-7-15:14 (USNM); 8.V.1935, A. Friedman (CUIC); 25.X, 31.X, 3.XI, 7–XI, M. Bates coll., St. Laurent diss.: 3-7-15:12 (CUIC). 2 ♂, “Rep. de Panama”: XII.1935-I.1936, L.M. Smith, J.G. Franclemont diss.: 1770 (CUIC). **SURINAME**: 1 ♂, Moengo, Boven Cottica River: 19.V.1927, Cornell Univ. Lot 760, Sub 67, St. Laurent diss.: 3-7-15:3 (CUIC). 2 ♂, Aroewarwa Creek, Maroewym Valley: III.1905, IV.1905, S.M. Klages, Rothschild Bequest, BM 1939–1 (NHMUK). **VENEZUELA**: 1 ♂, Caripito: 14.V.1942, Gift of New York Zoo. Soc. Dept. Tropical Research, William Beebe, Dir., St. Laurent diss.: 3-7-15:6 (AMNH).

##### Diagnosis.

Sexual dimorphism is weak; the females are only slightly larger. Thus both sexes of *Menevia lantona* are recognizable by their small to moderate size, only slightly falcate forewing apices, and yellowish tan to gold ground color with gray highlights, especially near the discal region. The postmedial lunule is bright white and only barely curved outward toward the forewing margin, not sharply curving as in other species-groups. The phallus is broad, usually with a small protuberance dorsally but always without a dorsal ridge. The gnathos processes are unique in that they are very broad, flat, and subtriangular, not oblong or thick as in most other species-groups. Female genitalia are separable from those of the similar female of *Menevia magna* sp. n. by the tergite of VIII, which is thin and upturned mesally rather than being replaced by a lightly sclerotized sac as in *Menevia magna* sp. n.

##### Description.

**Male.** *Head*: Straw or tan colored, eyes bordered posteriorly by dark brown collar of scales reaching labial palpi, labial palpi small, segments weakly defined ventrally due to ventral tufts, dorsally with darker scales contrasting with overall straw coloration. Scape and pedicel weakly tufted. *Thorax*: As for genus but tan or gold, fading to straw. *Legs*: As for genus. Tibial spurs thin apically, terminal third not scaled, especially ventrally. *Forewing dorsum*: Forewing length: 15–18 mm, avg.: 16.2 mm, n = 28. Triangular, apical half of outer margins concave, convex near tornus, apex slightly falcate. Ground color yellowish tan to gold with varying degrees of gray, especially near discal region, overall lightly speckled by dark petiolate scales. Discal spot faintly marked by light gray. Apex marked by black scales above apical dash. Straight or slightly undulating postmedial line black or brown. Antemedial area lighter, submarginal area gray with tan coloration near tornus, postmedial lunule originating from near where apical dash meets postmedial line, lunule follows postmedial line from apex to one third length of postmedial line where lunule smoothly curves outward toward wing margin, forming roughly 45 degree angle with postmedial line. Antemedial line very faint or absent, if present, brown, undulating. *Forewing venter*: As in forewing dorsum but postmedial line fainter, antemedial line absent; usually rounded discal spot present, small, black. *Hindwing dorsum*: Rounded with margin weakly pointed mesally, anal angle weakly accentuated, similar coloration and patterning as forewings, vague postmedial lunule originating near anterior margin undulating, not steeply swept to margin, antemedial line absent, postmedial line undulating, especially near anal angle. *Hindwing venter*: Following similar pattern as forewing venter but discal mark not always present. *Abdomen*: As for genus. Coloration a continuation of tan or golden thoracic color. Midventral stripe absent. *Genitalia*: (Fig. 72) n = 16. Tegumen rectangular or somewhat ovoid, especially when prominently constricted at base of gnathos. Vinculum narrow, somewhat quadrate ventrally. Valves asymmetrical, relatively narrow, saccular edge of left valve with large triangular tooth proximal to transtilla, right valve with tooth much reduced in size, both valves with smaller mesal costal teeth immediately above saccular edge teeth, apex of mesal tooth pointed toward saccular edge. Valves rounded apically. Uncus handbell-shaped, truncated apically, apex rounded. Gnathos as two prominent, moderately flattened, subtriangular outward facing flaps, upturned where they converge over phallus. Juxtal processes shorter than phallus, flattened, slightly curved, covered in short setae. Base of phallus with paired, somewhat elongated, rounded, diverging, backwards facing fingerlike lobes. Phallus broad, widened mesally, usually with a small protuberance dorsally but extended dorsal ridge absent. Left edge of rolled phallus simple, without ridge-like process, distal tip of phallus separated into two distinct, bent points. Vesica small, bag-like. **Female.** *Head*: As in male, antennae slightly smaller overall. *Thorax*: As in male. *Legs*: As in male. *Forewing dorsum*: Forewing length: 21 mm, n = 1. As in male but barely more elongated, only slightly broader, less falcate, postmedial line bent mesally. Faint brown antemedial line present, undulated. *Forewing venter*: As in forewing dorsum but postmedial line fainter, antemedial absent, small black discal spot present. *Hindwing dorsum*: As in male but slightly broader. *Hindwing venter*: Following similar pattern as forewing venter except lighter, frenulum reduced. *Abdomen*: As in male but stouter. Sternites of VIII as pair of elongated sclerotized bands widening toward anterior margin of VIII. *Genitalia*: (Fig. 92) n = 1. Tergite of VIII very thin, converging mesally to form anteriorly directed point. Apophyses anteriores slightly shorter than apophyses posteriores. Lamella antevaginalis ribbon-like, weakly concave mesally. Ductus bursae thin. Papillae anales rectangular when viewed ventrally, covered in setae.

##### Distribution

**Map 1.**
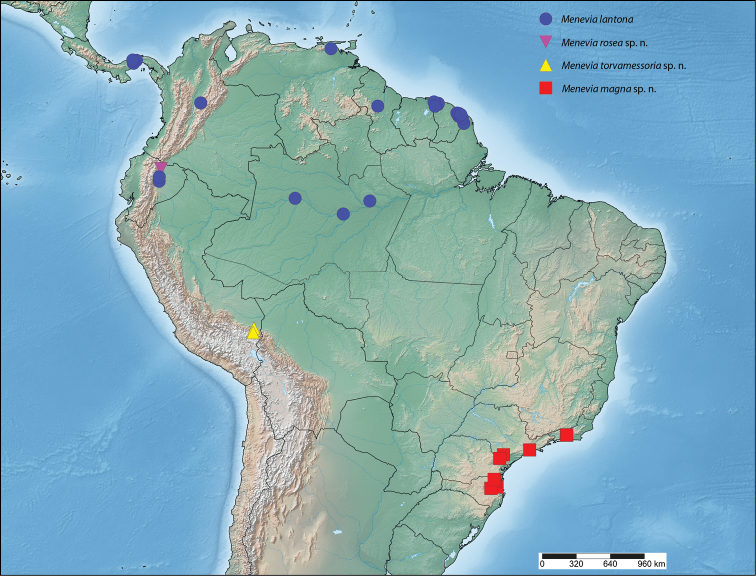
*Menevia lantona* species-group.

(Map 1). *Menevia lantona* is found throughout the Amazonian rainforest in the Guianas, Suriname, northern Venezuela, the Brazilian state of Amazonas, as well as in central Colombia and northcentral Ecuador. This species also ranges into central Panama.

##### Remarks.

As a wide-ranging species, *Menevia lantona* expresses some geographic variation. Most notably, specimens from Colombia are larger (Fig. 9) with somewhat broader forewings, but genitalia show no remarkable differences. Specimens from Panama (Fig. 8) are slightly grayer and a bit darker overall, but like the Colombian populations, show no genitalia differences specifically applicable to this geographic form. Additionally, in specimens from Ecuador, the dorsal point of the phallus is somewhat more pronounced than in other populations, and one specimen from a relatively high elevation (1097 m) in Ecuador has much darker postmedial lines than most other individuals of *Menevia lantona*, somewhat reminiscent of *Menevia torvamessoria* sp. n., to be described below. The genitalia of this unique higher elevation individual however, are completely in line with those of *Menevia lantona*, and cannot be mistaken for the highly remarkable and distinct genitalia of *Menevia torvamessoria* sp. n.

**Figure 3.**
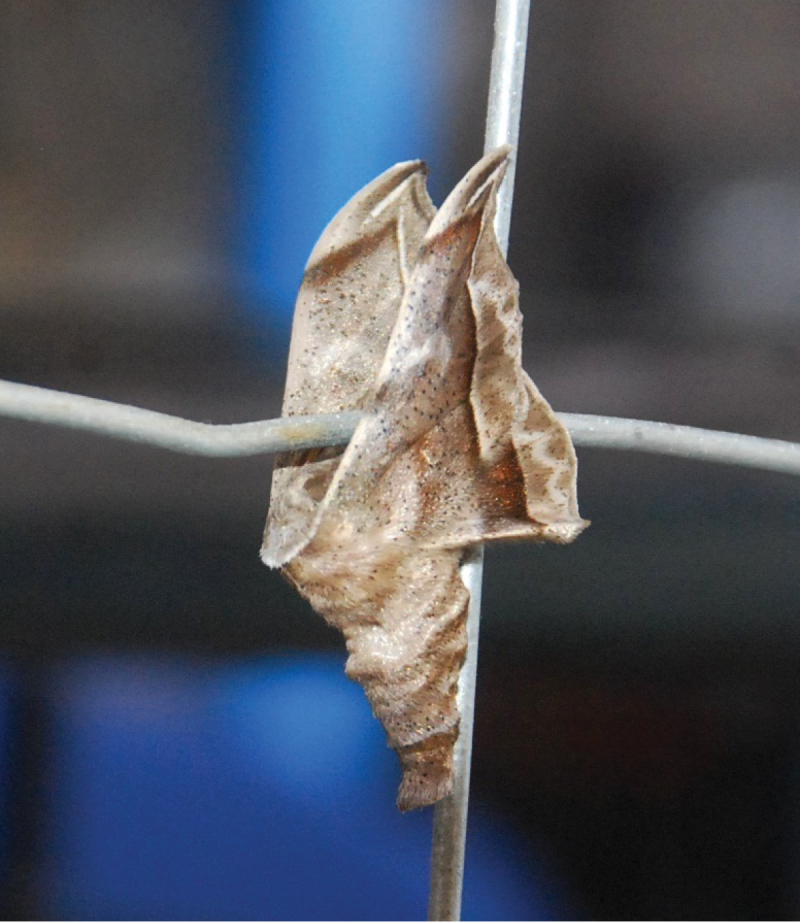
*Menevia* in resting position. *Menevia australis* sp. n., Brazil, Santa Catarina, Laurentino. Photo credit: Miguel Angelo Biz, used with permission.

**Figure 4.**
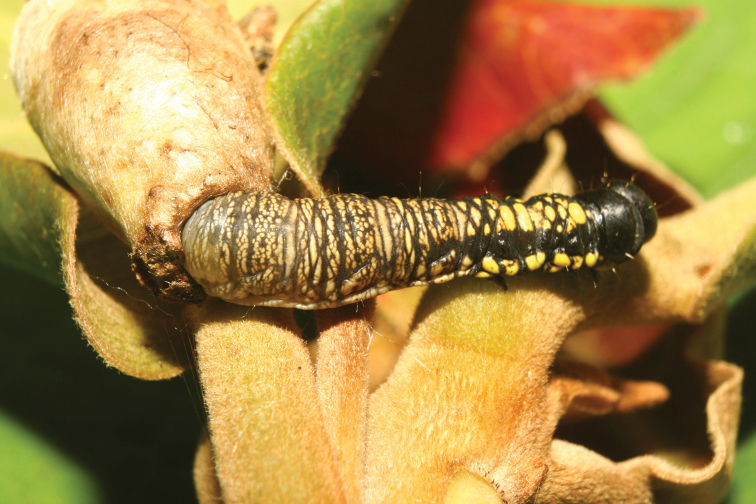
*Menevia ostia* larva with case, lateral view. On unknown host plant, larval length = 33 mm, 13-SRNP-71165. Image courtesy of D. H. Janzen & W. Hallwachs, used with permission.

**Figure 5.**
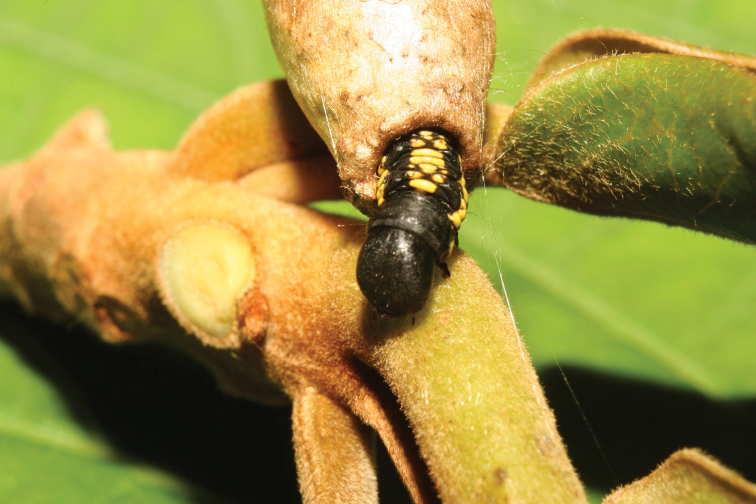
*Menevia ostia* larva with case, anterior view. As for Fig. 4.

**Figures 6–17.**
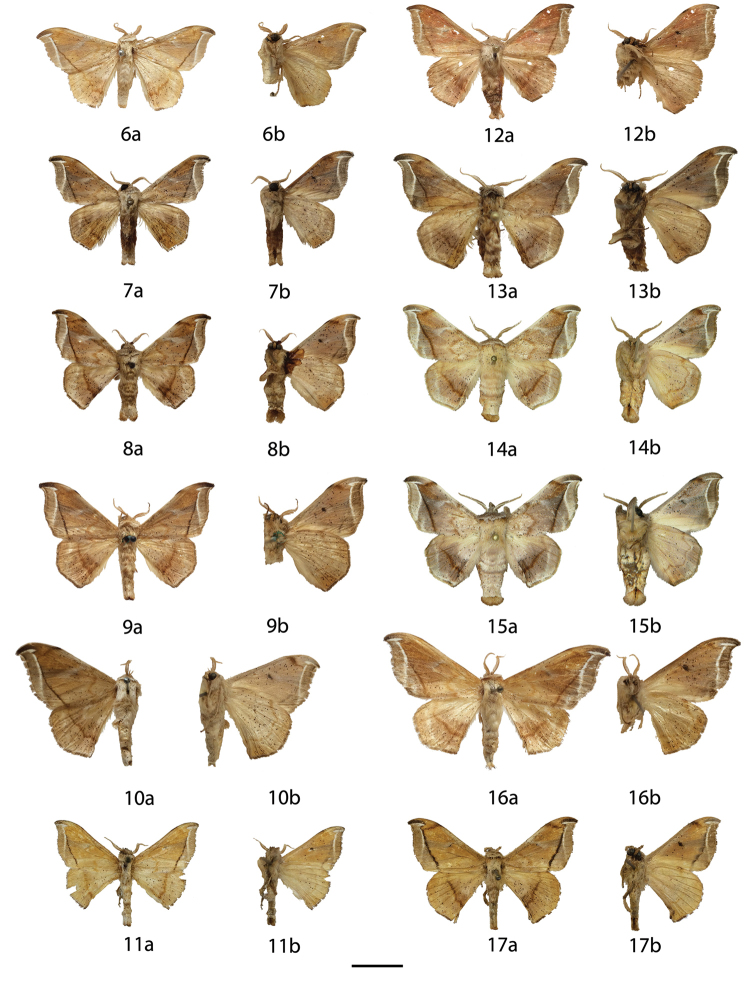
*Menevia lantona* species-group adults, **a** recto, **b** verso. 6 *Menevia lantona* holotype ♂, French Guiana, St. Jean du Maroni (USNM) **7** *Menevia lantona* ♂, French Guiana, Cayenne, Patawa, 275 m (RAS) **8** *Menevia lantona* ♂, Panama (CUIC) **9** *Menevia lantona* ♂, Colombia, Muzo, 800 m (USNM) **10** *Menevia lantona* ♀, French Guiana, St. Jean du Maroni (USNM) **11** *Menevia torvamessoria* holotype ♂, Peru, Puno, La Unión, 2000 ft (NHMUK) **12** *Menevia rosea* holotype ♂, Ecuador, Napo, Simón Bolívar, 1200 m (CMNH) **13** *Menevia magna* holotype ♂, Brazil, Santa Catarina, São Bento do Sul, Rio Natal, 450 m (DZUP) **14** *Menevia magna* paratype ♂, Brazil, São Paulo, Guapiara, Paivinha, 800 m [photo courtesy CGCM] (CGCM) **15** *Menevia magna* paratype ♂, Brazil, São Paulo, Apiaí, 750 m [photo courtesy CGCM] (CGCM) **16** *Menevia magna* paratype ♀, Brazil, Santa Catarina, Dalbérgia (CUIC) **17** *Menevia torvamessoria* paratype ♂, Peru, Puno, S. Domingo, 6500 ft (NHMUK). Scale bar = 1 cm.

#### Menevia rosea sp. n.

Taxon classificationAnimaliaLepidopteraMimallonidae

http://zoobank.org/5E2B3DC6-6B92-48EF-958D-6F67D144563F

Figs 12 , 73 ; Map 1

##### Type material.

**Holotype**, ♂: **ECUADOR**: Ecuador: Napo, Simon Bolivar [Simón Bolívar], Coca River canyon, 1200 m, 16 Aug 1996, Jan Hillman, undisturbed wet forest/ St. Laurent diss.: 3-7-15:9/ HOLOTYPE male *Menevia rosea* St Laurent and Dombroskie, 2016 [handwritten red label]/ (CMNH). No paratypes. Type locality: Ecuador: Napo: Simón Bolívar.

##### Diagnosis.

*Menevia rosea* is distinguishable from all other species in the *lantona* species-group by the pink coloration of the forewings, especially medially and proximal to the apical region. The postmedial lunule is also more weakly curved than in the most similar species, *Menevia lantona*. Genitalia characters should also readily distinguish *Menevia rosea* from other species in the *lantona* species-group. The particularly short phallus has a small, but obvious dorsal ridge, which is lacking in *Menevia lantona*. The phallic ridge of *Menevia rosea* should not be confused with the dorsal protuberance on the phallus of some *Menevia lantona*, as this protuberance is closer to mid-length of the phallus while the phallic ridge of *Menevia rosea* is more distal. The juxtal processes are more curved toward the distal end of the phallus. The valves are more triangular and are particularly broad proximal to the vinculum, the saccular edges of the valves are also particularly straight.

##### Description.

**Male.** *Head*: Brownish gray with pinkish hue, eyes bordered posteriorly by dark brown collar of scales reaching labial palpi, labial palpi small, segments weakly defined ventrally due to ventral tufts, dorsally with darker scales contrasting with overall pinkish gray coloration. Scape and pedicel weakly tufted. *Thorax*: As for genus but light tan, scales of prothoracic collar pinker, tipped with white. *Legs*: As for genus. Tibial spurs very thin, relatively long, terminal third not scaled, especially ventrally. *Forewing dorsum*: Forewing length: 16 mm, n = 1. Triangular, apical half of outer margins concave, apex slightly falcate. Ground color light tan with excessive pink scaling, especially medially and nearing apex before postmedial line, very sparsely speckled by dark petiolate scales. Discal spot faintly marked by light gray. Apex marked by black scales above small apical dash. Dark brown postmedial line mostly straight, somewhat undulating. Antemedial area lighter, less pink, submarginal area faint gray to more tan near tornus, postmedial lunule originating perpendicular to where apical dash meets postmedial line, lunule follows postmedial line from apex to nearly half length of postmedial line where lunule barely curves outward toward wing margin, forming very acute angle with postmedial line. Antemedial line absent. *Forewing venter*: As in forewing dorsum, pink coloration widespread but postmedial line fainter, more curved, antemedial line absent, small black discal spot present. *Hindwing dorsum*: Rounded with margin weakly pointed mesally, anal angle very weakly accentuated, similar coloration and patterning as forewings but with more petiolate scales, vague postmedial lunule originating near anterior margin undulating, not steeply swept to margin, antemedial line absent, postmedial line mostly straight, brown, surrounded by pink. *Hindwing venter*: Following similar pattern as forewing venter, but lighter. *Abdomen*: As for genus. Coloration a continuation of tan coloration of thorax with pink hue. *Genitalia*: (Fig. 73) n = 1. Tegumen rectangular, not constricted at base of gnathos. Vinculum moderately broad, somewhat quadrate ventrally. Valves asymmetrical, triangular, very broad at base, with very straight saccular edge except for large triangular tooth proximal to transtilla on left saccular edge, right valve with tooth much reduced in size, both valves with smaller mesal costal projection immediately above saccular edge teeth, apex of mesal projection pointed outwards. Valves rounded apically. Uncus handbell-shaped, truncated apically, apex rounded. Gnathos as two prominent, flattened, vaguely subtriangular outward facing flaps, upturned where flaps converge over phallus. Juxtal processes shorter than phallus, curved, creased along length, covered in moderately long setae. Base of phallus with paired, somewhat elongated, rounded, diverging, backwards facing fingerlike lobes. Phallus broad, short, widened mesally, with small dorsal ridge anteriorly. Left edge of rolled phallus simple, with small ridge-like process, distal tip of phallus separated into two distinct, straight points. Vesica small, sac-like. **Female.** Unknown.

##### Distribution

(Map 1). *Menevia rosea* is so far known only from the type locality in Ecuador, Napo, Simón Bolívar, at a rather high elevation for the genus, 1200 m.

##### Etymology.

*Menevia rosea* is named for the unique pink coloration of this species, unlike other species of the *lantona* species-group, which are usually tan.

##### Remarks.

This new species is unique externally and is the only species in the *lantona* species-group with pink scales on the wings. Pink scaling is seen in many other *Menevia* species, particularly those in the *lucara* species-group. However, all other characteristics of patterning and the genitalia of *Menevia rosea* perfectly match those characters typical of the *lantona* species-group.

Apart from the interesting external coloration and genitalia characters, the type locality of this species is noteworthy mostly due to the relatively high elevation, 1200 m. The most similar species, *Menevia lantona*, has been collected in the Napo province just 80 km south of the type locality of *Menevia rosea*. Furthermore, the specimen collected 80 km south of the *Menevia rosea* type locality was collected at 1097 m elevation, also high for the genus. We have attributed this particular specimen to *Menevia lantona* due to the complete lack of pink and the genitalia characters. It is worth noting however, that this specimen has much darker postmedial lines on the fore and hindwings than typical *Menevia lantona*. Although the external characters and elevation of the collecting site are somewhat unique, the genitalia characters (St. Laurent diss.: 7-7-15:3) are completely typical of *Menevia lantona*, and are unlike either *Menevia rosea* or the other suspected possibility, *Menevia torvamessoria* sp. n. described below. Other Ecuadorian *Menevia lantona* are from much lower altitudes, 380-400 m. The differences in genitalia and the fact that *Menevia lantona* has been recorded from rather high elevations suggest that *Menevia rosea* is not a mere high elevation form of *Menevia lantona*.

#### Menevia torvamessoria sp. n.

Taxon classificationAnimaliaLepidopteraMimallonidae

http://zoobank.org/68607BD2-04A6-41E5-92F5-C8F709869950

Figs 11 , 17 , 74 ; Map 1

##### Type material.

**Holotype**, ♂: **PERU**: La Union [La Unión], R. Huacamayo, Carabaya [Puno], 2000 ft., wet s., Nov. 1904 (G. Ockenden)/ Rothschild Bequest BM 1939–1/ St. Laurent diss.: 6-29-15:4/ BMNH(E) 1378762/ HOLOTYPE male *Menevia torvamessoria* St Laurent and Dombroskie, 2016 [handwritten red label] (NHMUK). Type locality: Peru: Puno: Carabaya: La Unión.

**Paratypes**, 2 ♂: **BRAZIL: Pará**: 1 ♂, Monte Cristo, Rio Tapajós: Dognin Collection, USNM-Mimal: 2576, St. Laurent diss.: 3-7-15:5 (USNM). **PERU**: 1 ♂, S. Domingo, Carabaya [Puno], 6500 ft.: XII.1902, wet s., “591”, Rothschild Bequest BM 1939–1, St. Laurent diss.: 6-29-15:5, BMNH(E) 1378761 (NHMUK). – All paratypes with the following yellow label: PARATYPE male *Menevia torvamessoria* St Laurent and Dombroskie, 2016.

##### Diagnosis.

Externally, *Menevia torvamessoria* is similar to *Menevia lantona*, but can be distinguished by the darker, yellow-orange ground color (in well-preserved specimens); *Menevia lantona* is lighter, more yellow-tan. Additionally, the postmedial line is very dark and contrasting in *Menevia torvamessoria* and there is a roughly rectangular gray patch of scales that extends from the discal region to the postmedial line. There is a similar gray patch in *Menevia lantona* but is not so well defined. The most outstanding diagnostic features of this new species are in the male genitalia. The phallus is unlike any other in the genus, it is almost pistol shaped and sharply bent halfway along its length. The dorsal surface of the proximal end of the phallus bears a distinct triangular or rounded ridge while the remainder of the length of the phallus is smooth, elongated, and tubular. The juxtal processes are very thin, shorter and more curved in other species in the *lantona* species-group. The gnathos processes are unique in that they are cupped and circular. The valves are unlike the previous species in that they are symmetrical and do not bear teeth on the saccular edge, but instead have distinct sclerotized inward facing lobes at the base of the valves, which conceal the gnathos. The sides of the tegumen are greatly bowed outwards, causing it to appear almost circular. Finally, the scythe-like uncus is acutely triangular and sharply hooked.

##### Description.

**Male.** *Head*: Brownish tan or almost black, eyes bordered posteriorly by dark brown collar of scales reaching labial palpi, labial palpi small, segments moderately well defined ventrally due to ventral tufts, dorsally with darker scales contrasting with overall lighter coloration. Scape and pedicel thinly tufted. *Thorax*: As for genus but light tan, fading to straw. *Legs*: As for genus. Tibial spurs relatively small, only lightly scaled, especially proximally. *Forewing dorsum*: Forewing length: 14–16.5 mm, avg.: 14.8 mm, n = 3. Triangular, apical half of outer margin concave, apex slightly falcate. Ground color orange-yellow with elongate, gray rectangle of scales extending from discal region to postmedial line, overall very sparsely speckled by dark petiolate scales. Discal spot faintly marked by light gray. Apex marked by black scales above scythe-like apical dash. Slightly undulating postmedial line black, strongly contrasting. Antemedial area lighter, submarginal area gray with slight invasion of medial area coloration near tornus, postmedial lunule originating from near where apical dash meets postmedial line, forming scythe-like dash, lunule follows postmedial line from apex to one third length of postmedial line where lunule smoothly curves outward toward wing margin, forming roughly 45 degree angle with postmedial line. Antemedial line very faint or absent, if present, brown, undulating, bowed out near anal margin. *Forewing venter*: As in forewing dorsum but postmedial line fainter, undulations more distinct, antemedial line absent, small black, rounded or oblong discal spot present. *Hindwing dorsum*: Rounded, anal angle weakly accentuated, similar coloration and patterning as forewings, but postmedial lunule almost nonexistent, antemedial line absent, postmedial line more undulated and brown, not black and contrasting, undulations prominent, especially near anal angle. *Hindwing venter*: Following similar pattern as forewing venter, but lighter, discal spot much less distinct or absent. *Abdomen*: As for genus. Coloration a continuation of tan thoracic color. Midventral stripe absent. *Genitalia*: (Fig. 74) n = 3. Tegumen almost circular, sides bowed out dramatically. Vinculum somewhat broad, quadrate ventrally. Transtilla tusks relatively short, thick, bent. Valves symmetrical, base of valves each with bulbous lobe pointed inward, partially covering gnathos. Valves bent mesally. Uncus extremely truncated apically, apex hooked, scythe-like. Gnathos projections as pair of cupped, rounded flaps, situated behind inward facing extensions at base of valves. Juxtal processes shorter than phallus, thin, curved. Base of phallus with paired, short, rounded, diverging, ventrally angled lobes. Phallus pistol shaped, sharply bent mesally, dorsal surface of proximal end of phallus with distinct triangular or rounded ridge, remainder of phallus thin, elongated, and tubular. Left edge of rolled phallus simple, without ridge-like process except for rounded or triangular ridge proximally, distal tip of phallus separated into two distinct points. Vesica small, sac-like. **Female.** Unknown.

##### Distribution

(Map 1). *Menevia torvamessoria* is known from only three locations, one of them being questionable. Two specimens, including the holotype, come from two nearby localities in the Carabaya Province of Peru, in the Cordillera Oriental. The type locality, La Unión, is at about 609 m elevation, while the paratype from nearby Santo Domingo was collected at a rather high elevation for the genus, about 1981 m. From these two data points it seems logical to infer that *Menevia torvamessoria* is a species of moderate elevation, apparently from the Andean Cordillera Oriental. However, a third specimen questionably from Brazil (not shown on Map 1), is discussed in the remarks below.

##### Etymology.

*Menevia torvamessoria* is named for the unique hooked uncus, reminiscent of the Grim Reaper’s (=torva messor Latin) scythe. The name is doubly appropriate to describe the apical dash, which combined with the postmedial lunule of the forewing, is scythe-like, a character seen in all *Menevia* species.

##### Remarks.

Although externally *Menevia torvamessoria* is rather similar to *Menevia lantona*, this new species is wholly unlike any other in the genus when taking into account male genitalia. Every aspect of the genitalia, particularly the hooked uncus, circular tegumen, cupped gnathos processes, bulbous projections at the base of the valves, and the shape of the phallus, are all unique to this species. *Menevia torvamessoria* belongs in the genus *Menevia* due to the presence of the general structure of the genitalia such as the paired gnathos, juxtal processes, and outward facing tusks; but it is difficult to assign this taxon to a species-group based on genitalia alone. External characters, however, such as the size, orange-yellow coloration, and weakly falcate forewings, tentatively allow placement of *Menevia torvamessoria* in the *Menevia lantona* species-group.

In addition to the unusual genitalia, the distribution is also strange, but this may be due to one specimen being incorrectly labeled. One paratype bears a nearly illegible label reading “Monte Cristo, Rio Tapajós, Amazonas” and seems to be from Monte Cristo, in the Brazilian state of Pará, on the Tapajós River. This particular location is very low in elevation, with some hills only as high as about 300 m nearby (as determined from Google Earth), which is quite divergent from the Andean foothill localities of the holotype and paratype. Either the specimen is mislabeled or *Menevia torvamessoria* is very widespread in South America, and apparently very rare. Regardless of the uncertainty of the collecting locality of this paratype, its genitalia display the very unique and bizarre characteristics of the specimens from southern Peru and the ground color of this specimen is the same distinctive orange-yellow of *Menevia torvamessoria*, thus we include this individual in the type series given that there are so few examples of this species available.

#### Menevia magna sp. n.

Taxon classificationAnimaliaLepidopteraMimallonidae

http://zoobank.org/2B8D7774-B967-46AB-A825-32D9D12F0727

Figs 13–16 , 75 , 93 ; Map 1

##### Type material.

**Holotype**, ♂: **BRAZIL: Santa Catarina**: BRAZIL – SC, São Bento do Sul, Rio Natal, 450 m. I. Rank leg, 27.VI.2014, S 26°20'2", W 49°18'30" / St. Laurent diss.: 6-16-15:1/ DZ 32.694/ HOLOTYPE male *Menevia magna* St Laurent and Dombroskie, 2016 [handwritten red label]/ (DZUP). Type locality: Brazil: Santa Catarina: São Bento do Sul.

**Paratypes**, 22 ♂, 3 ♀: **BRAZIL: Santa Catarina**: 1 ♂, Blumenau: X, Pohl, “696”, USNM-Mimal: 2582, St. Laurent diss.: 3-7-15:16 (USNM). 1 ♂, Corupá: VIII.1958, Maller col., No. HRP 889, “*Menevia lanthona*? Schaus Pearson det.”, USNM-Mimal: 2416, St. Laurent diss.: 3-7-15:17 (USNM). 1 ♂, 1 ♀, Nova Bremen [Dalbérgia]: 4.X.1935, 8.VII.1936, Fritz Hoffmann, St. Laurent diss.: 9-7-14:3, 3-7-15:15 (CUIC). 1 ♂, Rio Vermelho [São Bento do Sul], 830 m: VI.1936, A. Maller (NHMUK). 3 ♂, São Bento do Sul, Rio Natal, 550 m: XI.2013, A. Rank leg., Col. C. Mielke 28.001, 28.020, 28.040 (CGCM). 2 ♂, São Bento do Sul, Rio Natal, S 26°20'2", W 49°18'30", 450 m: 27.VI.2014, I. Rank leg, Col. C. Mielke 29.334, 29.422 (CGCM). **São Paulo**: 1 ♂, Alto da Serra: VI.1926, R. Spitz, Rothschild Bequest BM 1939–1, St. Laurent diss.: 6-29-15:6, BMNH(E) 1378759 (NHMUK). 3 ♂, Guapiara, Paivinha, 800 m: 18.VII.2007, C. Mielke leg, Col. C. Mielke 25.826, 26.555, St. Laurent diss.: 6-16-15:2 (CGCM). 3 ♂, Apiaí, 750 m: 18–21.VI.2007, C. Mielke leg, Col. C. Mielke 20.950, 21.350, 21.382 (CGCM). **Rio de Janeiro**: 3 ♂, 1 ♀, Petrópolis: 10.X.1961, 19.II.1963, 25.II.1963, 19.XII.1965, Gagarin leg., ex. col. Gagarin, CGCM diss.: DZ 32.695, DZ 32.695–32.697, 32.722 (DZUP). 2 ♂, 1 ♀, Petrópolis, Independência, 900 m: 19.VI.1934, 16.V.1935, 23.XI.1939, Gagarin leg., ex. col. Gagarin, DZ 32.698–32.700 (DZUP). 1 ♂, Petrópolis, Parque São Vicente: 5.XI.1958, DZ 32.701 (DZUP). – All paratypes with the following yellow label: PARATYPE male/female *Menevia magna* St Laurent and Dombroskie, 2016.

##### Diagnosis.

*Menevia magna* is similar to other species in the *lantona* species-group, except *Menevia rosea*, due to the tan ground color, but can be easily recognized by the larger size on average, the darker gray and brown scaling overall (especially in fresh specimens), and the usually mesally kinked forewing postmedial line. Additionally, the male genitalia are unique in that the valves are very broad and ovoid, the paired processes of the gnathos very thin and fingerlike or acutely triangular, and the smoothly curved phallus with a prominent, sharp, triangular dorsal ridge. Female genitalia are unique in the genus in having a bulbous, sac-like, sclerotized structure dorsally, replacing the usual sclerotization of tergite VIII, which also distinguishes females of *Menevia magna* from the very similar female of *Menevia lantona*.

##### Description.

**Male.** *Head*: Tan to gray, eyes bordered posteriorly by dark brown collar of scales reaching labial palpi, labial palpi small, segments weakly defined ventrally due to ventral tufts, dorsally with very dark scales contrasting with overall tan-gray coloration. Scape and pedicel weakly tufted. *Thorax*: As for genus, but tan, fading to straw. *Legs*: As for genus. Tibial spurs short, mostly scaled. *Forewing dorsum*: Forewing length: 17–19.5 mm, avg.: 17.8 mm, n = 6. Triangular, apical half of outer margin concave, apex falcate. Ground color dark tan-brown fading to lighter yellowish, grayer medially, overall lightly speckled with dark petiolate scales. Discal spot faintly marked by light gray. Apex marked by black scales above apical dash. Dark brown postmedial line straight with slight inward kink mesally. Antemedial area lighter, submarginal area gray with very little tan coloration near tornus, postmedial lunule originating from near where apical dash meets postmedial line, lunule follows postmedial line from apex to one third length of postmedial line where lunule angled outward toward wing margin, forming roughly 45 degree angle with postmedial line. Antemedial line usually present, brown, undulated. *Forewing venter*: As in forewing dorsum but postmedial nearly absent, may be more heavily speckled with petiolate scales, antemedial line absent, small black, rounded or oblong discal spot present. *Hindwing dorsum*: Rounded with margin weakly pointed mesally, anal angle accentuated, similar coloration and patterning as forewings, vague postmedial lunules originating near anterior margin undulating or nearly straight, antemedial line absent, postmedial line undulating, especially near anal angle. *Hindwing venter*: Following similar pattern as forewing venter but lighter, discal spot absent. *Abdomen*: As for genus. Coloration a continuation of tan thoracic color. Midventral stripe weak, either extending from posterior tip to about one quarter length of abdomen or until thorax. *Genitalia*: (Fig. 75) n = 7. Tegumen gumdrop shaped, not constricted at base of gnathos. Vinculum narrow, somewhat quadrate ventrally. Valves very broad, ovoid, asymmetrical, saccular edge of left valve with triangular tooth proximal to transtilla, right valve with tooth much reduced in size. Valves rounded apically. Uncus somewhat triangular, truncated apically, apex slightly hooked. Gnathos as two, fingerlike or acutely triangular outward facing extensions. Juxtal processes shorter than phallus, flattened, slightly curved, setae absent. Base of phallus with paired, short, rounded or pointed, diverging, backwards facing lobes. Phallus broad, widened mesally, with prominent, sharp or subtriangular dorsal ridge. Left edge of rolled phallus with triangular, ridge-like process, distal tip of phallus separated into two distinct, bent points. Vesica somewhat elongated, bag-like. **Female.** *Head*: As in male. *Thorax*: As in male. *Legs*: As in male. *Forewing dorsum*: Forewing length: 20.5 mm, n = 1. As in male but longer, only slightly broader, postmedial line barely kinked mesally. Faint antemedial line present, brown, bowed. *Forewing venter*: As in forewing dorsum but postmedial line mostly absent, antemedial absent, small black discal spot present. *Hindwing dorsum*: As in male. *Hindwing venter*: Following similar pattern as forewing venter except somewhat lighter, postmedial line faint. *Abdomen*: As in male but stouter. Sternite VIII as pair of elongated sclerotized bands widening toward anterior margin of VIII where they converge. *Genitalia*: (Fig. 93) n = 1. Tergite of VIII very broad, thin, weakly sclerotized, transparent, sac-like (see Fig. 93b). Robust apophyses anteriores shorter and wider than apophyses posteriores. Lamella antevaginalis relatively thin, weakly concave mesally. Ductus bursae elongate, tubular. Papillae anales somewhat rectangular, covered in setae.

##### Distribution

(Map 1). This new species is apparently endemic to southeastern Brazil in the states of Rio de Janeiro, São Paulo, and Santa Catarina. The habitat at each of the collecting localities falls within the Brazilian Atlantic Forest biome (IBGE 2004).

##### Etymology.

*Menevia magna* is named for the very broad valves of the male genitalia and also for the overall size, which is quite large on average for the *lantona* species-group.

##### Remarks.

*Menevia magna* appears to be closely related to the much more widespread *Menevia lantona* of northern and central South America. Based on our present understanding of the distribution of this species in São Paulo, Santa Catarina, and Rio de Janeiro, it can be reasonably inferred that *Menevia magna* is probably found in eastern Paraná as well.

### *lucara* species-group

Species of the *lucara* species-group are similar to those of the *lantona* species-group and includes *Menevia lucara*, *Menevia menapia* sp. n., and *Menevia mielkei* sp. n. Sexual dimorphism is more pronounced than in the previous group, but still not as strong as in the next two species-groups, with females having broader, more ovoid forewings than males. The size of the species belonging to this group are on the smaller side for the genus, being only slightly larger than those of the *lantona* group overall. Forewing shape is stout and triangular as in the *lantona* group, but with more acutely falcate forewings and more steeply curved postmedial lunules. The ground color is dark, generally slate gray with varying degrees of brown and red in the medial area. The lobes of the gnathos in this species-group are unique among the genus, rather than being flattened, cupped, triangular or subtriangular in shape, they are thick, usually upturned, well-sclerotized structures reminiscent of boxing gloves. The phallus is rather tubular and simple, with only one species having a noticeable dorsal phallic ridge.

#### Key to *lucara* species-group

**Table:** 

1	Forewing not acutely falcate, phallus without dorsal ridge. Central America to northern South America	**2**
–	Forewing very acutely falcate, narrow, phallus with dorsal ridge. Southeastern Brazil	***Menevia mielkei* sp. n.**
2	Juxtal processes pointed. Belize and Guatemala	***Menevia menapia* sp. n.**
–	Juxtal processes terminally spatulate. Northern South America	***Menevia lucara***

#### Menevia lucara

Taxon classificationAnimaliaLepidopteraMimallonidae

(Schaus, 1905)

Figs 18–23 , 76 , 94 ; Map 2

Cicinnus lucara Schaus, 1905: 328
Menevia lucara ; Schaus 1928: fig. ♂ 88f
Menevia lucara ; Forbes 1942
Menevia lucara ; Becker 1996
Menevia lucara ; Herbin and Mielke 2014: fig. ♂ 51

##### Type material.

**Holotype**, ♂: **FRENCH GUIANA**: St. Jean, Maroni, Fr. Guiana/ Collection Wm. Schaus/ *Perophora lucara* type Schaus/ Type No.: 8895 U.S.N.M./ USNM-Mimal: 1123/ St. Laurent diss.: 2-5-15:3/ (USNM) [examined]. No paratypes. Type locality: French Guiana: St. Jean du Maroni.

##### Additional specimens examined.

(60 ♂, 4 ♀ total) **BRAZIL: Amazonas**: 4 ♂, Hyutanahan [Huitanaã], Rio Purus: II.1922, III.1922, S.M. Klages, Carn. Mus. Accs. 6963, 7088, 8840, St. Laurent diss.: 2-5-15:5 (CMNH). 2 ♂, Nova Olinda, Rio Purus: V.1922, VI.1922, S.M. Klages, Carn. Mus. Acc. 7088, St. Laurent diss.: 2-5-15:9 (CMNH). 1 ♂, Miracema, Rio Purus: IV.1922, S.M. Klages, Carn. Mus. Acc. 6960, St. Laurent diss.: 2-7-15:1 (CMNH). 1 ♂, “en remontant l’Amazones de Teffé à Tonantins,” [between Tefé and Tonantins]: XI.1921, Dognin Collection, USNM-Mimal: 2584 (USNM). 1 ♂, 2 km. N. of Itacoatiara-Manaus Highway, 11 km. W. of Itacoatiara, “Canadian Fathers’ Pool”: 11.V.1972, E.G., I. & E.A. Munroe (CNC). 3 ♂, Reserva Ducke, km. 26 Manaus-Itacoatiara Highway: 16.V.1972, 20.IV.1972, E.G., I. & E.A. Munroe, St. Laurent diss.: 7-7-15:2 (CNC). 5 ♂, Fonte Boa: V.1906, VI.1906, S.M. Klages, Rothschild Bequest, BM 1939–1 (NHMUK). **Pará**: 1 ♂, Ponte Nova, Rio Xingu: Dognin Collection, USNM-Mimal: 2575 (USNM). 3 ♂, 1 ♀, Likely Belém: A.M. Moss (NHMUK). **COLOMBIA**: 1 ♂, Antioquia, Nari [Nare?] River: Collection Frank Johnson, USNM-Mimal: 2580, St. Laurent diss.: 2-5-15:6 (USNM). 1 ♂, Muzo, 400–800 m: Coll. Fassl, ex. Joicey Coll. Brit. Mus, 1925—157, BMNH(E) 1378765, St. Laurent diss.: 6-29-15:1 (NHMUK). 1 ♂, Villavicencio, 400 m: Coll. Fassl, ex. Joicey Coll. Brit. Mus, 1925—157 (NHMUK). 1 ♀, “Interior of Colombia”: Wheeler, ex. Joicey Coll. Brit. Mus, 1925—157, BMNH(E) 1378760 [abdomen missing, no dissection prep.] (NHMUK). 2 ♂, “W. Columb.”, Rio Dagua, 600–1000 m: “2–5,” W. Hopp S., St. Laurent diss.: 7-7-15:1 (MNHU). **ECUADOR**: 2 ♂, Esmeraldas, 27 km. W. Alto Tambo, 200 m, timber tract at forest edge: 19.VIII.1996, Jan Hillman, St. Laurent diss: 2-7-15:3, 9-7-14:2 (CMNH). **FRENCH GUIANA**: 13 ♂, St. Jean du Maroni: Collection Le Moult, ex. Dognin Collection, “topotype”, USNM-Mimal: 2578, St. Laurent diss.: 2-7-15:2 (USNM); VII-VIII.1904, E. Le Moult, Rothschild Bequest, BM 1939–1 (NHMUK); 1905–14, Rothschild Bequest, BM 1939-1 (NHMUK). 2 ♂, Mana River: V.1917, Acc. 6008, St. Laurent diss.: 2-5-15:4 (CMNH). 1 ♀, Kourou Forest: 25.VIII.1975, Mission M. Boulard and P. Pompanon, Muséum Paris, St. Laurent diss.: 5-22-15:1 (MNHN). 3 ♂, St. Laurent du Maroni: 1920–1932, ex. coll. L. & J. de Joannis (MNHN); XI, Collection Le Moult, ex. coll. Ed. Brabant, ex. Joicey Coll. Brit. Mus, 1925–157 (NHMUK); I.1906, E. Le Moult, Rothschild Bequest, BM 1939–1 (NHMUK). 1 ♂, Saül: 7.III.1978, Th. Porion (MNHN). **GUYANA**: 5 ♂, Potaro: V.1908, S.M. Klages, Rothschild Bequest, BM 1939–1 (NHMUK). 1 ♀, Bartica: W.J. Kaye, 1904-25, BMNH(E) 1378763, St. Laurent diss.: 7-4-15:1 (NHMUK). 1 ♂, Montsinery-Tonnegrande Nr. Tonnegrande [D5], N04°50.772', W52°31.165', 12 m: 11.III.2011, P. Sammut (RAS). **PANAMA**: 1 ♂, Darien vic. Cerro Pirre, Rancho Frio, N 08°01'11.3", W 077°43'57.0", 100 m: 19–24.VII.2013, J.R. MacDonald, St. Laurent diss.: 9-7-15:1 (MEM). **PERU**: 3 ♂, Carabaya, La Union, Huacamayo River, 2000 ft.: “wet s.,” XII.1904, G. Ockenden, Rothschild Bequest, BM 1939–1, BMNH(E) 1378764, St. Laurent diss.: 6-29-15:2 (NHMUK). **SURINAME**: 2 ♂, Moengo, Boven Cottica River: 25.V.1927, Cornell Univ. Lot 760, Sub 79, Lot 672, Sub 382, St. Laurent diss.: 12-10-13:1 (CUIC). 1 ♂, Aroewarwa Creek, Maroewym Valley: IV.1905, S.M. Klages, Rothschild Bequest, BM 1939–1 (NHMUK).

##### Diagnosis.

Both males and females are recognizable by their moderate size and gray ground color with pinkish highlights especially concentrated near the apical angle of the contrasting black postmedial line and near the anal angle of hindwings. The postmedial lunule is bright white and sharply curved outward toward the forewing margin, nearly forming a semicircle. The phallus/juxta combination is unique in the genus in that the phallus is smooth and cylindrical, without a dorsal phallic ridge. The terminal ends of the juxtal processes are spatulate, not pointed, and curved inward toward each other, near the terminal quarter of their length. The gnathos processes are highly distinct; they are thick, upturned, heavily sclerotized, somewhat boxing glove in shape.

##### Description.

**Male.** *Head*: Light gray, straw colored in old specimens, eyes large comprising about two-thirds of head area, eyes bordered posteriorly by dark brown collar of scales reaching labial palpi, labial palpi small, segments weakly defined ventrally due to ventral tufts, dorsally with darker scales contrasting with overall gray coloration. Scape and pedicel tufted. *Thorax*: As for genus. Grayish. *Legs*: As for genus. Tibial spurs thin apically, terminal third not scaled, especially ventrally, weakly hooked. *Forewing dorsum*: Forewing length: 16.5–21 mm, avg.: 17.3 mm, n = 22. Triangular, apical half of outer margin concave, apex falcate. Ground color gray with pink hue especially near apical point of postmedial line and throughout medial area, overall lightly speckled by dark petiolate scales. Discal spot faintly marked by light gray. Apex marked by black scales above apical dash. Straight postmedial line black or brown, usually strongly contrasting. Antemedial area lighter, submarginal area gray without pink hue, somewhat contrasting with medial area, postmedial lunule originating from apical dash, lunule follows postmedial line from apex to one third length of postmedial line where lunule sharply sweeps outward toward wing margin, forming acute angle with postmedial line. Antemedial line usually absent, if present, faint, brown, undulating. *Forewing venter*: As in forewing dorsum but postmedial line fainter, antemedial line absent, small black discal spot present. *Hindwing dorsum*: Somewhat rounded with margin weakly pointed mesally, anal angle accentuated, similar coloration and patterning as forewings, vague postmedial lunule originating near anterior margin, sweeping outward to marginal point, antemedial line absent, postmedial line straight or slightly curved, especially near anterior wing margin. *Hindwing venter*: Following similar pattern as forewing venter, but slightly lighter, discal mark absent. *Abdomen*: As for genus. Coloration a continuation of grayish thoracic color. Midventral stripe absent. *Genitalia*: (Fig. 76) n = 14. Tegumen subtriangular, not constricted near base of gnathos. Vinculum broad, somewhat quadrate ventrally. Valves simple, relatively narrow, saccular and costal edges of valves with sharp tooth proximal to transtilla. Valves rounded apically. Uncus bottle-shaped, apex quadrate. Gnathos as two prominent, thick, heavily sclerotized, boxing glove shaped, upturned, outward facing extensions of varying width, occasionally broken dorsally. Juxtal processes roughly phallus length, heavily sclerotized, curving inward toward each other. Processes spatulate distally, setae absent. Base of phallus with robust, paired, somewhat elongated, rounded, diverging lobes, angled ventrally. Phallus simple, cylindrical, thin, but expanded distally. Left edge of rolled phallus simple, ridge-like process absent, distal tip of phallus separated into two distinct, often curled, points of varying length. Vesica elongated. **Female.** *Head*: As in male, labial palpi slightly longer. *Thorax*: As in male. *Legs*: As in male. *Forewing dorsum*: Forewing length: 15–20.5 mm, avg. 18.5 mm, n = 3. As in male but broader, less falcate, pinkish hue more prominent, postmedial line bowed out slightly mesally. *Forewing venter*: As in forewing dorsum but postmedial line fainter, antemedial line absent, small black discal spot present. *Hindwing dorsum*: As in male but slightly more rounded, broader. *Hindwing venter*: Following similar pattern as forewing venter except lighter. *Abdomen*: As in male but stouter. Sternite of VIII as pair of elongated sclerotized bands converging near anterior margin of VIII forming a “V”. *Genitalia*: (Fig. 94) n = 2. Tergite of VIII smoothly curved, very wide, with rounded edges, membranous gap mesally. Apophyses anteriores slightly shorter than apophyses posteriores. Lamella antevaginalis bent mesally, forming sharp “V” with ostium bursae at apex. Ductus bursae elongate. Papillae anales subtriangular when viewed ventrally, base widened, covered in relatively long setae especially at apex.

##### Distribution

**Map 2.**
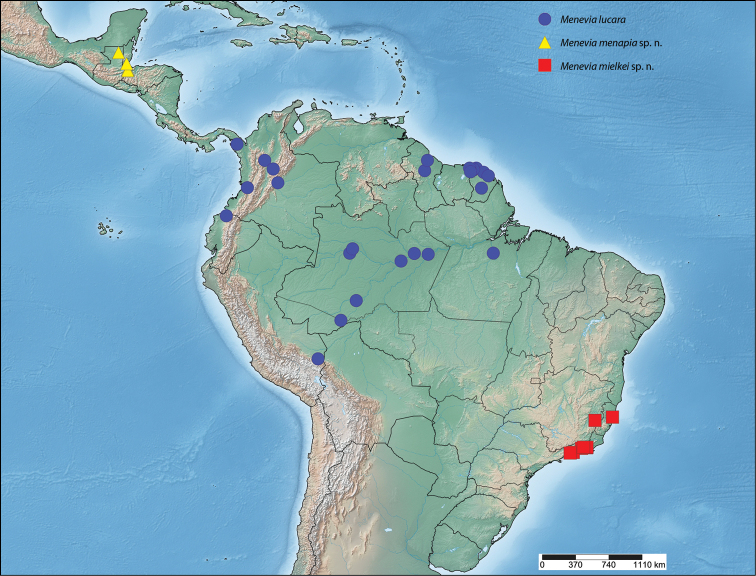
*Menevia lucara* species-group.

(Map 2). *Menevia lucara* is found throughout the Amazonian rainforest in the Guianas, Suriname, the Brazilian states of Pará and Amazonas, as well as in western and central Colombia, Panama, northwestern Ecuador, and southern Peru.

##### Remarks.

Like *Menevia lantona*, *Menevia lucara* is a wide-ranging Amazonian species found throughout northern South America and is sympatric with *Menevia lantona* throughout most of its range.

Some geographical variation has been noted among the material examined. Specimens from Panama and Colombia (Figs 20 and 21 respectively) average slightly larger than those from the rest of the species range. Similarly, *Menevia lantona* showed parallel geographic variation, with specimens from Colombia, and some from Panama also being larger on average. Additional geographic variation is present in the shape of the phallus. In some of the examined specimens from Colombia, and one from Ecuador, the phallus is slightly thicker and more robust overall than in specimens from other Amazonian localities. Also, the apical tips of the phallus are very short in specimens from French Guiana, Guyana, Suriname, and Peru, whereas they are longer in specimens from Ecuador and those from some Colombian localities.

**Figures 18–29.**
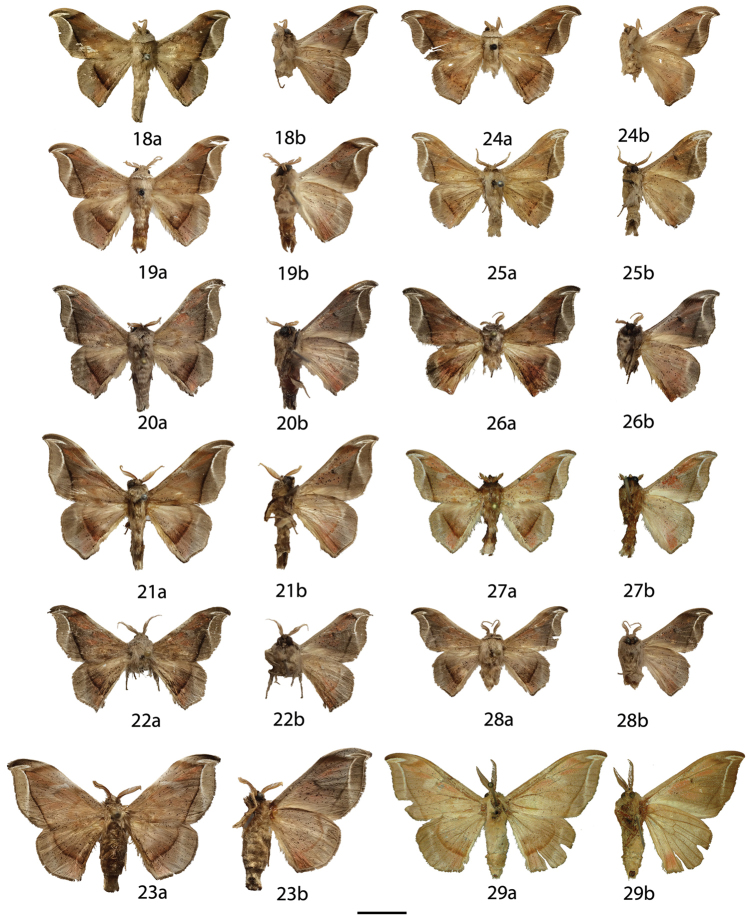
*Menevia lucara* species-group adults, **a** recto, **b** verso. **18** *Menevia lucara* holotype ♂, French Guiana, St. Jean du Maroni (USNM) **19** *Menevia lucara* ♂, Brazil, Nova Olinda, Rio Purus (CMNH) **20** *Menevia lucara* ♂, Panama, Darien vic. Cerro Pirre, Rancho Frio (MEM) **21** *Menevia lucara* ♂, Colombia, Rio Dagua, 600-1000 m (MNHU) **22** *Menevia lucara* ♂, Ecuador, Esmeraldas, 27 km W Alto Tambo, 200 m (CMNH) **23** *Menevia lucara* ♀, French Guiana, Kourou Forest (MNHN) **24** *Menevia menapia* holotype ♂, Guatemala, Cayuga (USNM) **25** *Menevia menapia* paratype ♂, Belize, Punta Gorda (NHMUK) **26** *Menevia menapia* paratype ♂, Guatemala, Izabal, Finca la Firmeza (CUIC) **27** *Menevia mielkei* holotype ♂, Brazil, Minas Gerais, Estação Biológica de Caratinga, Caratinga [photo courtesy CGCM] (DZUP) **28** *Menevia mielkei* paratype ♂, Brazil, Rio de Janeiro, Cachoeiras de Macacu, 700 m (USNM) **29** *Menevia mielkei* paratype ♀, Brazil, Rio de Janeiro, Sahy-Ramal de Mangaratiba [photo courtesy CGCM] (DZUP). Scale bar = 1 cm.

#### Menevia menapia sp. n.

Taxon classificationAnimaliaLepidopteraMimallonidae

http://zoobank.org/290C69E5-B0E0-4C36-B4B6-57D71ECAB807

Figs 24–26 , 77 ; Map 2

##### Type material.

**Holotype**, ♂: **GUATEMALA**: Cayuga Guat/ June/ Schaus and Barnes coll/ USNM-Mimal: 2565/ St. Laurent diss.: 9-7-14:1/ HOLOTYPE male *Menevia menapia* St Laurent and Dombroskie, 2016 [handwritten red label] (USNM). Type locality: Guatemala: Cayuga.

**Paratypes**, 8 ♂: **BELIZE**: 2 ♂, Punta Gorda, Brit. Honduras: VI.1933, J.J. White, Rothschild Bequest BM 1939-1, St. Laurent diss.: 6-29-15:3, BMNH(E) 1378758 (NHMUK); same data as previous Belize specimen, but no BMNH(E) number and not dissected, this specimen is missing the abdomen with no genitalia prep. (NHMUK). **GUATEMALA**: 3 ♂, Cayuga: Schaus and Barnes coll., Dognin Collection, “*Cicinnus lucara* Schaus 1920, Schaus,” USNM-Mimal: 2579 (USNM); VI, Schaus and Barnes coll., USNM-Mimal: 2564 (USNM); IV, “*Cicinnus lucara* I Schaus,” Carn. Mus. Acc. 6540, St. Laurent diss.: 2-5-15:7 (CMNH). 2 ♂, Tikal: 10.I.1980, R. Holland (AMNH); 11.I.1980, R. Holland, UV light, St. Laurent diss. 2-7-15:4 (AMNH). 1 ♂, Izabal, Finca la Firmeza 15.407, -88.696: 1.III.2014, J.J. Dombroskie, T. McCabe, J. Monzón, MV/UV light, St. Laurent diss.: 2-5-15:8 (CUIC). – All paratypes with the following yellow label: PARATYPE male *Menevia menapia* St Laurent and Dombroskie, 2016.

##### Diagnosis.

Externally *Menevia menapia* is nearly identical to *Menevia lucara*, albeit this new species is slightly smaller on average. The most significant differences in external characters are present in the postmedial lunules of the fore- and hindwings. The postmedial lunule is less sharply swept to the forewing margin in *Menevia menapia*, and on the hindwing the lunule is more distinct, a brighter white rather than extremely faded as in *Menevia lucara*. The male genitalia of *Menevia menapia* are recognizable by the weaker sclerotization; smaller, slightly thinner processes of the gnathos, and the sharp, rather than spatulate, apices of the juxtal processes. Furthermore, the lobes at the base of the phallus are much shorter and stouter than in *Menevia lucara*. Geography is perhaps the easiest way to differentiate *Menevia menapia* from other species in the *lucara* species-group, as it is the only representative of the group from Central America north of Panama.

##### Description.

**Male.** *Head*: Gray, fading to straw colored in old specimens, eyes large comprising about two-thirds of head area, eyes bordered posteriorly by dark brown collar of scales reaching labial palpi, labial palpi small, segments weakly defined ventrally. Scape and pedicel tufted. *Thorax*: As for genus. Gray fading to straw in old specimens. *Legs*: As for genus. Tibial spurs thin apically, terminal third not scaled, weakly hooked. *Forewing dorsum*: Forewing length: 15–17 mm, avg.: 16 mm, n = 7. Triangular, apical half of outer margin concave, apex falcate. Ground color gray with pink hue medially, overall lightly speckled by dark petiolate scales. Discal spot faintly marked by light gray. Apex marked by black scales above apical dash. Weakly concave postmedial line black, contrasting. Antemedial area lighter, submarginal area gray without pink hue, contrasting with pinkish medial area, postmedial lunule originating from apical dash, lunule follows postmedial line from apex to one third length of postmedial line where lunule sweeps outward toward wing margin, roughly forming 45 degree angle with postmedial line. Antemedial line faint or absent, if present, brown, undulating. *Forewing venter*: As in forewing dorsum but postmedial line fainter, antemedial line absent, small black discal spot present. *Hindwing dorsum*: Rounded with margin weakly pointed mesally, anal angle accentuated, similar coloration and patterning as forewings, postmedial lunule present, originating near anterior margin, sweeping outward and fading to marginal point, antemedial line absent, postmedial line straight except near anterior margin. *Hindwing venter*: Following similar pattern as forewing venter. *Abdomen*: As for genus. Coloration a continuation of grayish thoracic color. Midventral stripe absent. *Genitalia*: (Fig. 77) n = 5. Tegumen subtriangular, not constricted near base of gnathos. Vinculum broad, somewhat quadrate ventrally. Valves simple, relatively narrow, saccular and dorsal edges of valves with sharp tooth proximal to transtilla. Valves rounded or somewhat quadrate apically. Uncus somewhat triangular, apex quadrate. Gnathos as two sclerotized, somewhat boxing glove shaped, upturned, outward facing extensions. Juxtal processes roughly phallus length, not inwardly curved, parallel, curved toward phallus apex, flattened, lightly covered in short setae, pointed apically. Base of phallus with paired, stout, rounded, diverging lobes angled ventrally. Phallus simple, cylindrical, thin but somewhat engorged distally. Left edge of rolled phallus simple, without ridge like process, distal tip of phallus separated into two distinct points of varying length. Vesica bag-like. **Female.** Unknown.

##### Distribution

(Map 2). *Menevia menapia* is so far known only from the Petén and Izabal Departments of eastern Guatemala and an adjacent area of Belize.

##### Etymology.

*Menevia menapia* is named for the likely derivation of *Menevia*, Menapia. The etymology of Menapia, however, is less clear and may refer to an ancient Roman settlement supposed to have existed in Pembrokeshire, Wales or to a settlement inhabited by the Menapii people in Belgica.

##### Remarks.

This species, although very similar in external appearance and genitalia characteristics to the South American representatives of the *lucara* species-group, especially the Amazonian *Menevia lucara*, is separated by well over 2000 km land distance. Because of the extreme allopatry of *Menevia menapia*, it is surprising that this new species differs so little from the wide-ranging, South American *Menevia lucara*. Despite these similarities, the allopatry combined with the overall slightly smaller size, minor external differences, and the distinct genitalia differences, warrant the separation of these two similar species.

#### Menevia mielkei sp. n.

Taxon classificationAnimaliaLepidopteraMimallonidae

http://zoobank.org/A8588B6A-EE9D-4D08-8ADD-ADAF7C90AA45

Figs 2 , 27–29 , 78 ; Map 2

##### Type material.

**Holotype**, ♂: **BRAZIL: Minas Gerais**: 29-I--3-II-2003, Estação Biológica de Caratinga, Caratinga, MG, 400 m, Mielke & Casagrande leg./ OM 61.563/ CGCM diss.: OM 61.563/ HOLOTYPE male *Menevia mielkei* St Laurent and Dombroskie, 2016 [handwritten red label] (donated to DZUP by OM). Type locality: Brazil: Minas Gerais: Caratinga.

**Paratypes**, 13 ♂, 1 ♀: **BRAZIL: Espírito Santo**: 1 ♂, Linhares, 40 m: 05–09.IV.1992, V.O. Becker col., USNM-Mimal: 2345, St. Laurent diss.: 2-5-15:12 (USNM). **Rio de Janeiro**: 4 ♂, Angra-Jussaral: 25.XI.1935, coll. D’Almeida, No. 19.179 (DZUP); 20.II.1936, 24.II.1936, D’Almeida, Oiticica & A. Costa, ex. coll. D’Almeida, No. 19.176, 19.177, 19.178, DZ 32.702–32.705 (DZUP). 1 ♂, Petrópolis, 650 m: 10–20.X.1985, V.O. Becker col., col. Becker 64840, USNM-Mimal: 2344, St. Laurent diss.: 2-5-15:10 (USNM). 2 ♂, Petrópolis: 7.IX.1928, 12.XI.1928, Gagarin leg., ex. col. Gagarin, DZ 32.706–32.707 (DZUP). 3 ♂, Teresópolis, Barreira: 11.X.1955, 15.X.1955, 18.X.1955, ex. col. Gagarin, DZ 32.708–32.710 (DZUP). 1 ♂, Cachoeiras de Macacu, 700 m: 23.I.1998, V.O. Becker col., col. Becker 113168, USNM-Mimal: 2036, St. Laurent diss.: 2-5-15:11 (USNM). 1 ♂, Cachoeiras de Macacu, Boca do Mato: 18.I.1998, N. Tangerini col., ex. coleção Nirton Tangerini, DZ 32.711 (DZUP). 1 ♀, Sahy-Ramal de Mangaratiba [Saí]: 25.X.1932, coll. Ferr. d’Almeida, ex. coll. D’Almeida, No. 19.180, DZ 32.712 (DZUP). – All paratypes with the following yellow label: PARATYPE male/female *Menevia mielkei* St Laurent and Dombroskie, 2016.

##### Diagnosis.

Externally *Menevia mielkei* is nearly identical to both *Menevia lucara* and *Menevia menapia*. The forewings of *Menevia mielkei* are slightly narrower than in the other two species, most notably by the somewhat more acute apices. Genitalia are very useful however, in the diagnosis of *Menevia mielkei*. In *Menevia mielkei*, the uncus is more slender, the processes of the gnathos are extremely atrophied and thin, the phallus has a distinct dorsal ridge, the processes of the juxta are nearly vertically extended above the base of the phallus, not curved over its length, and the valves lack the saccular edge tooth. Furthermore, the lobes at the base of the phallus are much shorter and stouter than in *Menevia lucara*, and in that way are more similar to *Menevia menapia*.

##### Description.

**Male.** *Head*: Light gray, eyes large comprising about two-thirds of head area, eyes bordered posteriorly by somewhat reduced dark brown collar of scales reaching labial palpi, labial palpi small, segments weakly defined ventrally. Scape and pedicel tufted. *Thorax*: As for genus. Grayish. *Legs*: As for genus. Tibial spurs thin apically, terminal third not scaled, especially ventrally, weakly hooked. *Forewing dorsum*: Forewing length: 16–17 mm, avg.: 16.6 mm, n = 4. Triangular, apical half of outer margin deeply concave, slightly convex near tornus, apex acutely falcate. Ground color gray with pinkish-red to salmon hue especially near apical point of postmedial line and near discal region, overall lightly speckled by dark petiolate scales. Discal spot faintly marked by light gray. Apex marked by black scales above apical dash. Postmedial line straight, black-brown. Antemedial area lighter than medial area, submarginal area gray without pink hue, postmedial lunule originating from apical dash, lunule follows postmedial line from apex to roughly one third length of postmedial line where lunule sharply sweeps outwards toward wing margin, forming acute angle with postmedial line. Antemedial line very faint, brown, undulating. *Forewing venter*: As in forewing dorsum but postmedial line fainter, pinkish-red hue concentrated near costa and discal region, antemedial line absent, small black discal spot present. *Hindwing dorsum*: Rounded with margin weakly pointed mesally, anal angle accentuated, similar coloration and patterning as forewings, small black discal mark usually present, very vague postmedial lunules originating near anterior margin sweeping outward to marginal point, antemedial line absent, postmedial line straight or slightly concave. *Hindwing venter*: Following similar pattern as forewing venter, pinkish-red hue concentrated in anal region. *Abdomen*: As for genus. Coloration a continuation of grayish thoracic color. Midventral stripe absent. *Genitalia*: (Fig. 78) n = 4. Tegumen subtriangular, quadrate at base. Vinculum broad, quadrate ventrally. Valves simple, relatively narrow, saccular edge smooth. Valves rounded apically. Uncus elongated, apex quadrate. Gnathos as two thin, atrophied, lightly sclerotized, outward facing extensions. Juxtal processes angled nearly perpendicular to dorsal surface of phallus. Juxtal processes sharply tipped, lightly covered in setae. Base of phallus with paired, backwards facing, rounded, diverging lobes. Phallus thick, expanded mesally. Left edge of rolled phallus forms distinct ridge of varying shape, always covered in setae, distal tip of phallus separated into two distinct points of varying length. Vesica elongate, sac-like. **Female.** *Head*: As in male. *Thorax*: As in male. *Legs*: As in male. *Forewing dorsum*: Forewing length: about 21 mm, n = 1. As in male but broader, less falcate, pinkish hue more prominent, postmedial line bowed out slightly mesally. *Forewing venter*: As in forewing dorsum but postmedial line fainter, antemedial line absent, small black discal spot present. *Hindwing dorsum*: As in male but slightly more rounded, broader. *Hindwing venter*: Following similar pattern as forewing venter except lighter. *Abdomen*: As in male but stouter. *Genitalia*: Not examined.

##### Distribution.

*Menevia mielkei* is found in a small region of southeastern Brazil in the states of Rio de Janeiro, Espírito Santo, and adjacent eastern Minas Gerais. Whether or not this species is more widely distributed in the Brazilian Atlantic Forest is yet to be determined.

##### Etymology.

*Menevia mielkei* is named after Carlos G. C. Mielke who provided extensive support and data throughout the process of writing this revision.

##### Remarks.

Like *Menevia menapia*, *Menevia mielkei* is very similar to the wide-ranging *Menevia lucara*. Both *Menevia menapia* and *Menevia mielkei* are apparently restricted in distribution, both species being widely allopatric with *Menevia lucara*. Of the three species in the *lucara* species-group, *Menevia mielkei* has the most distinct genitalia characters as outlined in the diagnosis. The dorsal ridge of the phallus is a common character in many other species of *Menevia*, but within the *lucara* species-group, this ridge is only present in *Menevia mielkei*.

#### *ostia* species-group

The following group contains three species, *Menevia ostia* comb. n., *Menevia parostia* comb. n., and *Menevia pallida*. The former two species were assigned to the genus *Pamea* Walker, 1855 and the latter species was recently described in *Menevia* (Herbin and Mielke 2014). The species belonging to the *ostia* species-group are appropriately placed in *Menevia* due to the presence of the white postmedial lunule and the complex genus-specific male genitalia. In this species-group, the sexual dimorphism is pronounced. The species belonging to the *ostia* species-group are similar in size to larger species of the *lantona* and *lucara* species-groups, although those of the *ostia* species-group average slightly larger. The females of this group, namely those of *Menevia ostia*, can be quite large for the genus. The forewing shape of females are characteristic of this species-group, males however, are much more similar to those of the *lantona* group. Specifically, the forewings are triangular in both species-groups and only weakly falcate, especially in the *ostia* species-group. The species belonging to the *ostia* group are light yellow to dark yellow or somewhat gold, without a brownish cast to the medial area as in the *lantona* species-group. The stark yellowish coloration of the medial area is highly contrasting with the gray submarginal area, which is typical of the genus. The male genitalia are characteristic of this species-group; the *ostia* group is the only group in which every examined specimen has a prominent phallic ridge. The paired gnathos is similar to that of the *lantona* group, but in the *ostia* group it is more spade-shaped rather than simply triangular.

##### Key to *ostia* species-group

**Table:** 

1	Coloration washed out yellow-tan, hindwing with very faint zigzagged lunule; females: hindwing postmedial line closer to wing margin than to middle of wing	**2**
–	Coloration more gold yellow-orange, hindwing with usually prominent zigzagged lunule; females: hindwing postmedial line closer to thorax than to middle of wing. Distribution wide but usually in wet forests from Costa Rica to throughout Amazonia and into the Brazilian Atlantic Forest	***Menevia ostia* comb. n.**
2	Female forewing less than 21 mm, male unknown	***Menevia parostia* comb. n.**
–	Female forewing greater than or equal to 21 mm. Brazilian Cerrado, possibly also south to Paraguay	***Menevia pallida***

##### Menevia ostia

Taxon classificationAnimaliaLepidopteraMimallonidae

(Druce, 1898) comb. n.

Figs 4 , 5 , 30–33 , 36 , 37 , 79–81 , 95 ; Map 3

Perophora ostia Druce, 1898: 447; Tab. LXXXVIII, fig. 18 ♀
Pamea ostia ; Schaus 1928: fig. ♀ 88g
Pamea ostia ; Becker 1996

###### Type material.

**Holotype**, ♀: **PANAMA**: Chiriqui [Chiriquí]/ 855/ Coll. Staudinger/ Type/ *Perophora ostia* ♀ type Druce/ Coll. Staudinger II.1178./ St. Laurent diss.: 7-14-15:1/ (MNHU) [examined]. Type locality: Panama: Chiriquí.

###### Additional specimens examined.

(31 ♂, 15 ♀ total) **BRAZIL: Espírito Santo**: 9 ♂, Linhares, 40 m: 20–29.II.1992, 06.III.1993, 25–30.I.1998, V.O. Becker Col., Col. Becker 80930, 82945, 113493, USNM-Mimal: 2053, 2054, 2310–2316, St. Laurent diss.: 3-24-15:9, 3-24-15:10, 3-24-15:11, 7-14-14:5, 7-14-15:6 (USNM). **Minas Gerais**: 1 ♂, Estação Biológica de Caratinga, Caratinga, 400 m: 29.I.2003–3.II.2003, Mielke & Casagrande leg. (OM). **Pará**: 2 ♀, Likely Belém: A.M. Moss, Rothschild Bequest 1939–1, BMNH(E) 1377143 (NHMUK). **Rio de Janeiro**: 3 ♂, Petrópolis: 7.IV.1928, 6.IX.1959, 15.X.1963, Gagarin leg., ex. coll. Gagarin (DZUP). **COSTA RICA** (all Costa Rican specimens ex. D. H. Janzen & W. Hallwachs, Área de Conservación Guanacaste): **Alajuela**: 1 ♂, 1 ♀, Rio Blanco Abajo, 10.90037, -85.37254, 500 m: Voucher ♂: 05-SRNP-7880 [barcoded], collected: 18.XII.2005, emerged: 15.III.2006, food plant: *Terminalia oblonga* (USNM); Voucher ♀: 12-SRNP-4882 [barcoded], collected: 15.XI.2012, emerged: 12.V.2013, food plant: *Terminalia oblonga* (USNM). 1 ♂, Casa Leiva, 10.94314, -85.31808, 454 m: 17.IX.2009, light trap, Voucher: 09-SRNP-108159, St. Laurent diss.: 4-20-15:13 (USNM). 1 ♂, “Jabalina, Manta Pizote,” 10.97325, -85.31542, 288 m: 30.VIII.2008, light trap, Voucher: 08-SRNP-105449 (USNM). 1 ♂, Gallinazo, 11.01825, -85.37199, 360 m: Voucher: 11-SRNP-65684 [barcoded], collected: 21.VII.2011, emerged: 22.VIII.2011, food plant: *Terminalia amazonia* (USNM). 2 ♂, 1 ♀, Puente Palma, 10.9163, -85.37869, 460 m: Voucher ♂: 03-SRNP-34660 [barcoded], collected: 1.XII.2003, emerged: 16.I.2004, food plant: *Terminalia oblonga* (USNM); Voucher ♂: 03-SRNP-34659, collected: 1.XII.2003, emerged: 13.I.2004, food plant: *Terminalia oblonga* (USNM); Voucher ♀: 03-SRNP-34661, collected: 1.XII.2003, emerged: 20.I.2004, food plant: *Terminalia oblonga* (USNM). 1 ♂, 1 ♀, Tomatera: Voucher ♂: 12-SRNP-65485 [barcoded], collected: 16.VII.2012, emerged: 15.IX.2012, food plant: *Terminalia amazonia*, St. Laurent diss.: 4-20-15:12 (USNM); Voucher ♀: 12-SRNP-65488 [barcoded], collected: 16.VII.2012, emerged: 8.IX.2012, food plant: *Terminalia amazonia*, St. Laurent diss.: 4-20-15:11 (USNM). **Guanacaste**: 1 ♂, Sendero Puertas, 11.01087, -85.48817, 400 m: Voucher: 03-SRNP-18623, collected: 5.VIII.2003, emerged: 28.IX.2003, food plant: *Terminalia amazonia*, St. Laurent diss.: 7-14:15:3 (USNM). 1 ♂, 1 ♀, Pasmompa, 11.01926, -85.40997, 440 m: Voucher ♂: 07-SRNP-31642 [barcoded], collected: 8.III.2007, emerged: 1.VI.2007, food plant: *Terminalia amazonia* (USNM); Voucher ♀: 07-SRNP-31860 [barcoded], food plant: *Terminalia amazonia*, St. Laurent diss.: 4-20-15:8 (USNM). 1 ♂, Monte Cristo, 11.01373, -85.42531, 525 m: Voucher: 10-SRNP-21610 [barcoded], collected: 17.VII.2010, emerged: 14.IX.2010, food plant: *Terminalia amazonia* (USNM). 1 ♂, Tajo Angeles, 10.86472, -85.41531, 540 m: Voucher: 10-SRNP-4309 [barcoded], collected: 8.VIII.2010, emerged: 9.IX.2010, food plant: *Terminalia oblonga*, St. Laurent diss.: 7-14-15:2 (USNM). 1 ♂, Metereologico, 11.00199, -85.46166, 590 m: Voucher: 10-SRNP-21714 [barcoded], collected: 26.VII.2010, emerged: 29.VIII.2010, food plant: *Terminalia amazonia*, St. Laurent diss.: 4-20-15:14 (USNM). 1 ♂, 1 ♀, Casa Roberto, 11.01095, -85.42094, 520 m: Voucher ♂: 03-SRNP-20585 [barcoded], collected: 11.VIII.2003, food plant: *Terminalia amazonia* (USNM); Voucher ♀: 03-SRNP-20690, collected: 11.VIII.2003, emerged: 17.V.2004, food plant: *Terminalia amazonia* (USNM). 1 ♀, Mena Central, 11.02991, -85.45364, 345 m: Voucher: 01-SRNP-24319 [barcoded], collected: 29.XI.2001, St. Laurent diss.: 4-20-15:9 (USNM). 1 ♀, Sendero Rotulo, 11.01355, -85.42406, 510 m: Voucher: 04-SRNP-34126 [barcoded], collected: 27.VII.2004, emerged: 5.IX.2004, food plant: *Terminalia amazonia* (USNM). 1 ♀, Camino Mangos, 11.00766, -85.47926, 480 m: Voucher: 03-SRNP-19176, collected: 12.VIII.2003, emerged: 13.IX.2003, food plant: *Terminalia amazonia* (USNM). 1 ♀, Maderos, 11.00494, -85.47491, 510 m: Voucher: 11-SRNP-20049 [barcoded], collected: 4.I.2011, emerged: 15.V.2004, food plant: *Terminalia amazonia*, St. Laurent 4-20-15:10 (USNM). **FRENCH GUIANA**: 1 ♂, Sinnamary, Piste a St. Elie, km. 24, 50 m: 20.II.1991, at lights, coll. C. Snyder, St. Laurent diss.: 3-24-15:6 (AMNH). 1 ♂, Nouveau Chantier: Collection Le Moult, Dognin Collection, USNM-Mimal: 2572, St. Laurent diss.: 3-24-15:7 (USNM). 1 ♂, Piste Coralie PK 2: IV.1993, J. Navatte H. de Toulgoët (MNHN). 1 ♀, Godebert Maroni: VII, Collection Le Moult, Ex. Coll. Ed. Brabant 1920, ex. Joicey Coll., Brit. Mus. 1925–157, BMNH(E) 1377145 (NHMUK). 1 ♀, no additional locality data: Collection C. Bar, Ex. Oberthür Coll., Brit. Mus. 1927–3, BMNH(E) 1377140 (NHMUK). **PERU**: 1 ♀, Junín, Chanchamayo: I-VIII.1901, Hoffmann, Rothschild Bequest 1939–1, BMNH(E) 1377144 (NHMUK). **SURINAME**: 1 ♀, Paramaribo: 7.I.1955, Rf. [reared] *Terminalia catappa* L., J.B.M van Dinther, USNM-Mimal: 2612, St. Laurent diss.: 4-20-15:5 (USNM). **VENEZUELA**: 1 ♂, Aragua, Rancho Grande, 1100 m: 22-31.VII.1967, R.W. Poole, USNM-Mimal: 2739, St. Laurent diss.: 3-24-15:8 (USNM). **UNKNOWN**: 1 ♂ specimen without locality label, but bears label with red “1” written in colored pencil, there was a similar label on an examined female from Suriname, USNM-Mimal: 2613, St. Laurent diss.: 7-14-15:4 (USNM).

###### Diagnosis.

*Menevia ostia* can be differentiated from all other species-groups by the gold-yellow to pale yellow ground color with the contrasting silvery gray submarginal area with a small white accessory mark near the tornus. These traits combined with overall very weakly falcate forewings, distinguish *Menevia ostia* from the similar *Menevia lantona* but not necessarily from the similar *Menevia pallida* and *Menevia parostia* comb. n. to be diagnosed below. The width of the submarginal area of the hindwings of female *Menevia ostia* is wider than in both *Menevia pallida* and *Menevia parostia* females. The postmedial line is situated at about midway along the length of the hindwing in *Menevia ostia*, but is slightly closer to the wing margin in the other two species. Male genitalia are distinct, except when in comparison with *Menevia pallida*, in that the apical end of the phallus is sharply downturned and the dorsal ridge of the phallus is highly variable, though always present. In females, the lateral portion of the prominently sclerotized VIII has appendicular apophyses dorsolaterally in addition to the apophyeses anteriores, distinguishing the females from a questionable female of *Menevia pallida*, where the appendicular apophyses are absent.

###### Description.

**Male.** *Head*: Straw or tan-gold colored, eyes bordered posteriorly by dark brown collar of scales reaching labial palpi, labial palpi small, segments somewhat well defined ventrally, dorsally with darker scales contrasting with overall lighter coloration. Scape and pedicel weakly tufted. *Thorax*: As for genus. Gold to pale, fading to straw. *Legs*: As for genus. Tibial spurs thin apically lightly to almost completely scaled. *Forewing dorsum*: Forewing length: 12.5–18.5 mm, avg.: 16.8 mm, n = 23. Triangular, apical half of outer margin weakly concave, apex slightly falcate. Ground color gold-yellow to pale yellow, overall very lightly speckled by dark petiolate scales. Discal spot faintly marked by silvery white. Apex marked by black scales above apical dash. Black postmedial line slightly concave, sometimes weakly undulating. Submarginal area silvery gray, postmedial lunule originating from near where apical dash meets postmedial line, white mark follows postmedial line from apex to one quarter length of postmedial line where mark smoothly curves outwards toward wing margin becoming diffuse, forming acute angle with postmedial line. White accessory mark present near tornus. Antemedial line very faint or absent, if present, brown, undulating. *Forewing venter*: As in forewing dorsum but postmedial line fainter, convex near tornus, antemedial line absent, small black, somewhat rounded or elongated discal spot present. *Hindwing dorsum*: Rounded, anal angle weakly accentuated, similar coloration and patterning as forewings, postmedial lunule vague or well defined, zigzagged, originating near anterior margin, following curvature of wing margin, not steeply swept to margin, antemedial area lighter, postmedial line straight, sometimes undulating near anal angle. *Hindwing venter*: Following similar pattern as forewing venter but lighter, discal mark absent, marginal area usually browner than surrounding area. *Abdomen*: As for genus. Coloration a continuation of thoracic color. Midventral stripe absent. *Genitalia*: (Figs 79–81) n = 14. Tegumen subtriangular. Vinculum narrow, somewhat quadrate. Valves asymmetrical, moderate in width, saccular edge of left valve with large triangular tooth proximal to transtilla, right valve with tooth much reduced in size to nearly absent, both valves with smaller mesal costal tooth immediately above saccular edge teeth. Valves somewhat indented mesally, rounded apically. Uncus teardrop-shaped, truncated apically, base variable in width, apex somewhat hooked. Gnathos as two, flattened, spade-shaped outward facing flaps, bent toward each other, tips nearly converging. Juxtal processes shorter than phallus, thin, flattened, slightly curved, smooth. Base of phallus with paired, rounded, diverging, backwards facing fingerlike lobes. Phallus variable, broad, dorsal ridge of varying shape. Left edge of phallus forms distinct ridge, either a rounded or rectangular hump mesally or extended along phallus length but always quadrate anteriorly forming distinct edge, distal tip of phallus downturned, separated into two distinct, bent points. Vesica elongated. **Female.** *Head*: As in male but scales more grayish, labial palpi longer, thinner, dorsally covered in fewer dark scales. *Thorax*: As in male. *Legs*: As in male but tibial spurs broader, more triangular. *Forewing dorsum*: Forewing length: 17.5–26 mm, avg.: 22.8 mm, n = 11. Coloration and patterning as in male but maculation stronger, silvery discal mark wider, wing shape broader, more elongate, ovoid. *Forewing venter*: As in forewing dorsum but usually darker, markings subdued, postmedial line much fainter, bent near tornus; antemedial line not present; elongate black discal spot present. *Hindwing dorsum*: As in male but broader, postmedial lunule proportionally larger, zigzagged pattern more obvious. Postmedial line situated midway along length of wing. *Hindwing venter*: Following similar pattern as forewing venter but lighter. *Abdomen*: As in male but stouter. Sternite of VIII as pair of elongated sclerotized bands sometimes widening toward posterior margin of VIII converging near anterior margin. Sclerotized bands may be parallel or bowed out midway along length. *Genitalia*: (Fig. 95) n = 6. VIII prominently sclerotized laterally, with appendicular apophyses dorsolaterally in addition to apophyeses anteriores. Tergite of VIII arch-like, converging mesally to form posteriorly directed point. Apophyses anteriores slightly shorter than apophyses posteriores. Lamella antevaginalis as wide, semicircular, sclerotized band. Ductus bursae short. Papillae anales rectangular or subtriangular when viewed ventrally, covered in short setae.

###### Distribution

**Map 3.**
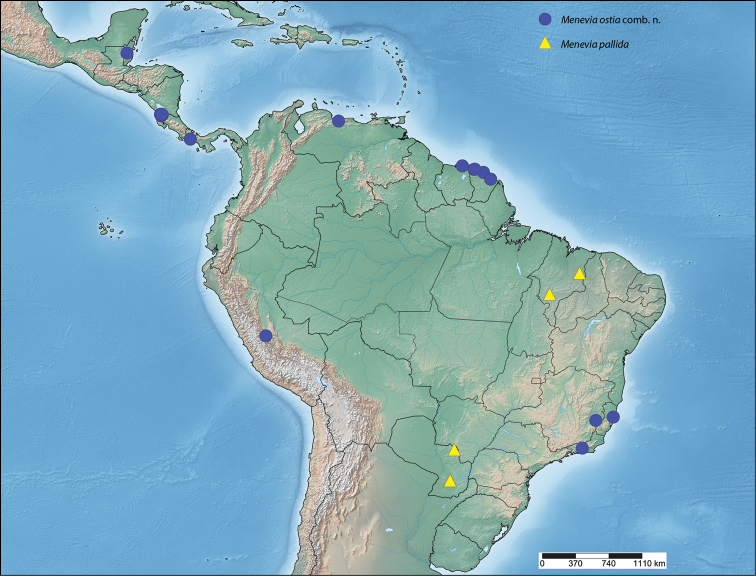
*Menevia ostia* species-group.

(Map 3). *Menevia ostia* is a widespread species, found in wet forests from Costa Rica and Panama, Peru, Suriname, French Guiana and the Brazilian Amazon in the state of Pará (the latter Brazilian state is not marked on Map 3 due to uncertainty of a specific locale, but which was probably Belém (I. Kitching pers. comm.)). There is also one record from northern Venezuela. This species is apparently found in the Brazilian Atlantic Forest, but see remarks below. *Menevia ostia* may range as far north as Belize as per a single report from Pook’s Hill, Belize. This interesting record is treated in more detail in the remarks.

###### Natural history.

*Menevia ostia* is one of the few species of *Menevia* with known host plant associations. Rearing records from D. Janzen & W. Hallwachs show that *Menevia ostia* feeds on both *Terminalia amazonia* and *Terminalia oblonga* (Combretaceae). Additionally, a female specimen from Suriname at the USNM bears a label referencing *Terminalia catappa*, a known host plant of *Menevia plagiata* (Raymundo 1919).

Furthermore, D. Janzen provided us access to photos of reared *Menevia ostia*, published here for the first time (see Figs 4, 5). Larval coloration is quite striking, but shows the usual morphology and one of a number of case structures typical of Mimallonidae.

###### Remarks.

Druce described *Menevia ostia* at a time when most new Mimallonidae that were being described were being placed in the now invalid, preoccupied genus *Perophora* Harris, 1841. Later, Schaus (1928) moved *Perophora ostia* to the genus *Pamea* Walker, 1855, apparently based on wing venation, which along with other wing characteristics used by Schaus, are unreliable in assigning species to genera (St Laurent and Dombroskie 2015). Schaus made no mention of the striking similarity in appearance of *Pamea ostia* to species placed in the genus *Menevia*.

Schaus’s assignment of *Menevia ostia* (and *Menevia parostia* comb. n.) to the genus *Pamea* is strange considering the resemblance of these two species to *Menevia lantona*, a species that he described and designated as type species of *Menevia*, a genus that he also described. In comparing the genitalia of *Menevia ostia* with the type species of *Pamea*, *Pamea albistriga* Walker, 1855, the first author found the male genitalia of *Menevia ostia* completely unlike those of *Pamea albistriga*. The male genitalia of *Pamea albistriga* are very simple in comparison with those of *Menevia ostia* and *Menevia* as a whole. The phallus of *Pamea albistriga* is simple and truncated apically, and lacking a fused juxta with extended superior processes as in *Menevia ostia* and all other *Menevia*. In *Pamea albistriga* the vinculum is much more elongated, valves extremely simple, and the gnathos are atrophied. Genitalia alone offer enough support for the reassignment of *Pamea ostia* to *Menevia* based on the presence of all generic autapomorphies. The external characters of *Menevia ostia* are also sufficient for assigning this species to *Menevia*, namely the postmedial lunule and apical dash, both of which are readily apparent in *Menevia ostia*.

Of the four *Menevia* species-groups, the *ostia* species-group is the most difficult group to tease apart into different morphologically separable species. Phallic structure is an important character for species identification in *Menevia*, but each specimen of *Menevia ostia* examined (n = 14) had a uniquely shaped dorsal phallic ridge, varying in structure from a singular rounded or rectangular hump, to a crest that follows the length of the phallus (compare Figs 79c, 80, and 81). There is some degree of geographic association with the phallus structure. For example, specimens from Costa Rica generally have a more hump-like phallic ridge whereas those from northern South America have more elongated crests along the length of the phallus. There is also a great deal of variation within a single given locality. Specimens from the state of Espírito Santo, Brazil, for example, seem to have dorsal phallic ridges almost intermediate between those found in Costa Rica and northern South America. Furthermore, specimens from the Atlantic Forest biome are paler than those from the rest of the distribution. However, a small number of specimens from Rio de Janeiro contradicted this apparent trend as they were darker and much more similar to Costa Rican populations.

This issue of blurred species boundaries is non-existent in the *lantona* and *lucara* species-groups. Both of these species-groups each have one species endemic to the Brazilian Atlantic Forests, and these endemic species each have unique genitalia and display minor but consistent external differences. The high degree of genitalia and external variation in *Menevia ostia*, even from a single location, impedes our ability to locate species-specific traits. Molecular evidence may be able to offer more conclusive insights, but even then, material is greatly lacking for the *ostia* species-group. Until more data is made available, it is more parsimonious to consider *Menevia ostia* to be a wide-ranging, phenotypically homogeneous species with variable male genitalia.

*Menevia ostia*, the very similar *Menevia parostia* comb. n., and *Menevia pallida*, are apparently distinct on the basis of female morphology and environment, with the latter species being restricted to drier regions of central South America. Only one record of *Menevia ostia* exists from a dry region, a specimen from northern Venezuela. The genitalia of this specimen are surprisingly very similar to those of *Menevia pallida*. A similar issue exists for the specimens from Espírito Santo. Although these specimens are consistently larger and paler than Costa Rican specimens, some specimens from Costa Rica are in fact, larger and paler than most others from similar localities.

Further complicating our understanding of the distributional boundaries of true *Menevia ostia* is a male specimen resembling *Menevia ostia* reported from Pook’s Hill, Belize (M. J. C. Barnes pers. comm.; color photo examined). This record is much farther north in Central America than any other records of the *ostia* group. Only one other species of *Menevia* is known from Belize: *Menevia menapia*. Unfortunately, this specimen is inaccessible to us and it cannot be dissected to determine if it is conspecific with *Menevia ostia*, or if it perhaps represents an undescribed species. Regardless of the specific identity of this specimen, the *ostia* species-group ranges at least as far north as Belize, perhaps making the extreme allopatry of *Menevia menapia* less of a biogeographic oddity relative to the genus *Menevia* as a whole.

##### Menevia parostia

Taxon classificationAnimaliaLepidopteraMimallonidae

(Schaus, 1928) comb. n.

Figs 38 , 96

Pamea parostia Schaus, 1928: 667
Pamea perostia ; Becker 1996, misspelling

###### Type material.

**Holotype**, ♀: **UNKNOWN**: Type No. 33592 U.S.N.M./ *Pamea parostia* type Schaus/ USNM-Mimal: 1107/ St. Laurent diss.: 4-20-15:7/ (USNM) [examined]. Type locality: Unknown.

###### Diagnosis.

*Menevia parostia* can be differentiated from *Menevia ostia* by the placement of the postmedial line of the hindwing, which is roughly midway along the length of the hindwing in *Menevia ostia* and closer to the wing margin in *Menevia parostia*. Furthermore, the sclerotized bands on the venter of the VIII abdominal segment are very thin in *Menevia parostia*. Lack of material and variability of this structure, however, belies its diagnostic capability. Additionally, most (93%, n = 15) female specimens of *Menevia ostia* are much larger than those of *Menevia parostia*. This holotype of *Menevia parostia* does not differ remarkably from the single definitive female of *Menevia pallida* (see remarks).

###### Description.

**Male.** Unknown. **Female.** *Head*: Straw colored, eyes bordered posteriorly by dark brown collar of scales reaching labial palpi, labial palpi moderately long, reaching beyond frons, segments somewhat well defined ventrally, dorsally with darker scales contrasting with overall lighter coloration. Scape and pedicel weakly tufted. *Thorax*: As for genus. Straw colored. *Legs*: As for genus. Tibial spurs relatively thick, long, almost completely scaled except ventrally. *Forewing dorsum*: Forewing length: 18 mm, n = 1. Subtriangular, rounded, apical quarter of outer margins weakly concave, apex slightly falcate. Ground color pale tan-yellow, moderately speckled by dark petiolate scales. Discal spot faintly marked by gray. Apex marked by black scales near tip of apical dash. Postmedial line brown, mostly straight. Submarginal area pale gray, postmedial lunule originating from near where apical dash meets postmedial line, lunule follows postmedial line from apex to one quarter length of postmedial line where lunule smoothly curves outward toward wing margin becoming somewhat diffuse, forming acute angle with postmedial line. Faint white accessory mark present near tornus. Antemedial line very faint, brown, undulating. *Forewing venter*: As in forewing dorsum but more heavily speckled, postmedial line bent outwards near tornus, antemedial line absent, discal spot present, small, black. *Hindwing dorsum*: Rounded, similar coloration and patterning as forewings, postmedial lunule very vague, wavy, not zigzagged, originating near anterior wing margin, following curvature of wing margin, not steeply swept to margin, antemedial area lighter, postmedial line weakly curved, closer to wing margin than midway along wing length. *Hindwing venter*: Following similar pattern as forewing venter but discal mark absent, marginal area color as surrounding area. *Abdomen*: As for genus but stouter. Coloration a continuation of thoracic color. Midventral stripe absent. Sternite of VIII as pair of thin sclerotized bands not touching near anterior margin, bowed out slightly mesally. *Genitalia*: (Fig. 96) n = 1. VIII prominently sclerotized laterally, appendicular apophyses present. Tergite of VIII arch-like, converging mesally to form posteriorly directed point. Apophyses anteriores slightly shorter than apophyses posteriores. Lamella antevaginalis as semicircular, sclerotized band. Ductus bursae short. Papillae anales rectangular when viewed ventrally, covered in short setae.

###### Distribution.

Unfortunately the holotype is without locality information, furthermore, Schaus’s original (1928) description listed the “habitat” as “unknown.”

###### Remarks.

Schaus (1928) described *Pamea parostia*, known only from the female holotype, based on its resemblance to *Menevia ostia*, differing only by its smaller size, “reduced” markings, and the “more developed” frenulum. Upon examining the holotype and comparing it with the much larger female holotype of *Menevia ostia* and a series of mostly larger females from Costa Rica, we found that Schaus was incorrect in his assertion that the frenulum of *Menevia parostia* is more developed, when in fact the frenulum appears to be of the same size and arrangement in examined *Menevia ostia* females. Schaus frequently failed to locate the frenulum despite its presence, as shown by previous authors (Pearson 1951, 1984, St Laurent and Dombroskie 2015). Furthermore, the size difference between *Menevia parostia* and *Menevia ostia* is certainly not enough evidence to maintain *Menevia parostia* as a valid species. Among the *Menevia ostia* specimens from Costa Rica that were examined in this work, one female specimen from Tomatera was actually smaller than the holotype of *Menevia parostia*, along with a similarly very small male. Public barcode data shows this smaller pair of *Menevia ostia* display no differences whatsoever from regularly sized *Menevia ostia* from nearby locations (BOLD). Many species of Mimallonidae frequently display dwarfed specimens (R. A. St. Laurent pers. obs.), perhaps due to poor host plant assimilation or other environmental factors, potentially explaining the small size of the single pair of *Menevia ostia*.

The presence of such small specimens of *Menevia ostia* originally lead us to believe that *Menevia parostia* must be just another small example of this species, well within the natural size range and we were prepared to synonymize *Menevia parostia* with *Menevia ostia*. However, additional examination of the holotype of *Menevia parostia* revealed characters of the hindwing maculation and genitalia that were not seen in any examined *Menevia ostia*. The paired sternites of VIII in *Menevia parostia* are slightly thinner overall in comparison to those of *Menevia ostia*, but this character is rather variable in general. A more dramatic difference is found in the arrangement of the hindwing postmedial line between the two species (see Figs 36, 37 compared with 38). The holotype of *Menevia parostia* is strikingly similar to a female specimen of *Menevia pallida* in relative size, coloration, and arrangement of the hindwing postmedial line. Unfortunately, the abdomen of the female *Menevia pallida* is missing and thus a genitalia comparison cannot be performed. It is quite possible that *Menevia pallida* is a junior synonym of *Menevia parostia*, but without locality data, the abdomen of the female *Menevia pallida*, or true males of *Menevia parostia*, we cannot render this conclusion definitive. Pending further data, we therefore retain both *Menevia parostia* and *Menevia pallida* as valid species.

**Figures 30–41.**
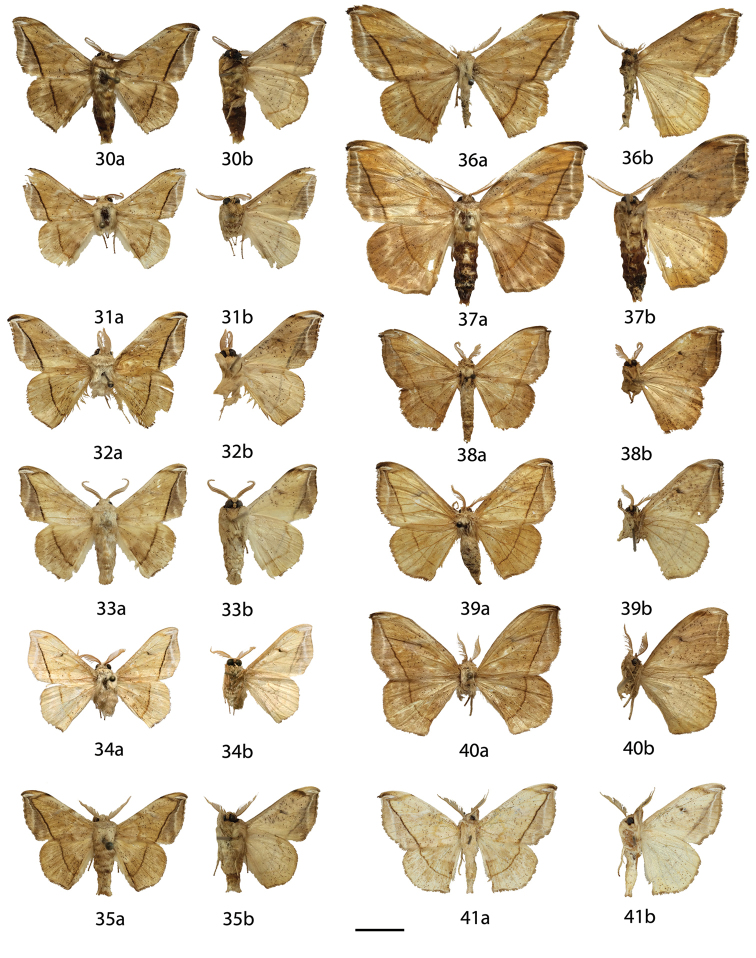
*Menevia ostia* species-group adults, **a** recto, **b** verso. **30** *Menevia ostia* ♂, Costa Rica, Guanacaste, Tajo Angeles, 540 m, 10-SRNP-4309 (USNM) **31** *Menevia ostia* ♂, Costa Rica, Guanacaste, Sendero Puertas, 400 m, 03-SRNP-18623 (USNM) **32** *Menevia ostia* ♂, French Guiana, Sinnamary, Piste a St. Elie, km. 24, 50 m (AMNH) **33** *Menevia ostia* ♂, Brazil, Espírito Santo, Linhares, 40 m (USNM) **34** *Menevia pallida* holotype ♂, Brazil, Maranhão, Feira Nova do Maranhão, 480 m (DZUP) **35** *Menevia pallida* paratype ♂, Brazil, Maranhão, Feira Nova do Maranhão, 480 m (CGCM) **36** *Menevia ostia* holotype ♀, Panama, Chiriquí (MNHU) **37** *Menevia ostia* ♀, Costa Rica, Guanacaste, Sendero Rotulo, 510 m, 04-SRNP-34126 (USNM) **38** *Menevia parostia* holotype ♀, unknown locality (USNM) **39** *Menevia pallida* ♀ [questionable], Brazil, Minas Gerais, Lassance (CUIC) **40** *Menevia pallida* ♀, Brazil, Maranhão, Caixas, Reserva Ecol. Inhamum (CPAC) **41** *Menevia pallida* ♀ [questionable], Paraguay, Villarrica [photo couresty NHMUK] (NHMUK). Scale bar = 1 cm.

##### Menevia pallida

Taxon classificationAnimaliaLepidopteraMimallonidae

Herbin & Mielke, 2014

Figs 34 , 35 , 39 , 40 , 41 , 82 , 97 ; Map 3

Menevia pallida Herbin & Mielke, 2014: 147–149; figs ♂ 52–54, ♂ genitalia 55, 56

###### Type material.

**Holotype**, ♂: **BRAZIL: Maranhão**: genitalia prep. D. Herbin, ref H. 986 [blue label]/ BRESIL Maranhao [Maranhão], Feira Nova do Maranhão, Retiro, 480 m. 21-25.II.2012, -07°00'31"S, -46°26'41"W, C. Mielke leg. Coll. D. Herbin / DZ 15.734/ Holotype ♂, *Menevia pallida* Herbin & Mielke det., Antenor 2014 [red label]/ (DZUP) [examined]. Type locality: Brazil: Maranhão: Feira Nova do Maranhão.

**Paratype**, 1 ♂, **BRAZIL: Maranhão**: Feira Nova do Maranhão, Retiro, 46°26'41"W, -07°00'31"S, 480 m: 16–17.II.2013, C. Mielke leg., Paratypus *Menevia pallida* Herbin & C. Mielke det., 2014 [green label], Col. C. Mielke 26.349, St. Laurent diss.: 6-16-15:3 (CGCM).

###### Additional specimen examined.

**BRAZIL: Maranhão**: 1 ♀, Caixas, Reserva Ecol. Inhamum, 13°12'S, 45°27'W [given coordinates inaccurate], 110 m: 27.II.2006–1.III.2006, lençol e luz mista [sheet and mixed light], F. Limeira-de-Oliveira & J.C. Silva cols, Coleção Embrapa-CPAC No. 20053, [missing abdomen, no genitalia prep.] (CPAC).

###### Additional questionable specimens examined.

(3 ♀ total) **BRAZIL: Minas Gerais**: 1 ♀, Lassance: 12.XI.1919, Cornel Univ. Expedition Lot 569, Sub 116, Cornell U. Lot 672, Sub 385 Det. W. Schaus [*ostia* ♀], St. Laurent diss.: 4-20-15:6 (CUIC). **PARAGUAY**: 1 ♀, Amambay, Parque Nacional Cerro Corá, 22°39'S, 56°01'W: 7–10.IV.1986, M. Pogue & M. Solis, St. Laurent diss.: 5-8-15:1 (USNM). 1 ♀, Guairá, Villarrica: 21.IX.1926, F. Schade, ex. Joicey coll. 1925–157, BMNH(E) 1377142 (NHMUK).

###### Diagnosis.

*Menevia pallida* can be differentiated from the somewhat larger, but very similar *Menevia ostia* by the pale tan to yellowish brown ground color as opposed to gold or pale yellow. Additionally, the dark speckling is usually heavier due to the presence of more petiolate scales. The hindwings are without bright, obvious, zigzagged postmedial lunules as in *Menevia ostia*. In both *Menevia pallida* and *Menevia parostia*, the placement of the postmedial line on the hindwing is closer to the wing margin than to midway along the length of the wing as it is in *Menevia ostia*. The phallic ridge is more smoothly curved and less quadrate terminally than in *Menevia ostia*, with the front edge of the phallic ridge evenly sloped rather than squared. The female of *Menevia pallida* is smaller than those of *Menevia ostia*. We are unable to provide characters to differentiate the unique female of *Menevia parostia* from female *Menevia pallida*, although the females of *Menevia pallida* at our disposal (both true and questionable specimens) are always slightly larger than the unique specimen of *Menevia parostia*.

###### Description.

**Male.** *Head*: Pale tan, eyes bordered posteriorly by dark brown collar of scales reaching labial palpi, labial palpi small, segments somewhat well defined ventrally, dorsally with darker scales contrasting with overall lighter coloration. Scape and pedicel weakly tufted. *Thorax*: As for genus. Pale gold-tan. *Legs*: As for genus. Tibial spurs relatively thick. *Forewing dorsum*: Forewing length: 15.5–17 mm, avg.: 16.3 mm, n = 2. Triangular, apical half of outer margins weakly concave, apex slightly falcate. Ground color pale tan to yellowish, moderately speckled by dark petiolate scales. Discal spot faintly marked by gray. Apex marked by black scales above apical dash. Black postmedial line mostly straight, sometimes weakly undulating or kinked. Submarginal area pale gray, postmedial lunule originating from near where apical dash meets postmedial line, lunule follows postmedial line from apex to one quarter length of postmedial line where lunule smoothly curves outward toward wing margin becoming somewhat diffuse, forming acute angle with postmedial line. White accessory mark present near tornus. Antemedial line faint, brown, undulating. *Forewing venter*: As in forewing dorsum but two postmedial lines present, both much fainter than single line on dorsum, one line convex near tornus and slightly undulating, the other straight, following the postmedial line of wing dorsum, antemedial line absent, small black elongated discal spot present. *Hindwing dorsum*: Rounded, anal angle weakly accentuated, similar coloration and patterning as forewings, postmedial lunule very vague, wavy, not zigzagged, originating near anterior wing margin, following curvature of wing margin, not steeply swept to margin, antemedial area lighter, postmedial line straight, weakly undulating near anal angle. *Hindwing venter*: Following similar pattern as forewing venter but discal mark absent, marginal area color as surrounding area. *Abdomen*: As for genus. Coloration a continuation of thoracic color. Midventral stripe absent. *Genitalia*: (Fig. 82) n = 2. Tegumen subtriangular to nearly ovoid. Vinculum narrow, somewhat accentuated quadrate corners. Valves slightly asymmetrical, short, saccular edge of left valve with large triangular tooth proximal to transtilla, right valve with tooth much reduced in size, both valves with smaller central ridge immediately above saccular edge teeth. Valves somewhat indented mesally, rounded apically. Uncus triangular, apex sharp, moderately hooked. Gnathos as two, flattened, spade-shaped outward facing flaps, bent inward, tips nearly meeting. Juxtal processes shorter than phallus, narrow, flattened, slightly curved, smooth. Base of phallus with paired, rounded, diverging, backwards facing fingerlike lobes. Phallus curved, broad, lengthwise dorsal ridge present. Left edge of phallus forms distinct setae covered ridge, extended along phallus length, smoothly sloped at anterior terminus, distal tip of phallus weakly downturned separated into two distinct, bent points. Vesica bag-like. **Female.** *Head*: As in male but scales paler, labial palpi longer, thinner, dorsally covered in less dark scales. *Thorax*: As in male. *Legs*: As in male. *Forewing dorsum*: Forewing length: 21 mm, n = 1. Coloration and patterning exactly as in male, wing shape broader. *Forewing venter*: As in forewing dorsum but lighter, markings subdued, postmedial line much fainter, weakly bent near tornus; antemedial line not present; faint, elongated black discal spot present, darkest mesally. *Hindwing dorsum*: As in male but slightly broader, larger, submarginal area grayish. *Hindwing venter*: Following similar pattern as forewing venter except lighter with very little maculation. [Abdomen and genitalia based on a questionable specimen from Minas Gerais that may not be attributable to *Menevia pallida*, the single Maranhão female is missing an abdomen and thus genitalia are unavailable]. *Abdomen*: As in male, but stouter. Sternite of VIII as pair of elongated sclerotized bands, bowed out midway along length. *Genitalia*: (Fig. 97) n = 1. VIII prominently sclerotized laterally, appendicular apophyses absent. Tergite of VIII arch-like, converging mesally to form posteriorly directed point. Apophyses anteriores shorter than apophyses posteriores. Lamella antevaginalis as semicircular, sclerotized band. Ductus bursae short. Papillae anales subtriangular when viewed ventrally, covered in setae.

###### Distribution

(Map 3). *Menevia pallida* is potentially restricted to drier Cerrado habitat. Reliable records exist only from the Brazilian state of Maranhão. We questionably consider a female specimen from eastern Minas Gerais, Brazil to be this species due to the presence of Cerrado at that locality (IBGE 2004). Additional records from Paraguay are herein provisionally considered *Menevia pallida*, but likely represent an additional undescribed species, see remarks below.

###### Remarks.

The recently described *Menevia pallida* is not very distinct from the widespread *Menevia ostia* or from the unique specimen of *Menevia parostia*. The differences between *Menevia pallida* and *Menevia ostia*, while present, provide only a weak basis on which to consider these taxa as separate species. The original description of *Menevia pallida* was based on comparisons with *Menevia lantona*, which was considered the most similar species by Herbin and Mielke (2014). *Menevia ostia* was not mentioned by these authors, despite the almost exact same external and genitalia morphologies of the males of the two species. In addition, the authors reported that the genitalia of *Menevia pallida* are “the same” as *Menevia lantona*, which we have found not to be the case. The genitalia of the holotype of *Menevia pallida* in Herbin and Mielke (2014) displays very little resemblance to any *Menevia lantona* dissections that we have reviewed (compare Figs 72 and 82). Most notably, the uncus of *Menevia lantona* is handbell shaped rather than triangular, the valves narrower, the gnathos processes more flattened and triangular, and finally the phallus of *Menevia lantona* is of an entirely different shape. The phallus of *Menevia lantona* lacks a dorsal ridge, which is a prominent character of the entire *ostia* species-group. Apparently the phallus of *Menevia pallida* was not fully examined during the description, as it was not figured separately from the rest of the genitalia nor was it removed from the genitalia when one of us examined the genitalia preparation.

Despite the issues with the original description of *Menevia pallida*, we still consider it a valid species based primarily on environmental differences, consistently smaller size, paler tan rather than golden coloration, the position of the hindwing postmedial line in females, and the potential difference in female genitalia compared to female *Menevia ostia*. The female from Caixas, Maranhão, Brazil, was collected about 440 km northeast of the type locality of *Menevia pallida*, and from the same Cerrado habitat (Silva et al. 2012), and thus is the single female specimen most likely associated with *Menevia pallida*. We consider this association reliable because the size and maculation agree perfectly with the examined holotype and paratype males of *Menevia pallida*.

The single female from Lassance, Minas Gerais, Brazil, which we associate here with *Menevia pallida*, albeit questionably, is relatively small and pale compared to *Menevia ostia* females from Central America and northern South America, and consequently seems more in line with *Menevia pallida*. However, as explained in the remarks to *Menevia ostia*, some populations from Atlantic Forest localities are similarly pale. Pending upon the availability of additional specimens of both sexes from Minas Gerais and Espírito Santo, we are unable to conclusively allocate these populations to either species.

We consider two females from Paraguay to be *Menevia pallida* due to their localities near the Cerrado, small size, and pale coloration. However, the forewing shape is less rounded than in female *Menevia ostia* and *Menevia pallida*, making the Paraguayan specimens appear rather distinct (see Fig. 41). Because of this morphological difference, these specimens were not included when writing the female description for *Menevia pallida*. Due to the fact that we lack males from Paraguay, we are unable to determine conclusively whether the specimens from Paraguay represent *Menevia pallida* or a distinct, undescribed species. Regardless, the specimens from Paraguay certainly belong to the *ostia* species-group and are treated here in the present study as they represent the only records of *Menevia* from Paraguay and are therefore significant.

#### *plagiata* species-group

The *plagiata* species-group contains the largest *Menevia*, the females of some species are among the largest Mimallonidae. Sexual dimorphism is very pronounced in this group. Forewings of females are longer and broader than those of the males, which are conversely very falcate. Unlike in the three previous species-groups, the species belonging to the *plagiata* group do not have the curved postmedial lunules, but instead have distinct white bands that border the outer margin of the postmedial line, either along the complete length of the line as in the *vulgaris* subgroup containing: *Menevia vulgaris* sp. n., *Menevia franclemonti* sp. n., *Menevia vulgaricula* sp. n., *Menevia cordillera* sp. n., and *Menevia delphinus* sp. n.; or are interrupted midway along the line as in the *plagiata* subgroup, which contains: *Menevia plagiata*, *Menevia australis* sp. n., and *Menevia alurca*. The lack of the lunules immediately distinguishes this species-group from the others. However, the presence of the apical dash is very distinctive and thus the species in this group are easily identifiable as *Menevia*. Ground color is also darker for this group, most similar to the *lucara* group, being primarily gray, brown, or some combination. The male genitalia are more robust than in other species-groups. The paired gnathos is flat and oblong, either somewhat triangular or partly ovoid, usually with extreme truncation distally. The phallus is diverse in form, ranging from elongated and smooth to almost triangular due to an exaggerated dorsal projection when viewed laterally. The juxtal arms are generally very flat and wide, not sharp apically.

##### Key to *plagiata* species-group

**Table:** 

1	Forewing with continuous white band along exterior edge of postmedial line, midventral abdominal stripe absent	**4 [*vulgaris* subgroup**]
–	Forewing with interrupted white band along exterior edge of postmedial line, midventral abdominal stripe present	**2 [*plagiata* subgroup**]
2	Postmedial line usually straight, brown with little red coloration	**3**
–	Postmedial line usually bent outwards mesally, coloration dark gray with predominance of red suffusion. Central South America, especially in the Brazilian Cerrado	***Menevia alurca***
3	Wings relatively narrow, usually grayish, white band of forewing postmedial line usually reaching apical tip of wing, especially prominent in females, phallus with elongated, thin, dorsal projection. Vicinity of the state of Rio de Janeiro	***Menevia plagiata***
–	Wings broader, usually brown, white band of forewing postmedial line usually intercepting apical dash mesally, or dissipating before reaching apex, especially prominent in females, phallus with triangular or rounded dorsal hump. Southeastern Brazil, especially Santa Catarina north to São Paulo	***Menevia australis* sp. n.**
4	Large, forewing length males: 22–28 mm, females: 27.5–39 mm, phallus smoothly curving, dorsally smooth or with irregular edge, but never with distinct bulge	**5**
–	Small, forewing length males: 17–23 mm, females: 27 mm, phallus with dorsal bulge anteriorly or central protuberance	**6**
5	Phallus dorsum smooth, evenly edged, terminal sclerotized edge that becomes vesica, diagonal; ventral bump of phallus angled away from phallus terminus. Southeastern Brazil	***Menevia franclemonti* sp. n.**
–	Phallus dorsum with irregular edge, terminal sclerotized edge that becomes vesica nearly vertical, ventral bump of phallus absent or indistinct. Northern South America	***Menevia vulgaris* sp. n.**
6	Lobes at base of phallus rounded, not peg-like. Andean or Brazilian Cerrado	**7**
–	Lobes at base of phallus peg-like. Amazonian	***Menevia vulgaricula* sp. n.**
7	Forewings elongate, very acute apically, forewing postmedial line mostly straight only slightly curved. Andean Cordillera Oriental, Peru to Bolivia	***Menevia cordillera* sp. n.**
–	Forewings stouter, less acute apically, forewing postmedial line slightly undulated. Endemic to the Brazilian Cerrado	***Menevia delphinus* sp. n.**

##### *plagiata* subgroup

The *plagiata* subgroup contains *Menevia plagiata*, *Menevia australis* sp. n., and *Menevia alurca* and is diagnosed by the discontinuous white band along the outer margin of the postmedial line and by the presence of a midventral abdominal stripe. The species boundaries in the *plagiata* subgroup are unclear and thus we consider the three species in this subgroup as part of a species complex hereby-considered the “*Menevia plagiata* species complex” and may contain additional species not formally recognized herein.

###### Menevia plagiata

Taxon classificationAnimaliaLepidopteraMimallonidae

(Walker, 1855)

Figs 42–44 , 52 , 53 , 83 , 84 , 98 ; Map 4

Mimallo plagiata Walker, 1855: 1341
Perophora plagiata ; Raymundo 1919
Menevia plagiata ; Schaus 1928: fig. ♀ 88g
Perophora plagiata ; Monte 1934
Menevia plagiata ; Lima 1950
Menevia plagiata ; Silva et al. 1968
Menevia plagiata ; Becker 1996
Menevia plagiata ; Mecke et al. 2000
Menevia plagiata ; Pastrana 2004
Menevia plagiata ; Herbin and Mielke 2014

####### Type material.

**Holotype**, ♂, presumed lost/destroyed. Type locality: Brazil: Rio de Janeiro (see remarks).

**Neotype (here designated)**, ♂: **BRAZIL: Rio de Janeiro**: BRAZIL: Rio de Janeiro Ste., Teresopolis [Teresópolis], 13–22.iii.1958, H.B.D. Kettlewell, B.M. 1958–273/ NEOTYPE male *Mimallo plagiata* designated by St Laurent and Dombroskie 2016/ BMNH(E) 1378747/ St. Laurent diss.: 9-2-15:1/ (NHMUK). New type locality: Brazil: Rio de Janeiro: Teresópolis.

####### Additional specimens examined.

(10 ♂, 19 ♀ total) **BRAZIL: Espírito Santo**: 3 ♀, Santa Teresa: 20.XI.1966, 18.XII.1966, Elias leg. (DZUP); XII.1966, C. & C.T. Elias (DZUP). **Pernambuco**: 1 ♂, Serra de Communaty [southeastern Pernambuco]: 1.II.1893, E. Gounelle, Ex. Oberthür Coll., Brit. Mus. 1927–3, ex. Joicey Coll. Brit. Mus. 1925–157, BMNH(E) 1378748, St. Laurent diss.: 6-29-15:12 (NHMUK). 1 ♀, Canhotinho: 19.VII.1991, Cardoso leg., ex. coleção A. Cardoso, No. 5467 (DZUP). **Rio de Janeiro**: 3 ♂, Teresópolis: 13–22.III.1958, H.B.D. Kettlewell, B.M. 1958–273, BMNH(E) 1378746, 1378751, 1378752, St. Laurent diss.: 6-29-15:13, 6-29-15:14 (NHMUK). 3 ♂, 4 ♀, Petrópolis: 2.XII.1875, 10.XII.1875, 29.III.1876, Joicey Bequest, Brit. Mus. 1934–120, BMNH(E) 1378749, St. Laurent diss.: 6-29-15:15 (NHMUK); 10.IV.1928, 6.IV.1959, 26.IV.1960, 23.X.1964, Gagarin leg., ex. col. Gagarin (DZUP). 1 ♂, Petrópolis, Independência: 21.IX.1939, Gagarin leg., ex. col. Gagarin (DZUP). 1 ♀, Mendes, 92 km from Rio de Janeiro: Collection Le Moult, Joicey Coll. Brit. Mus. 1925–157 (NHMUK). 2 ♀, Corcovado, 800 ft: II.1910, 2.II.1910, E.D. Jones, E.D. Jones coll. 1919–295, [1 ♀ is the “type” of manuscript name *Perophora* ‡*superba* D. Jones, BMNH(E) 805425] (NHMUK). 2 ♀, Eugenho de Dentro, E.F.C.B.: 1.I.1947, 4.I.1947, ex. pupa, Nelson Almeida, No. 19.174, 19.175 ex. coll. D’Almeida (DZUP). 1 ♀, Três Rios, Jacarepagua [additional illegible data]: 17.IX.1929, Ferr. d’Almeida, ex. coll D’Almeida, No. 19.173 (DZUP). 1 ♀, “Penedo, Rezende” [either Penedo or Resende]: 4.X.1955, Coleção Richard Frey (DZUP). 2 ♂, 3 ♀, no additional locality data: Collection Wm Schaus, USNM-Mimal: 1202, St. Laurent diss.: 6-19-15:6 (USNM); 10.IX.1912, M.J. Holland, Carn. Mus. Acc. 4770, St. Laurent diss.: 4-25-15:2 (CMNH); Ex. Coll. J. Doll, Ac. 24352, St. Laurent diss.: 7-28-15:1 (AMNH); 12.IX.1943, Gagarin leg. ex. col. Gagarin (DZUP); IX.1944, ex. coleção A. Cardoso (DZUP). 1 ♀, additional locality information illegible: 5.I(?).1930, Ferreira d’Almeida, ex. coll. d’Almeida (DZUP).

####### Diagnosis.

*Menevia plagiata* is recognizable from all previous species in both sexes by the replacement of the wing margin-swept postmedial lunule with a white band along the length of the postmedial line, which is interrupted midway and resumes near the inner margin. The female of *Menevia plagiata* is differentiated from the following two similar species by the presence of a straight or only weakly undulated postmedial line, which, along with the white accessory band, curves toward the apex of the forewing, sometimes sharply, usually to the wingtip, rather than ending at or before reaching the apical dash. Male genitalia are unlike any other species except *Menevia alurca*, in that the phallus bears a prominent, elongated, pointed projection from the dorsal surface and is not smooth or ridged as in all other previously diagnosed species. The nearly straight forewing postmedial lines (except near the apex) distinguish both male and female *Menevia plagiata* from *Menevia alurca*, whereas male genitalia characters and the apical curve of the female forewing postmedial line distinguish *Menevia plagiata* from *Menevia australis* sp. n.

####### Description.

**Male.** *Head*: Gray, eyes large comprising about two-thirds of head area, eyes bordered posteriorly by darker gray collar of scales reaching labial palpi, labial palpi very small, dorsally with darker scales contrasting with overall gray coloration. Scape and pedicel tufted. *Thorax*: As for genus. Light gray-brown. *Legs*: As for genus. Tibial spurs small to moderate in length, almost entirely scaled. *Forewing dorsum*: Forewing length: 22–24.5 mm, avg.: 22.4 mm, n = 7. Triangular, apical half of outer margins concave, apex falcate. Ground color gray-brown with darker gray, brown suffusion especially near interior edge of postmedial line and medial area, reddish coloration near apex along apical interior of postmedial line, overall lightly speckled by dark petiolate scales. Discal spot faintly marked by light gray oblong shape, thin gray mark connecting discal spot to costa. Apex marked by black scales above apical dash, especially near apical tip. Postmedial line straight or weakly undulated, line black, strongly contrasting. Submarginal area light gray with whitish suffusion mesally, postmedial lunule as white band originating from apical dash, white band follows postmedial line from apex to midway along postmedial line, resuming near anal margin. Antemedial line faint, brown, curved outwards. *Forewing venter*: As in forewing dorsum but grayer rather than brownish, sometimes with pinkish hue, black portion of postmedial line mostly absent except medially where very dark, white outer band of postmedial line as in dorsum, antemedial line absent. *Hindwing dorsum*: Subtriangular, anal angle weakly accentuated, reddish suffusion near anal angle, similar coloration and patterning as forewings, except postmedial lunule present as zigzagged mark, originating from white outer band along first quarter of postmedial line, postmedial line usually sharply bent toward anterior wing margin, sometimes weakly concave mesally. *Hindwing venter*: Following similar pattern as forewing venter, but red coloration near anal angle and medial area much darker. *Abdomen*: As for genus but elongated, nearly sphingiform, reaching beyond anal margin of hindwing. Coloration a continuation of gray thoracic color. Dark, contrasting, midventral stripe present along entire length. *Genitalia*: (Figs 83, 84) n = 7. Somewhat variable; tegumen ovoid or rounded rectangular, sometimes weakly constricted near base of gnathos. Vinculum narrow, somewhat quadrate ventrally. Valves short, stocky, bent outwards or more elongated; saccular edge of left valve with large triangular tooth proximal to transtilla, sometimes notched mesally, right valve with tooth slightly reduced in size, both valves with prominent mesal costal projection originating from central ridge of valve, projection immediately above saccular edge teeth, apex of mesal projection pointed toward saccular edge. Valves triangular, rounded, or somewhat pointed apically. Uncus truncated apically, apex rounded. Gnathos as two prominent flattened, moderately sclerotized, flap-like, somewhat triangular, outward-facing extensions with truncated apices. Apices usually form fingerlike projections of varying length. Juxtal processes roughly phallus length, moderately sclerotized, curving toward apex of phallus. Juxtal processes very thin, long, widening distally, covered in fine setae, especially apically. Base of phallus with paired, backwards facing, elongated, rounded, diverging lobes sometimes with pointed tips on one or both lobes. Phallus irregularly shaped, unevenly edged dorsum lacking an extensive dorsal ridge but with prominent, elongated or sharply triangular, pointed protuberance, covered in setae, apical tip usually bent backwards. Left edge of rolled phallus uneven, forming extended protuberance, right edge usually with setae covered bulge laterally; base of sclerotized terminus of phallus with prominent ventral bump, angled away from distal end of phallus. Distal tip of phallus separated into two distinct points of varying length. Vesica elongated. **Female.** *Head*: As in male. *Thorax*: As in male. *Legs*: As in male. *Forewing dorsum*: Forewing length: 27.5 mm, n = 1. Maculation as in male, wing broader, more ovoid, less triangular, postmedial line may be bent slightly outward mesally, outer white band of postmedial line curved toward apex, continuously to wingtip, forming very acute angle at junction with apical dash, dark scaling above apical dash usually concentrated near apical tip. *Forewing venter*: As in forewing dorsum but grayer. *Hindwing dorsum*: As in male but more rounded, less triangular, postmedial line straight except for sharp turn towards anterior wing margin, not concave mesally. *Hindwing venter*: Following similar pattern as forewing venter, reddish-brown suffusion near anal angle much darker. *Abdomen*: As in male but more robust. Sternite of VIII as pair of elongated sclerotized bands curving toward each other near anterior edge of VIII segment, but never converge. *Genitalia*: (Fig. 98) n = 1. Tergite of VIII forms triangular, posteriorly directed arc. Apophyses anteriores shorter than apophyses posteriores, apophyses very thin. Lamella antevaginalis thin, C-shaped, weakly notched mesally near ostium bursae. Ductus bursae short. Papillae anales subtriangular, covered in relatively long setae.

####### Distribution

**Map 4.**
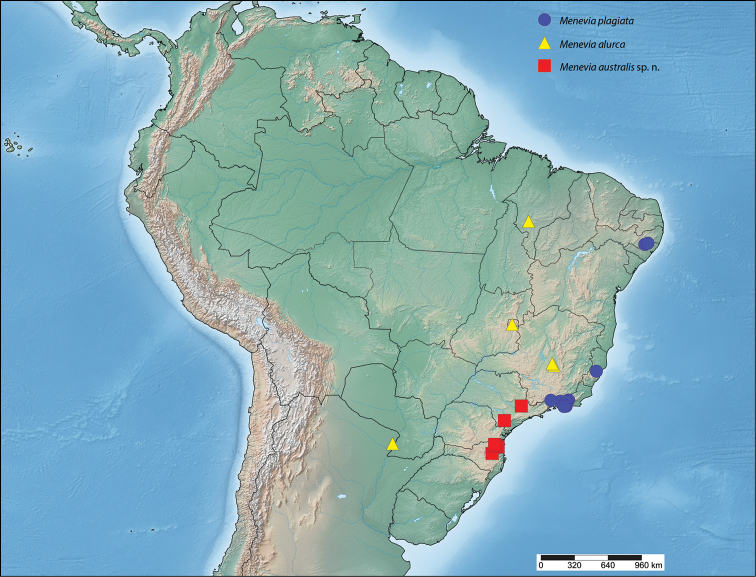
*Menevia plagiata* species-group [*plagiata* subgroup].

(Map 4). *Menevia plagiata* is found in the Brazilian Atlantic Forest. Most records come from the state of Rio de Janeiro, but records also exist from farther north in Espírito Santo and Pernambuco states. The distribution of this species probably extends along the entire coast of Brazil north from the state of Rio de Janeiro.

####### Natural history.

A number of host records exist in the literature for *Menevia plagiata* *sensu lato*, but due to the uncertainty of the identification of this taxon in the past (see remarks below), we cannot be certain that all of the following records pertain to *Menevia plagiata* *sensu stricto*. Perhaps the most reliable record comes from Raymundo (1919) because this author figures the adult of true *Menevia plagiata* and describes its distribution as only including the Brazilian states of Rio de Janeiro and Espírito Santo, which eliminates the more southerly distributed *Menevia australis* sp. n. and the primarily Amazonian *Menevia vulgaris* sp. n., both described below. Raymundo (1919), Monte (1934), and Lima (1950) mention only *Terminalia catappa* (Combretaceae) as a host of *Menevia plagiata*, a species we previously showed to be a host of *Menevia ostia*. Additional, less verifiable host plant records include: *Psidium guajava* (Myrtaceae), *Licania tomentosa* (Chrysobalanaceae), and even *Araucaria angustifolia* (Araucariaceae) all cited in Silva et al. (1968). Silva et al. (1968) refers to “pinheiro” (Pine), which we interpret to mean *Araucaria angustifolia* because this host is listed by Mecke et al. (2000) for *Menevia plagiata*, citing Silva et al. (1968). Additionally, Pastrana (2004) mentions *Prunus amygdalus* (Rosaceae) as a host for *Menevia plagiata*, but this is probably an erroneous misinterpretation of Lima (1950) who had listed “amendoeira (*Terminalia catappa*)” as the host of *Menevia plagiata*, referring to amendoeira da praia (*Terminalia catappa*), not true amendoeira (almond, *Prunus amygdalus*).

####### Remarks.

*Menevia plagiata* is the most problematic taxon in the genus, largely due to the unavailability of the holotype, which is presumed to be lost. The holotype of *Menevia plagiata* originated from Fry’s collection, and was collected in Rio de Janeiro. Becker (2001) provided information pertaining to the history of the many specimens from the Fry Collection, collected in Rio de Janeiro, later described by Walker, that were subsequently damaged, and are apparently now lost. We attempted to locate the holotype of *Menevia plagiata* at the NHMUK but were unsuccessful. Contacting the University Museum, Oxford in an effort to locate the holotype there was also unsuccessful. Although it is not impossible that this type remains undiscovered somewhere in these or other collections, we consider it unlikely to be located, thus we here designate a neotype for this species based on information discussed below.

Walker’s (1855) original description of *Menevia plagiata* is rather vague and could arguably be attributed to either *Menevia plagiata* or *Menevia franclemonti* sp. n. as both of these species are found in Rio de Janeiro and are somewhat similar in appearance. The most important line in Walker’s description is that relating to the white band, which follows the postmedial line in both *Menevia plagiata* and *Menevia franclemonti* sp. n. This band is interrupted in the former and continuous in the latter. Walker (1855) states in his original description of *Menevia plagiata* that there is a “very oblique slender white band at three-fourths of the length, forming a very acute angle near the tip,” which we interpret to mean the white band following the exterior of the postmedial line. However, Walker does not mention whether the white band is continuous or interrupted along the length of the postmedial line, which is necessary information to attribute this description definitively to either species. Walker’s description of *Mimallo saturata* Walker, 1855 from the same work offers some characters that could be attributed to what we consider *Menevia plagiata*, in which the most important again is the white band along what is assumed to be the postmedial line: “a whitish slender slightly oblique band, which has a blackish border on its outer side, and extends from near the tip of the costa to three-fourths of the length of the interior border” (Walker 1855). The holotype of *Menevia saturata* is also presumed lost due to it originating from Fry’s collection and having a type locality of Rio de Janeiro.

An additional taxonomic issue was created by the presence of the “holotype” of *Perophora* ‡*superba* Jones in the NHMUK. The name ‡*superba* is a manuscript name and was never published by Jones. It is possible that Jones realized the similarity of ‡*superba* to *Menevia plagiata* as described by Walker, and hence did not describe it. This assumption is an additional piece of evidence supporting our concept of *Menevia plagiata* because the “holotype” of ‡*superba* matches it. If a validly published description using the name ‡*superba* is located, it would have to be treated as a junior synonym of *Menevia plagiata*.

After visiting the NHMUK and reviewing the specimens belonging to the *Menevia plagiata* species-group, it was clear that all Rio de Janeiro specimens matched our concept of *Menevia plagiata*, including a number of specimens from the late 1800’s. The other species that the name *plagiata* could be associated with, which we describe below as *Menevia franclemonti* sp. n., is rarer relative to *Menevia plagiata* and was not present in the NHMUK. Schaus (in Seitz 1928) illustrated our concept of *Menevia plagiata* as this species, and did not figure anything resembling *Menevia franclemonti* sp. n. Therefore; it seems most probable that Walker had a specimen matching our concept of *Menevia plagiata* at his disposal when writing his description. Finally, one of the oldest *Menevia plagiata* determinations in the NHMUK, from 1889 by C. Berg, was a female specimen matching our concept of *Menevia plagiata*. This provides an indication that as early as 1889 the name *plagiata* was being associated with the species that we consider to be *Menevia plagiata*.

The taxonomic history of the name *plagiata* is surely complicated, and unfortunately, without seeing the holotype, we might never be completely sure that our concept of the species coincides with that of Walker’s (1855). Complications compound further when considering the great deal of variation, both externally and in male genitalia morphology, that *Menevia plagiata* displays within the state of Rio de Janeiro. The considerable amount of variation suggests that *Menevia plagiata* *sensu lato* may represent a species complex. However, the four examined NHMUK specimens from Teresópolis, Rio de Janeiro, including the neotype, represent a cohesive series in terms of external characteristics and genitalia, with very little variation. The valves of these specimens are much stouter and the projection of the phallus shorter than in other *Menevia plagiata* from nearby locations in the state of Rio de Janeiro. Therefore, to stabilize the nomenclature, we here designate the neotype, chosen from this series. A specimen from Petrópolis, Rio de Janeiro at DZUP also greatly resembles the series of four *Menevia plagiata* from Teresópolis (see Fig. 43).

**Figures 42–51.**
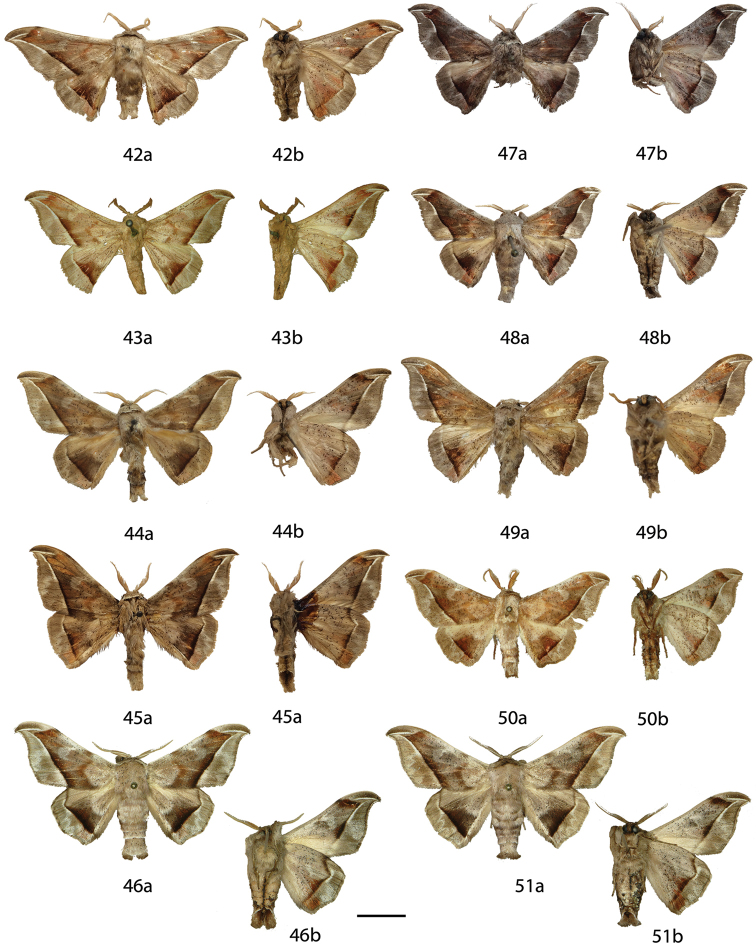
*Menevia plagiata* species-group [*plagiata* subgroup] male adults, **a** recto, **b** verso. **42** *Menevia plagiata* neotype ♂, Brazil, Rio de Janeiro, Teresópolis (NHMUK) **43** *Menevia plagiata* ♂, Brazil, Rio de Janeiro, Petrópolis, Independência, 900 m [photo courtesy CGCM] (DZUP) **44** *Menevia plagiata* ♂, Brazil, Rio de Janeiro (CMNH) **45** *Menevia australis* holotype ♂, Brazil, Santa Catarina, Jaraguá do Sul (CUIC) **46** *Menevia australis* paratype ♂, Brazil, São Paulo, Guapiara, Paivinha, 800 m [photo courtesy CGCM] (CGCM) **47** *Menevia alurca* holotype ♂, Brazil, Maranhão, Feira Nova do Maranhão, 480 m (DZUP) **48** *Menevia alurca* paratype ♂, Brazil, Maranhão, Feira Nova do Maranhão, 480 m (CGCM) **49** *Menevia alurca* [transitional population] ♂, Brazil, Minas Gerais, Sete Lagoas, 720 m (USNM) **50** *Menevia alurca* [transitional population] ♂, Brazil, Minas Gerais, Paraopeba [photo courtesy CGCM] (OM) **51** *Menevia australis* paratype ♂, Brazil, São Paulo, Guapiara, Paivinha, 800 m [photo courtesy CGCM] (CGCM). Scale bar = 1 cm.

**Figures 52–57.**
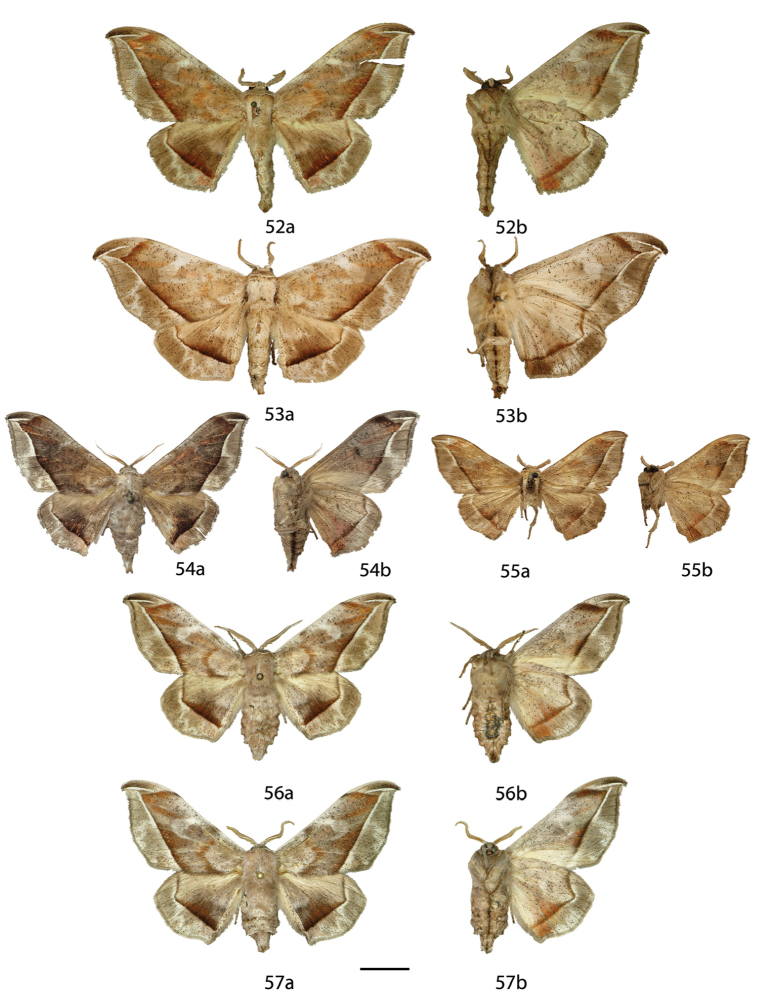
*Menevia plagiata* species-group [*plagiata* subgroup] female adults, **a** recto, **b** verso. **52** *Menevia plagiata* ♀, Brazil, Rio de Janeiro, Petrópolis [photo courtesy CGCM] (DZUP) **53** *Menevia plagiata* ♀ [“holotype” of manuscript name ‡*superba* Jones, photo courtesy NHMUK], Brazil, Rio de Janeiro, Corcovado, 800 ft. (NHMUK) **54** *Menevia alurca* ♀, Brazil, Maranhão, Feira Nova do Maranhão, 480 m (CGCM) **55** *Menevia alurca* ♀ [questionable], Argentina, Formosa (USNM) **56** *Menevia australis* paratype ♀, Brazil, Santa Catarina, São Bento do Sul, Rio Natal, 450 m [photo courtesy CGCM] (CGCM) **57** *Menevia australis* paratype ♀, Brazil, São Paulo, Guapiara, Paivinha, 800 m [photo courtesy CGCM] (CGCM). Scale bar = 1 cm.

###### Menevia alurca

Taxon classificationAnimaliaLepidopteraMimallonidae

Herbin & Mielke, 2014

Figs 47–50 , 54 , 55 , 85 , 99 ; Map 4

Menevia alurca Herbin & Mielke, 2014: 146–147; figs ♂ 48, 49, ♂ genitalia 50
Menevia ulcara ; Herbin and Mielke 2014: 147 misspelling

####### Type material.

**Holotype**, ♂: **BRAZIL: Maranhão**: genitalia prep. D. Herbin, ref H. 1008 [blue label]/ BRESIL Maranhao [Maranhão], Feira Nova do Maranhão, Retiro, 480 m. 4–12.XI.2011, -07°00'31"S, -46°26'41"W, C. Mielke leg. Coll. D. Herbin / Holotype ♂, *Menevia alurca* Herbin & Mielke det., Antenor 2014 [red label]/ DZ 15.727/ (DZUP) [examined]. Type locality: Brazil: Maranhão: Feira Nova do Maranhão.

**Paratypes**, 7 ♂ (6 specimens fit our concept of *Menevia alurca*, 1 specimen fits our concept of *Menevia delphinus* sp. n., see remarks under *Menevia delphinus* sp. n.), **BRAZIL: Maranhão**: 1 ♂, Feira Nova do Maranhão, Retiro, 46°26'41"W, -07°00'31"S, 480 m: 16–17.II.2013, C. Mielke leg., Paratypus *Menevia alurca* Herbin & C. Mielke det., 2014 [green label], Col. C. Mielke 26.882, St. Laurent diss.: 6-16-15:4 (CGCM) [examined]. Photos of all additional paratypes, each from the same locality and collector as the holotype and single physically examined paratype, were provided by Herbin and Mielke.

####### Additional specimens examined.

(4 ♂, 1 ♀ total) **BRAZIL: Distrito Federal**: 4 ♂, Estacão Florestal, Cabeça do Veado, 1100 m: 17.X.1971, 19.X.1971, 21.X.1971, 24.X.1971, E.G., I. & E.A. Munroe, St. Laurent diss.: 5-13-15:2 (CNC). **Maranhão**: 1 ♀, Feira Nova do Maranhão, Retiro, W46°26'41", S -07°00'31", 480 m: 23–24.XI.2013, C. Mielke leg., St. Laurent diss.: 7-28-15:2 (CGCM).

####### Additional transitional and questionable specimens examined.

(3 ♂, 1 ♀ total) **ARGENTINA**: 1 ♀, Formosa: data illegible except for locality, USNM-Mimal: 1203, St. Laurent diss.: 5-13-15:1 (USNM). **BRAZIL: Minas Gerais**: 2 ♂, Paraopeba: 27.II.1966 (OM). 1 ♂, Sete Lagoas, 720 m: 16.III.1974, V.O. Becker col., Col. Becker No. 413, USNM-Mimal: 2342, St. Laurent diss.: 5-13-15:3 (USNM).

####### Diagnosis.

*Menevia alurca* is distinguishable from all previous species in both sexes by the slate gray coloration suffused with deep blood-red, and the postmedial line, which is usually bent outward toward the wing margin at about three-quarters of its length. Additionally, the white band along the exterior of the postmedial line does not curve sharply toward the wing apex as it does in *Menevia plagiata*, but instead ends where it meets the apical dash. In this respect, *Menevia alurca* is similar to *Menevia australis* sp. n.; however, the white band usually juts out sharply just before approaching the apical dash in *Menevia alurca*, which is not seen in most other *Menevia*. The male genitalia however, should immediately distinguish this species from *Menevia australis* sp. n.; the phallus bears a prominent, elongated, pointed protuberance from the dorsal surface, not a rounded hump as in *Menevia australis* sp.n. The female genitalia differ from those of both *Menevia plagiata* and *Menevia australis* sp. n. by the small size, the very thin abdominal sclerotizations, and the mesally creased lamella antevaginalis.

####### Description.

**Male.** *Head*: Gray, eyes large comprising about two-thirds of head area, eyes bordered posteriorly by darker gray collar of scales reaching labial palpi, labial palpi very small, dorsally with darker scales contrasting with overall gray coloration. Antenna yellowish, scape and pedicel weakly tufted. *Thorax*: As for genus. Gray. *Legs*: As for genus. Tibial spurs moderate in length, scaled except for distal quarter. *Forewing dorsum*: Forewing length: 18–21.5 mm, avg.: 19.7 mm, n = 5. Triangular, apical half of outer margin concave, apex falcate. Ground color dark gray with predominance of deep red-brown or blood-red throughout medial area, overall lightly speckled by dark petiolate scales. Discal spot faintly marked by light gray, oblong shape; thin gray mark connects discal spot to costa. Apex marked by black scales above apical dash. Postmedial line usually bent outwards along three-fourths of its length, rarely nearly straight [especially in transitional population]. Submarginal area light gray, contrasting with much darker medial area, with whitish suffusion mesally forming a faint zigzag, postmedial lunule as distinct white band originating from apical dash, white band follows postmedial line from apex to roughly midway along postmedial line, resuming near anal margin. Antemedial line faint, brown, curved outwards. *Forewing venter*: As in forewing dorsum but generally much grayer, sometimes with pinkish hue, antemedial line absent, small black discal mark occasionally present. *Hindwing dorsum*: Subtriangular, anal angle accentuated, reddish coloration usually present near anal angle, bleeding into medial area, similar coloration and patterning overall as forewings, except postmedial lunule present as zigzagged mark, originating from white outer band outlining anterior bend of postmedial line, postmedial line weakly curved toward anterior wing margin, sometimes weakly concave mesally. *Hindwing venter*: Following similar pattern as forewing venter, but red coloration near anal angle much darker. *Abdomen*: As for genus, but somewhat stouter. Coloration a continuation of gray thoracic color. Dark, contrasting midventral stripe present. *Genitalia*: (Fig. 85) n = 4. Somewhat variable; tegumen ovoid or somewhat rectangular, sometimes weakly constricted near base of gnathos. Vinculum broad, somewhat quadrate ventrally. Valves broad at base, triangular, saccular edge of left valve with large triangular tooth proximal to transtilla, right valve with tooth slightly reduced in size, both valves with smaller mesal costal projection originating from central ridge of valve, mesal costal projection immediately above saccular edge teeth, apex of projection pointed toward saccular edge. Valves may be truncated distally, rounded apically. Uncus truncated apically, apex rounded. Gnathos as two prominent flattened, moderately sclerotized, flap-like, barely triangular, upward facing extensions with truncated apices. Apices usually form fingerlike projections of varying length. Juxtal processes roughly phallus length, moderately sclerotized, curving toward apex of phallus. Juxtal processes very thin, widening distally, covered in fine setae, especially apically. Base of phallus with paired, backwards facing, elongated, rounded, diverging fingerlike lobes. Phallus irregularly shaped, unevenly edged dorsum lacking an extensive dorsal ridge but with prominent, elongated, pointed setae covered projection, tip bent backwards. Left edge of rolled phallus uneven, forming extended projection, right edge usually with setae covered bulge laterally, base of sclerotized terminus of phallus with prominent ventral bump, angled away from distal end of phallus, distal tip of phallus separated into two distinct points of varying length. Vesica somewhat bag-like. **Female.** *Head*: As in male, dark gray scales surrounding eyes reduced. *Thorax*: As in male. *Legs*: As in male, tibial spurs longer. *Forewing dorsum*: Forewing length: 19.5–26 mm, n = 2. Maculation as in male but grayer with less red-brown, wing broader but still subtriangular. *Forewing venter*: As in forewing dorsum but lighter gray, dark discal mark present. *Hindwing dorsum*: As in male but more rounded, less triangular, postmedial line bent more sharply toward anterior wing margin. *Hindwing venter*: Following similar pattern as forewing venter, reddish-brown suffusion near anal angle much darker, contrasting. *Abdomen*: As in male but more robust. Sternite of VIII as pair of thin, nearly parallel, elongated, sclerotized bands. *Genitalia*: (Fig. 99) n = 2. Tergite of VIII robust, forming triangular, posteriorly directed arc. Apophyses anteriores shorter, thicker than apophyses posteriores. Lamella antevaginalis moderate in thickness, with mesal crease at ostium bursae. Ductus bursae short. Papillae anales elongated, subtriangular, covered in relatively long setae.

####### Distribution

(Map 4). *Menevia alurca* is primarily found in the Brazilian Cerrado, with records from Maranhão, Distrito Federal, and central Minas Gerais. The Minas Gerais population may represent an introgression zone with the similar *Menevia plagiata* of the Atlantic Forest. This species may also be present in Argentina, as suggested by the single questionable specimen discussed below.

####### Remarks.

Herbin and Mielke (2014) considered this species to be of the same size as *Menevia lucara*, although we found *Menevia alurca* larger on average. *Menevia plagiata*, which we show to be the closest species to *Menevia alurca* based on external characters and genitalia, was indeed mentioned in their diagnosis of *Menevia alurca*, but the authors did not provide differentiating characters other than that *Menevia plagiata* is larger. *Menevia alurca* is easily distinguished from *Menevia lucara* by all characters presented by Herbin and Mielke (2014). However, the male genitalia of *Menevia alurca* are nearly indistinguishable from those of *Menevia plagiata* and individuals of *Menevia alurca* that have straighter postmedial lines are externally very similar to *Menevia plagiata*.

Upon reviewing each of the seven paratypes of *Menevia alurca*, it became apparent that one paratype in the collection of Herbin (Bc-Her4848) does not belong to this species and was incorrectly included in the paratype series. The specimen in question has a continuous white band along the forewing postmedial line and lacks a midventral stripe on the abdomen, characters that allow us to identify this specimen as *Menevia delphinus* sp. n., another Cerrado species, described below.

External characteristics and male genitalia, namely, the phallus, of *Menevia alurca* is extremely similar to that of *Menevia plagiata*, thus these species are not readily differentiable based on male genitalia alone, as are most *Menevia* species. Furthermore, three specimens from two nearby localities in central Minas Gerais that we questionably determined as *Menevia alurca*, are somewhat intermediate in habitus between *Menevia alurca* from farther north and west (central Cerrado), and *Menevia plagiata* from farther east and southeast (Brazilian Atlantic Forest). These specimens display the predominance of darker coloration and the arrangement of markings near the forewing apices that we attribute to *Menevia alurca*. However, the forewing postmedial line is only weakly curved outwards, not bent so dramatically as in the types of *Menevia alurca*. These specimens are also quite large, much more in line with those of *Menevia plagiata*. The population from Minas Gerais is transitional in appearance between *Menevia alurca* and *Menevia plagiata*, and it is worth noting that the Cerrado and Atlantic Forest biomes converge there (IBGE 2004).

A female that we questionably assign to this species, from Formosa, Argentina, is extremely small and has a nearly straight forewing postmedial line. Although the size and postmedial line are not comparable to the other female *Menevia alurca*, the markings at the forewing apex, the predominance of red coloration, and genitalia (namely the mesally creased lamella antevaginalis), are all highly suggestive of *Menevia alurca*. Without more material from this locality, particularly males, it is impossible to reach conclusive determination about its identity. We decided to include this specimen in our material examined due to its collecting locality, which represents the only *Menevia* record from Argentina.

###### Menevia australis sp. n.

Taxon classificationAnimaliaLepidopteraMimallonidae

http://zoobank.org/F0C3F909-9FBF-4CBE-976E-FA9C903FE77D

Figs 45 , 46 , 51 , 56 , 57 , 86 , 100 ; Map 4

####### Type material.

**Holotype**, ♂: **BRAZIL: Santa Catarina**: Jaragua [Jaraguá do Sul], Santa Catharina, Brazil, 5 Sept 1934, Fritz Hoffmann/ PARATYPE *Menevia elegans* J. G. Franclemont/ St. Laurent diss.: 6-19-15:4/ Franclemont’s label refers to a MS name./ HOLOTYPE male *Menevia australis* St Laurent and Dombroskie, 2016 [handwritten red label]/ (CUIC). Type locality: Brazil: Santa Catarina: Jaraguá do Sul.

**Paratypes**, 19 ♂, 9 ♀: **BRAZIL: Santa Catarina**: 3 ♂, São Bento do Sul, Rio Vermelho, 850 m: 16.IX.1985, Mielke, Rank & Casagrande leg., DZ 32.713–32.715 (DZUP). 1 ♀, São Bento do Sul, Rio Natal, 26°20'2"S, 49°18'30"W, 450 m: 2.X.2014, I. Rank leg, Col. Mielke 28.976 (CGCM). 5 ♂, 2 ♀, Jaraguá do Sul: 13.IX.1934, 14.IX.1934, 15.IX.1934, 19.IX.1934, 6.X.1934, 8.X.1934, Fritz Hoffmann, Franclemont diss.: 1761, 1762, St. Laurent diss.: 4-25-15:3, 6-19-15:5 (CUIC). 1 ♂, 1 ♀, Nova Bremen [Dalbérgia], 250 m: 17.IX.1933, 26.III.1934, Carn. Mus. Acc. 11040, St. Laurent diss.: 7-30-15:1 (CMNH). 1 ♂, 1 ♀, no additional locality data: USNM-Mimal: 2434, 2851, St. Laurent diss.: 4-25-15:1 (USNM). **São Paulo**: 1 ♀, Campinas: 1.IV.1902, Coll. A. Hempel, Holland Collection, St. Laurent diss.: 4-25-15:4 (CMNH). 9 ♂, 3 ♀, Guapiara, Paivinha, 800 m: 12.IX.2007, 16–19.IX.2005, C. Mielke leg, 25.777, 25.811, 26.498, 26.567, 26.573, 27.126, 27.134, 28.833, 28.841, 28.869, 28.880, 28.906 Col. C. Mielke, C. Mielke diss.: 25.811 (CGCM). – All paratypes with the following yellow label: PARATYPE male/female *Menevia australis* St Laurent and Dombroskie, 2016.

####### Diagnosis.

*Menevia australis* is similar to *Menevia plagiata* in both sexes, but can be distinguished in both sexes by slightly broader wings, deeper brown coloration, and by the white band along the postmedial line, which terminates at the apex somewhat mesally along the length of the apical dash. The white band is either nearly perpendicular to the apical dash or it forms a roughly 45 degree angle with it. The white band may be somewhat curved toward the apex as in *Menevia plagiata*, but does not reach the apical tip of the wing. Male genitalia are easily recognized by the rounded hump or short triangular protuberance on the dorsal surface of the phallus, easily distinguishing the male genitalia from those of *Menevia plagiata* and *Menevia alurca*, which both have very elongated dorsal protuberances on the phallus. Additionally, the uncus is narrower and more triangular in *Menevia australis*. The VIII tergite in the female genitalia of *Menevia australis* forms a rounded arc and is not triangular as in *Menevia plagiata* and *Menevia alurca*.

####### Description.

**Male.** *Head*: Gray-brown or light brown, eyes large comprising about two-thirds of head area, eyes bordered posteriorly by darker brown collar of scales reaching labial palpi, labial palpi very small, dorsally with darker scales contrasting with overall gray coloration. Scape and pedicel tufted. *Thorax*: As for genus. Light gray-brown to light brown. *Legs*: As for genus. Tibial spurs small to moderate in length, almost entirely scaled. *Forewing dorsum*: Forewing length: 21.5–23 mm, avg.: 22.4 mm, n = 7. Triangular, apical half of outer margin concave, apex falcate. Ground color gray-brown with caramel brown or almost slate gray suffusion throughout medial area, reddish coloration near apex along apical interior of postmedial line, overall lightly speckled by dark petiolate scales. Discal spot faintly marked by light gray oblong shape, thin gray mark connecting discal spot to costa. Apex marked by black scales above apical dash, especially near apical tip. Postmedial line straight or weakly undulated, line black, strongly contrasting. Submarginal area light gray with whitish suffusion mesally forming faint or conspicuous zigzag, postmedial lunule as white band originating mesally from apical dash, white band follows postmedial line from apex to midway along postmedial line becoming zigzagged diffusion, white band resumes near anal margin. Antemedial line faint, brown, curved outwards. *Forewing venter*: As in forewing dorsum but grayer rather than brownish, antemedial line absent. *Hindwing dorsum*: Subtriangular, anal angle weakly accentuated, reddish coloration usually present near anal angle, similar coloration and patterning as forewings, except postmedial lunule present as zigzagged mark similar to zigzagged diffusion on forewing, mark originating from white outer band along first quarter of postmedial line, postmedial line sharply bent toward anterior wing margin, sometimes weakly concave mesally. *Hindwing venter*: Following similar pattern as forewing venter, but red coloration near anal angle much darker, almost brown. *Abdomen*: As for genus but somewhat elongated, nearly sphingiform. Coloration a continuation of gray thoracic color. Dark, contrasting midventral stripe present. *Genitalia*: (Fig. 86) n = 6. Tegumen ovoid, weakly constricted near base of gnathos. Vinculum rectangular, somewhat quadrate ventrally. Valves triangular, saccular edge of left valve with large triangular tooth proximal to transtilla, right valve with tooth slightly reduced in size, both valves with central tooth originating from central ridge of valve, tooth immediately above saccular edge teeth, apex of central tooth pointed toward saccular edge. Valves truncated distally, bent slightly outward near apex, rounded apically. Uncus narrow, triangular, apex rounded. Gnathos as two prominent flattened, moderately sclerotized, flap-like, somewhat triangular, outward-facing extensions with truncated apices. Apices usually form fingerlike projections of varying length. Juxtal processes roughly phallus length, moderately sclerotized, curving toward apex of phallus. Juxtal processes very thin, flattened, covered in fine setae. Base of phallus with paired, backwards facing, moderately elongated, rounded, diverging lobes. Phallus irregularly shaped, unevenly edged dorsum with prominent rounded, triangular, or somewhat rectangular setae covered hump. Left edge of rolled phallus uneven, forming hump; right edge usually with setae covered bulge laterally, base of sclerotized terminus of phallus with prominent ventral bump, angled away from distal end of phallus, distal tip of phallus separated into two distinct points of varying length. Vesica small, sac-like. **Female.** *Head*: As in male. *Thorax*: As in male. *Legs*: As in male, tibial spurs stouter. *Forewing dorsum*: Forewing length: 29.5–32 mm, avg: 30.3 mm, n = 1. Maculation as in male, wing broader, more ovoid, less triangular, outer white band of postmedial line intercepts apical dash mesally or dissipates before reaching mark, dark scaling above apical dash spread over length of apical dash. *Forewing venter*: As in forewing dorsum but usually grayer. *Hindwing dorsum*: As in male but more rounded, less triangular. *Hindwing venter*: Following similar pattern as forewing venter, reddish-brown suffusion near anal angle much darker, contrasting. *Abdomen*: As in male but more robust. Sternite of VIII as pair of elongated, broad or very narrow sclerotized bands curving toward each other near anterior edge of VIII segment, but not converging. *Genitalia*: (Fig. 100) n = 2. Tergite of VIII forms curved, rounded, posteriorly directed arc. Apophyses anteriores shorter than apophyses posteriores. Lamella antevaginalis thin, C-shaped, weakly notched mesally near ostium bursae. Ductus bursae narrow. Papillae anales subtriangular, covered in setae.

####### Distribution

(Map 4). This new species is so far known only from southeastern Brazil in the northeast of Santa Catarina state and eastern São Paulo state. *Menevia australis* is likely present in intervening eastern Paraná as well.

####### Etymology.

*Menevia australis* is named for its southerly distribution, which among *Menevia*, is only shared with *Menevia magna*. Additionally, *Menevia australis* seems to represent the southeast most extension of the *Menevia plagiata* species complex, replacing *Menevia plagiata* farther southeast.

####### Remarks.

*Menevia australis* is the southeasternmost species of the *Menevia plagiata* complex and is quite difficult to separate from true *Menevia plagiata* without a genitalia dissection or geographic information. However, upon thorough examination of the male and female genitalia, external diagnostic characters became readily apparent and have been presented above in the diagnosis. Additionally, the allopatric distribution of these species suggests that they are two separate species, albeit very closely related. The allopatric distribution patterns of *Menevia australis* and *Menevia plagiata* are not unique, similar allopatry was shown in two closely related Saturniidae by Mielke et al. (2012) wherein there is a distinct gap in eastern São Paulo state. This gap is probably not due to lack of collecting as the region has been extensively sampled (C. Mielke pers. comm.).

Both *Menevia australis* and *Menevia franclemonti* sp. n. described below, were originally recognized as distinct by J. G. Franclemont, and given manuscript names, but never formally described. The holotype and some paratypes of *Menevia australis* (all from Jaraguá do Sul, Santa Catarina, Brazil) bear labels reading “PARATYPE,” “HOLOTYPE,” or “ALLOTYPE” with Franclemont’s manuscript name *Menevia* ‡*elegans*. In addition to our holotype and paratype labels, we have placed labels on these specimens stating that Franclemont’s labels represent a manuscript name.

##### *vulgaris* subgroup

The *vulgaris* subgroup contains *Menevia vulgaris* sp. n., *Menevia franclemonti* sp. n., *Menevia vulgaricula* sp. n., *Menevia cordillera* sp. n., and *Menevia delphinus* sp. n. and is diagnosed by the continuous white band along the outer margin of the postmedial line and by the lack of a midventral abdominal stripe. The species boundaries in the *vulgaris* subgroup are clearer than in the previous subgroup.

###### Menevia vulgaris sp. n.

Taxon classificationAnimaliaLepidopteraMimallonidae

http://zoobank.org/AC0AD0CC-462E-4B81-BDEB-B1D5BFFD19BE

**Map 5.**
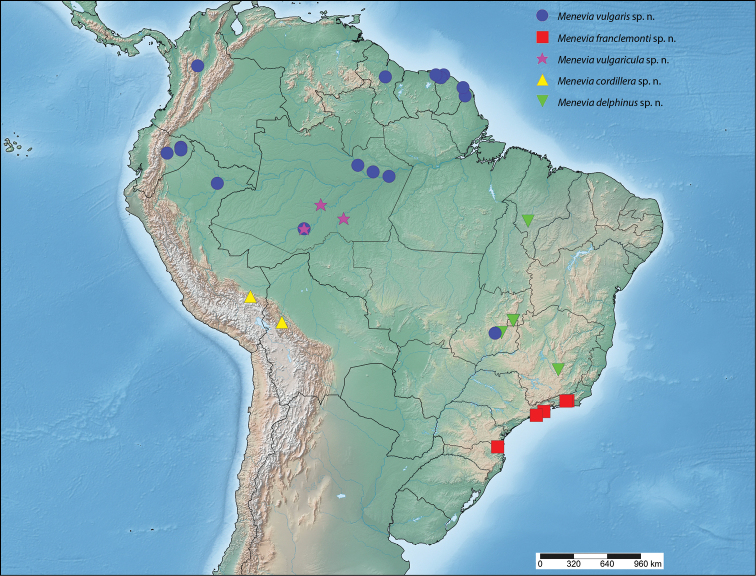
*Menevia plagiata* species-group [*vulgaris* subgroup].

**Figures 58–66.**
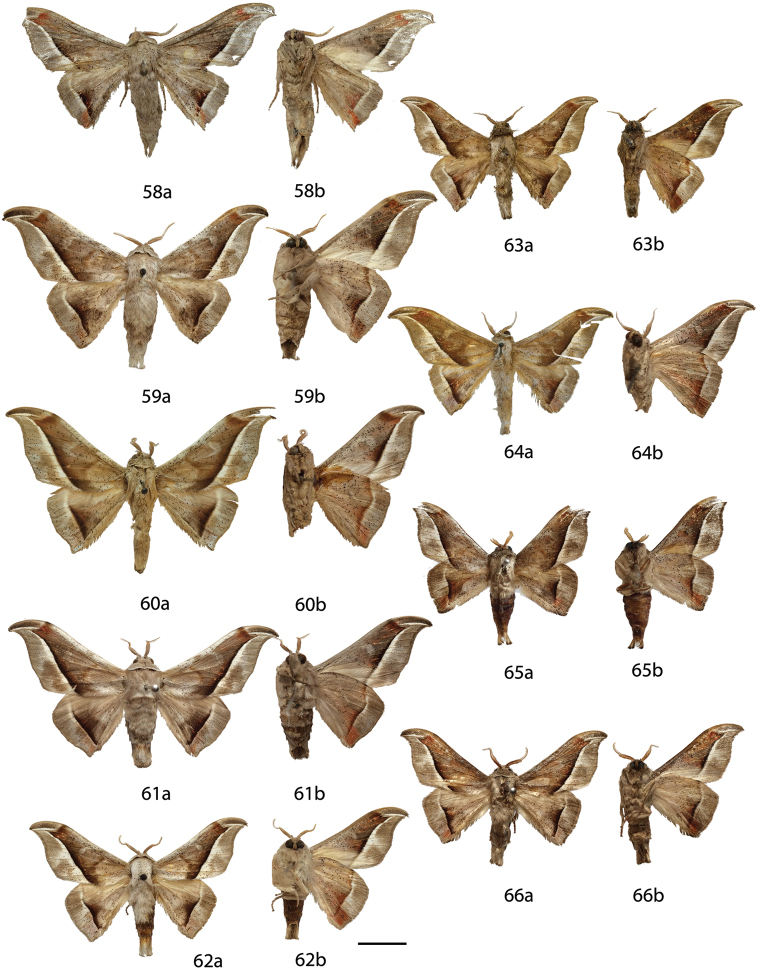
*Menevia plagiata* species-group [*vulgaris* subgroup] male adults, **a** recto, **b** verso. **58** *Menevia vulgaris* holotype ♂, French Guiana, St. Laurent du Maroni, Piste de Paul Isnard, 52 m (CUIC) **59** *Menevia vulgaris* paratype ♂, Brazil, Huitanaã, Rio Purus (CMNH) **60** *Menevia franclemonti* holotype ♂, Brazil, Santa Catarina, Jaraguá do Sul (CUIC) **61** *Menevia franclemonti* paratype ♂, Brazil, Rio de Janeiro, Teresópolis, 350 m (USNM) **62** *Menevia vulgaricula* holotype ♂, Brazil, Huitanaã, Rio Purus (CMNH) **63** *Menevia cordillera* holotype ♂, Peru, San Gabán, 2500 ft (NHMUK) **64** *Menevia cordillera* paratype ♂, Bolivia, Río Zongo, 750 m (USNM) **65** *Menevia delphinus* holotype ♂, Brazil, Distrito Federal, Planaltina, 1000 m (CPAC) **66** *Menevia delphinus* paratype ♂, Brazil, Distrito Federal, Planaltina, 1000 m (USNM). Scale bar = 1 cm.

**Figures 67–71.**
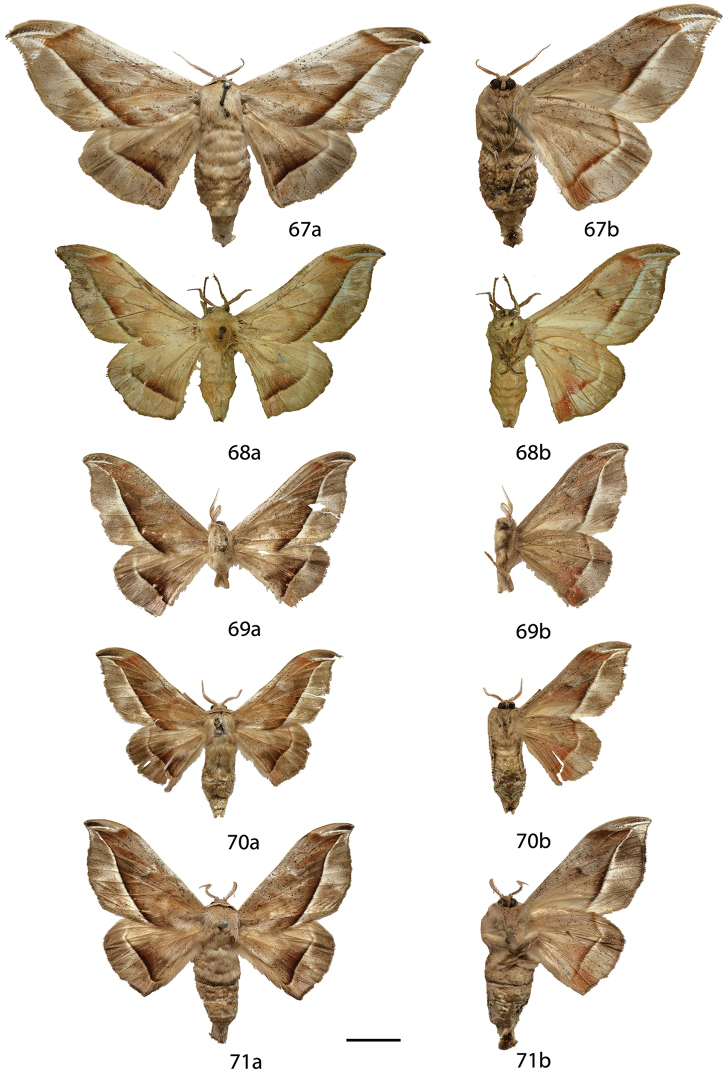
*Menevia plagiata* species-group [*vulgaris* subgroup] female adults, **a** recto, **b** verso. **67** *Menevia vulgaris* paratype ♀, Guyana, Omai (USNM) **68** *Menevia franclemonti* paratype ♀, Brazil, Rio de Janeiro, Teresópolis, Barreira [photo courtesy CGCM] (DZUP) **69** *Menevia vulgaricula* paratype ♀, Brazil, Amazonas, Rio Madeira (USNM) **70** *Menevia vulgaricula* ♀ [questionable], Brazil, Pará (NHMUK) **71** *Menevia delphinus* paratype ♀, Brazil, Distrito Federal, Planaltina, 1000 m (CPAC). Scale bar = 1 cm.

Figs 58 , 59 , 67 , 87 , 101 ; Map 5

####### Type material.

**Holotype**, ♂: **FRENCH GUIANA**: GUYANE, St. Laurent-du-Maroni, Piste de Paul Isnard, N05°22.562', W53°57.678', 8.iii.2011, 52 m., P. Sammut/ *MENEVIA plagiataplagiata* (Walker, 1855)/ St. Laurent diss.: 9-2-15:2/ HOLOTYPE male *Menevia vulgaris* St Laurent and Dombroskie, 2016 [handwritten red label]/ (CUIC). Type locality: French Guiana: St. Laurent du Maroni.

**Paratypes**, 30 ♂, 10 ♀: **BRAZIL: Amazonas**: 8 ♂, Reserva Ducke, km. 26 Manaus-Itacoatiara Highway: 15.IV.1972, 16.IV.1972, 19.IV.1972, 15.V.1972, 16.V.1972, E.G., I. & E.A. Munroe, St. Laurent diss.: 4-25-15:8, 8-3-15:1 (CNC). 1 ♂, Itacoatiara Airport: 6.V.1972, E.G., I. & E.A. Munroe (CNC). 1 ♂, Mirapinima, Rio Negro: 8.IV.1972, E.G., I. & E.A. Munroe (CNC). 7 ♂, Hyutanahan [Huitanaã], Rio Purus: II.1922, III.1922, S.M. Klages, Carn. Mus. Acc. 6963, 7088, St. Laurent diss.: 4-25-15:10, 7-20-15:1, 7-20-15:2, 7-23-15:1 (CMNH). **Goiás**: 1 ♂, Campinas [Goiânia]: I.1934, Coll. R. Spitz, Rothschild Bequest BM 1939-1, BMNH(E) 1378754 St. Laurent diss.: 6-29-15:9 (NHMUK). **Pará**: 1 ♂, 1 ♀, Likely Belém: Collector Moss, USNM-Mimal: 2592, 2597, St. Laurent diss.: 9-7-14:4, 4-25-15:12 (USNM). **COLOMBIA**: 3 ♂, Antioquia, Nari [Nare?] River: Collection Frank Johnson, USNM-Mimal: 2586, St. Laurent diss.: 4-25-15:11 (USNM); USNM-Mimal: 2587, St. Laurent diss.: 6-19-15:3 (USNM); USNM-Mimal: 2588 (USNM). 1 ♀, “Colombia, S.A.”: Felipe Ovalle, Q., Ac. 33501 (AMNH). **ECUADOR**: 1 ♂, Napo [Orellana], near Pompeya (Yasuni Nat. Pk.), 00°38–40'S, 76°22–27'W, 280 m: 17–30.X.1998, D. Robacker, M.H. Evans Collection, St. Laurent diss.: 9-7-14:5 (CUIC). 1 ♂, Napo [Orellana], Parque Nacional Yasuni, 1 km. SE PUCE station, edge of virgin forest: 13.V.1996, Jan Hillman (CMNH). 1 ♂, Napo [Orellana], Parque Nacional Yasui, Rio Natali, 20 km. S PUCE station, near edge of virgin forest: 16.V.1996, Jan Hillman, St. Laurent diss.: 7-20-15:3 (CMNH). 1 ♀, Napo, Rio Arajuno, Camp Dayuma, 1°05'35"S, 77°35'07"W, 390–420 m: 19–22.IV.1990, S.J. Weller, P. Batra, & M.J. Ryan, St. Laurent diss.: 7-21-15:3 (USNM). **FRENCH GUIANA**: 1 ♂, 1 ♀, Mana River: V.1917, Acc. 6008, St. Laurent diss.: 4-25-15:9 (CMNH). 1 ♀, St. Laurent du Maroni: Collection Wm Schaus, USNM-Mimal: 2591, St. Laurent diss.: 4-25-15:13 (USNM). 1 ♀, Pied Saut, Oyapok River: III.1918, S.M. Klages, C.M. Acc. 6173, St. Laurent diss.: 7-21-15:1 (CMNH). 1 ♀, St. Jean du Maroni: II, Collection Le Moult, Dognin Collection, USNM-Mimal: 2590 (USNM). 1 ♀, Camp Patawa, Kaw Mountains, 36 km. E. of Roura: 21.XII.1997, leg. I.L. Finkelstein (MGCL). **GUYANA**: 1 ♀, Omai, Br. Guiana: Collection Wm Schaus, USNM-Mimal: 2589 (USNM). **PERU**: 1 ♀, Iquitus [Iquitos]: Dr. Luka Kassarov donation to FSCA collection (FSCA). **SURINAME**: 3 ♂, Moengo, Boven Cottica River: 25.V.1927, Cornell Univ. Lot 760, Sub 79, St. Laurent diss.: 12-10-13:2 (CUIC); 26.V.1927, Cornell Univ. Lot 760, Sub 80 (CUIC). **BRAZIL-PERU BORDER**: 1 ♂, “Amazons, Peru-Brazil”: 1930, H.S. Parish (CUIC). – All paratypes with the following yellow label: PARATYPE male/female *Menevia vulgaris* St Laurent and Dombroskie, 2016.

####### Diagnosis.

*Menevia vulgaris* is recognizable from all previous species by the replacement of the wing margin swept postmedial lunule with a continuous white band along the entire length of the postmedial line. This species is quite large for the genus, with highly elongated acutely triangular forewings and triangular hind wings. Sexual dimorphism is well developed, with females having broader, less triangular, but still highly elongated forewings. Male genitalia are easily recognized by the somewhat cylindrical shape of the phallus with an irregularly edged dorsum lacking an extensive dorsal ridge, but usually with a weak anteriorly situated bulge. The juxtal processes are wide and flattened, but only weakly sclerotized; the proximal lobes of the phallus are also very broad, not elongated or fingerlike as in most other species. The combination of these genitalia characters also distinguish *Menevia vulgaris* from the following four new species described below. Size should also be sufficient in separating *Menevia vulgaris* from all sympatric species, as *Menevia vulgaris* is always the largest species within its range. *Menevia vulgaris* is most similar in appearance and size to *Menevia franclemonti* sp. n. described below, but is easily differentiated from this species by the more sharply angled hindwing postmedial line and by the more uneven dorsum of the phallus. Geography should also be sufficient for separating *Menevia vulgaris* from *Menevia franclemonti* sp. n., as the latter species is restricted to southeastern Brazil, outside the largely Amazonian distribution of *Menevia vulgaris*.

####### Description.

**Male.** *Head*: Gray, eyes large comprising about two-thirds of head area, eyes bordered posteriorly by darker gray collar of scales reaching labial palpi, labial palpi very small, segments weakly defined ventrally, dorsally with darker scales contrasting with overall gray coloration. Scape and pedicel tufted. *Thorax*: As for genus. Light gray. *Legs*: As for genus. Tibial spurs very small, short, almost entirely scaled. *Forewing dorsum*: Forewing length: 22–28 mm, avg.: 25.3 mm, n = 24. Elongated, acutely triangular, apical half of outer margin concave, apex falcate. Ground color gray with darker gray, brown, or reddish brown suffusion throughout medial area, especially near interior edge of postmedial line, pinkish red to blood red coloration near apex along apical interior of postmedial line, overall lightly speckled by dark petiolate scales. Discal spot faintly marked by light gray oblong shape, thin gray mark connecting discal spot to costa. Apex marked by black scales above extended apical dash. Postmedial line weakly curved to follow outline of wing margin, line black, strongly contrasting. Submarginal area light gray with whitish suffusion mesally, postmedial lunule as distinct white band originating from apical dash, white band follows postmedial line from apex to posterior wing margin. Antemedial line faint, brown, curved outwards. *Forewing venter*: As in forewing dorsum but antemedial area lighter gray, more contrasting, sometimes with blood-red suffusion, antemedial line absent, small black discal spot occasionally present. *Hindwing dorsum*: Triangular, anal angle weakly accentuated, reddish coloration near anal angle, similar coloration and patterning as forewings, antemedial line absent, postmedial line sharply bent toward anterior wing margin, concave mesally. *Hindwing venter*: Following similar pattern as forewing venter, but red coloration near anal angle much darker, discal mark sometimes present. *Abdomen*: As for genus, but somewhat elongated, nearly sphingiform. Coloration a continuation of gray thoracic color. Midventral stripe absent. *Genitalia*: (Fig. 87) n = 15. Tegumen elongated, ovoid or rounded rectangular, constricted near base of gnathos. Vinculum broad, rounded ventrally. Valves relatively narrow, saccular edge of left valve with large triangular tooth proximal to transtilla, right valve with tooth slightly reduced in size, both valves with smaller central tooth originating from central ridge of valve, tooth immediately above saccular edge teeth, apex of central tooth pointed toward saccular edge. Valves rounded or somewhat pointed apically. Uncus truncated apically, apex rounded. Gnathos as two prominent flattened, lightly sclerotized, flap-like, somewhat triangular, outward facing extensions with highly truncated apices. Apices usually form fingerlike projections of varying length. Juxtal processes roughly phallus length, lightly sclerotized, curving toward apex of phallus. Juxtal processes very thin, sclerotization weakening to become more membranous, covered in fine setae. Base of phallus with paired, backwards facing, short, rounded, diverging lobes. Phallus cylindrical, irregularly edged dorsum lacking an extensive dorsal ridge, covered in setae. Left edge of rolled phallus uneven but without ridge-like process, usually with weak, anteriorly situated setae covered bulge, distal tip of phallus separated into two distinct points of varying length. Vesica elongate, sac-like, originating from progressively weakened sclerotization of nearly vertical edge of phallus. **Female.** *Head*: As in male but collar of scales bordering eyes and palpi much lighter, less contrasting. *Thorax*: As in male. *Legs*: As in male, tibial spurs very small, only distal tip without scales. *Forewing dorsum*: Forewing length: 27.5–39 mm, avg. 34.6 mm, n = 9. Maculation as in male, wing broader and more elongate, less triangular, less falcate, pinkish hue may be replaced by deeper red brown, postmedial line usually straighter, antemedial line fainter. *Forewing venter*: As in forewing dorsum but sometimes with blood-red suffusion, antemedial line absent, thin black discal mark occasionally present. *Hindwing dorsum*: As in male but more rounded, less triangular, postmedial line straight, not concave mesally, but still sharply bent toward anterior wing margin. *Hindwing venter*: Following similar pattern as forewing venter except lighter, reddish suffusion near anal angle much darker, contrasting. *Abdomen*: As in male but more robust. Sternite of VIII as pair of elongated sclerotized bands converging into thicker, irregularly shaped sclerotization near anterior margin of VIII, forming a “V” or “U.” *Genitalia*: (Fig. 101) n = 4. Tergite of VIII smoothly curved or weakly triangular, with or without membranous gap mesally, which may be accentuated as posteriorly directed arc. Apophyses anteriores shorter or about same length as apophyses posteriores. Lamella antevaginalis quadrate, notched mesally near ostium bursae, anterior edge somewhat irregular. Ductus bursae moderately long. Papillae anales elongated, covered in relatively long setae.

####### Distribution

(Map 5). *Menevia vulgaris* is found throughout northern South America, in the Guyanas, Suriname, Colombia, Ecuador, Peru and the Brazilian states of Amazonas, Pará, and Goiás. This species is predominantly Amazonian in distribution, but the record from the Cerrado in Goiás suggests it is more widespread in various habitats.

####### Etymology.

*Menevia vulgaris* (=vulgaris Latin, meaning commonplace) is named for its wide distribution and apparent commonness.

####### Remarks.

This species is usually the most frequently represented *Menevia* in collections and apparently quite common throughout its broad distribution. This new species is usually misidentified as *Menevia plagiata* and has long been considered conspecific with the southeastern Brazilian species redescribed above. The taxonomic issues surrounding the name *plagiata* have been explained in the remarks of that species, and the designation of the neotype of *Menevia plagiata* resolves any previous identification problems and allows recognition of these two very distinct taxa.

Walker’s (1855) vague original description of *Menevia plagiata* could arguably have been associated with the external characters of our concept of *Menevia plagiata*, *Menevia vulgaris*, *Menevia australis*, or what we describe below as *Menevia franclemonti* sp. n. However, we have resolved any ambiguity regarding the application of the name by designating the neotype of *Menevia plagiata* above. The wide-ranging Amazonian species, *Menevia vulgaris*, which is apparently absent in the biome inhabited by the other three species, therefore remained undescribed until now.

Currently, the name *Menevia vulgaris* can now only be associated with the large species present in the Amazonian and Cerrado regions. Diagnostic characters given before, particularly size and male genitalia, are adequate for identifying this species. Other names, including *plagiata* and the various new species described below, are associated with either allopatric or much smaller species.

As with other wide-ranging *Menevia* species, such as *Menevia lantona* and *Menevia lucara*, there is a degree of geographic variation, although this variation is less obvious than in these two species. As in other species-groups, *Menevia vulgaris* from Colombia have more robust male genitalia, except that the juxtal processes are thinner, the valve teeth smaller, and the gnathos elongations shorter. Externally, however, the examined males do not differ from other populations except for their overall slightly larger size. Similar shortened gnathos elongations were found in the males from Ecuador. These males and a single examined female from Ecuador were all smaller than males and females from other populations. However, the lack of differences of the phallus in Ecuadorian males compared to those from other locations suggests that the smaller size and some noted differences found in the single female dissection, namely the shape of the highly variable ventral sclerotized bands on VIII, are not grounds to consider the Ecuadorian population a distinct taxon. Another variation worth noting is in the single Peruvian female specimen, which has the reddish maculation on all wings replaced by deeper brownish-red.

**Figures 72–81.**
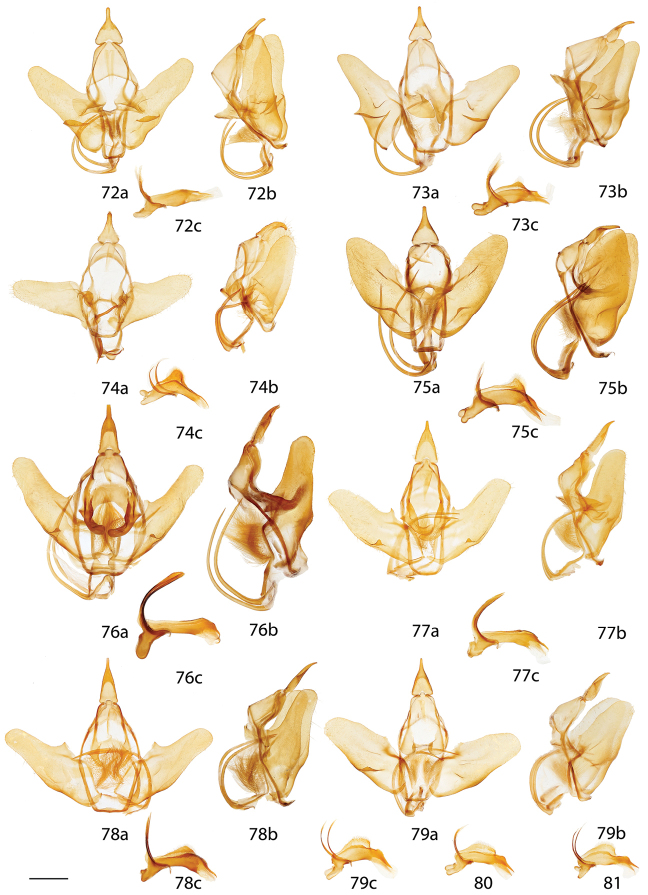
*Menevia* male genitalia, **a** ventral, **b** lateral, **c** phallus [except Figures 80, 81]. **72** *Menevia lantona*, French Guiana, Pied Saut, Oyapok River [St. Laurent diss.: 3-7-15:2] (CMNH) **73** *Menevia rosea* holotype, Ecuador, Napo, Simón Bolívar, 1200 m [St. Laurent diss.: 3-7-15:9, 73b inverted horizontally] (CMNH) **74** *Menevia torvamessoria* holotype, Peru, Puno, La Unión, 2000 ft [St. Laurent diss.: 6-29-15:4, 74b inverted horizontally] (NHMUK) **75** *Menevia magna* holotype, Brazil, Santa Catarina, São Bento do Sul, Rio Natal, 450 m [St. Laurent diss.: 6-16-15:1] (DZUP) **76** *Menevia lucara*, French Guiana, Mana River [St. Laurent diss.: 2-5-15:4] (CMNH) **77** *Menevia menapia* holotype, Guatemala, Cayuga [St. Laurent diss.: 9-7-14:1] (USNM) **78** *Menevia mielkei* paratype, Brazil, Rio de Janeiro, Cachoeiras de Macacu, 700 m [St. Laurent diss.: 2-5-15:11] (USNM) **79** *Menevia ostia*, Costa Rica, Guanacaste, Tajo Angeles, 540 m, 10-SRNP-4309 [St. Laurent diss.: 7-14-15:2] (USNM) **80** *Menevia ostia* phallus, French Guiana, Nouveau Chantier [St. Laurent 3-24-15:7] (USNM) **81** *Menevia ostia* phallus, Brazil, Espírito Santo, Linhares [St. Laurent 3-24-15:11] (USNM). Scale bar = 1 mm.

**Figures 82–86.**
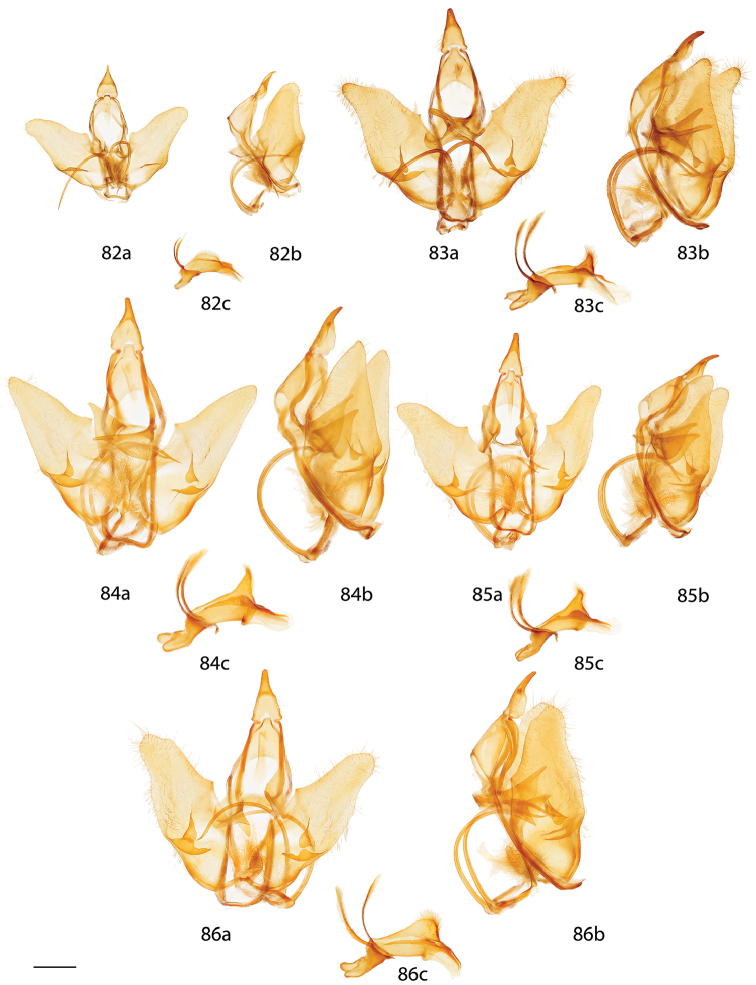
*Menevia* male genitalia, **a** ventral, **b** lateral, **c** phallus. **82** *Menevia pallida* paratype, Brazil, Maranhão, Feira Nova do Maranhão, 480 m [St. Laurent diss.: 6-16-15:3] (CGCM) **83** *Menevia plagiata* neotype, Brazil, Rio de Janeiro, Teresópolis [St. Laurent diss.: 9-2-15:1] (NHMUK) **84** *Menevia plagiata*, Brazil, Rio de Janeiro [St. Laurent diss.: 4-25-15:2] (CMNH) **85** *Menevia alurca* paratype, Brazil, Maranhão, Feira Nova do Maranhão, 480 m [St. Laurent diss.: 6-16-15:4] (CGCM) **86** *Menevia australis* holotype, Brazil, Santa Catarina, Jaraguá do Sul [St. Laurent diss.: 6-19-15:4] (CUIC). Scale bar = 1 mm.

**Figures 87–89.**
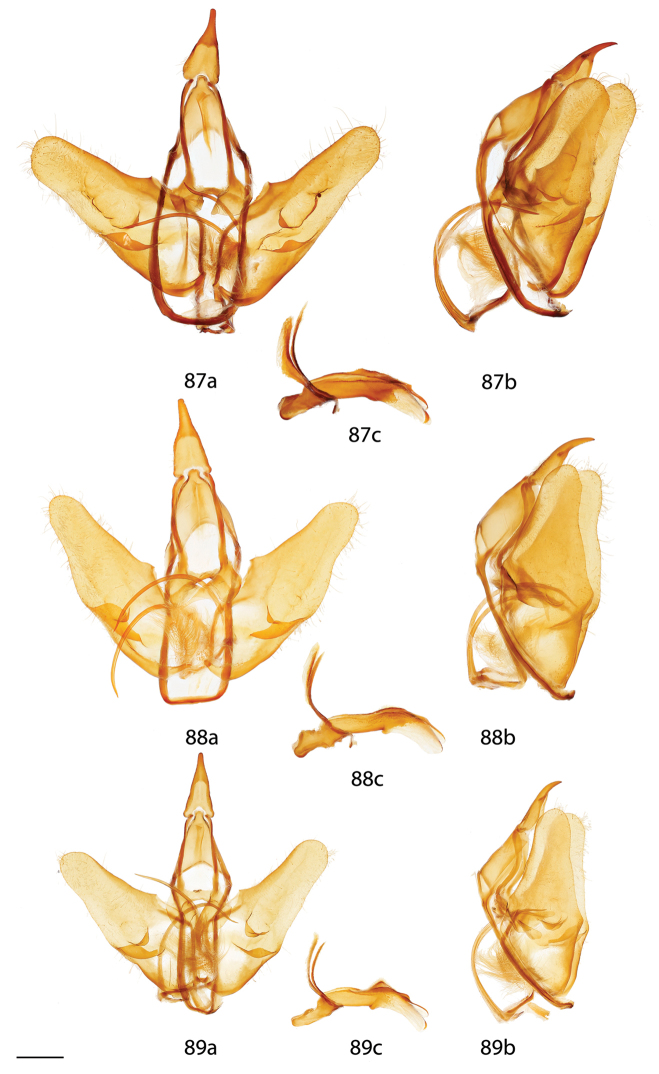
*Menevia* male genitalia, **a** ventral, **b** lateral, **c** phallus. **87** *Menevia vulgaris* holotype, French Guiana, St. Laurent du Maroni, Piste de Paul Isnard, 52 m [St. Laurent diss.: 9-2-15:2] (CUIC) **88** *Menevia franclemonti* holotype, Brazil, Santa Catarina, Jaraguá do Sul [St. Laurent diss.: 4-25-15:5] (CUIC) **89** *Menevia vulgaricula* holotype, Brazil, Huitanaã, Rio Purus [St. Laurent diss.: 6-19-15:1] (CMNH). Scale bar = 1 mm.

**Figures 90–91.**
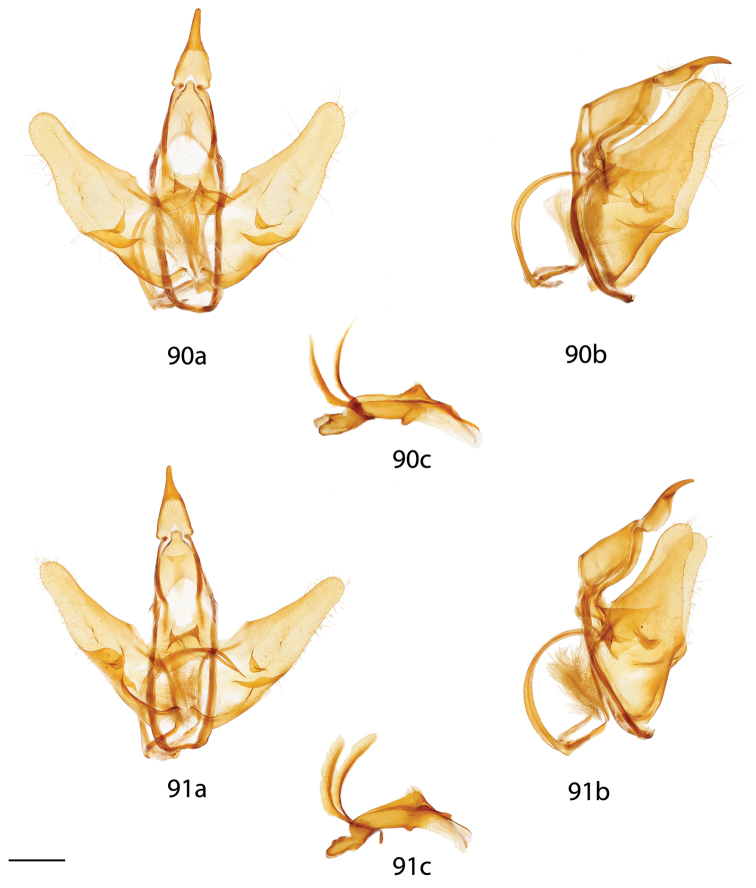
*Menevia* male genitalia, **a** ventral, **b** lateral, **c** phallus. **90** *Menevia cordillera* holotype, Peru, San Gabán, 2500 ft [St. Laurent diss.: 6-29-15:8] (NHMUK) **91** *Menevia delphinus* holotype, Brazil, Distrito Federal, Planaltina, 1000 m [St. Laurent diss.: 9-3-15:1] (CPAC). Scale bar = 1 mm.

**Figures 92–97.**
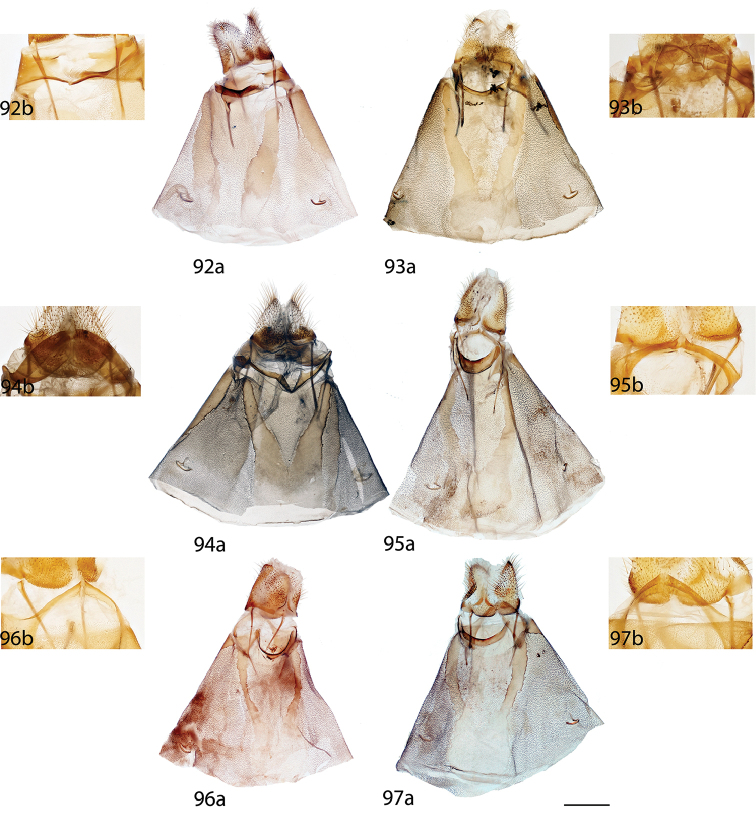
*Menevia* female genitalia, **a** ventral, **b** VIII tergite detail. **92** *Menevia lantona*, French Guiana, St. Jean du Maroni, [St. Laurent diss.: 7-8-15:1] (USNM) **93** *Menevia magna* paratype, Brazil, Santa Catarina, Dalbérgia [St. Laurent diss.: 3-7-15:15] (CUIC) **94** *Menevia lucara*, French Guiana, Kourou Forest [St. Laurent diss.: 5-22-15:1] (MNHN) **95** *Menevia ostia*, Costa Rica, Guanacaste, Mena Central, 345 m, Voucher: 01-SRNP-24319 [St. Laurent diss.: 4-20-15:9] (USNM) **96** *Menevia parostia* holotype, unknown locality [St. Laurent diss.: 4-20-15:7] (USNM) **98** *Menevia pallida* [questionable], Brazil, Minas Gerais, Lassance [St. Laurent diss.: 4-20-15:6] (CUIC). Scale bar = 1 mm.

**Figures 98–100.**
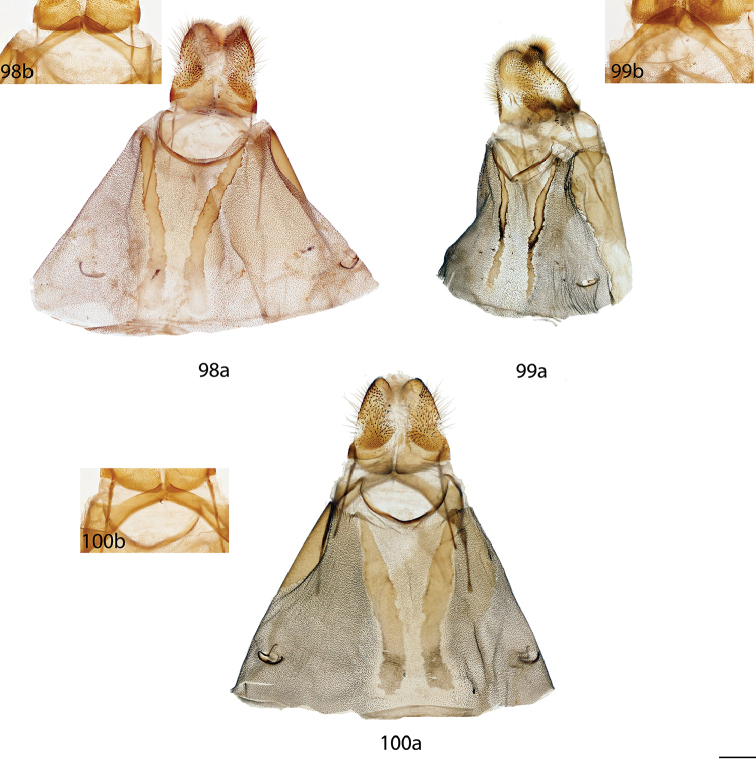
*Menevia* female genitalia, **a** ventral, **b** VIII tergite detail. **98** *Menevia plagiata*, Brazil, Rio de Janeiro [St. Laurent diss.: 7-28-15:1] (AMNH) **99** *Menevia alurca*, Brazil, Maranhão, Feira Nova do Maranhão [St. Laurent diss.: 7-28-15:2] (CGCM) **100** *Menevia australis* paratype, Brazil, Santa Catarina, Jaraguá do Sul [St. Laurent diss.: 4-25-15:3] (CUIC). Scale bar = 1 mm.

**Figures 101–103.**
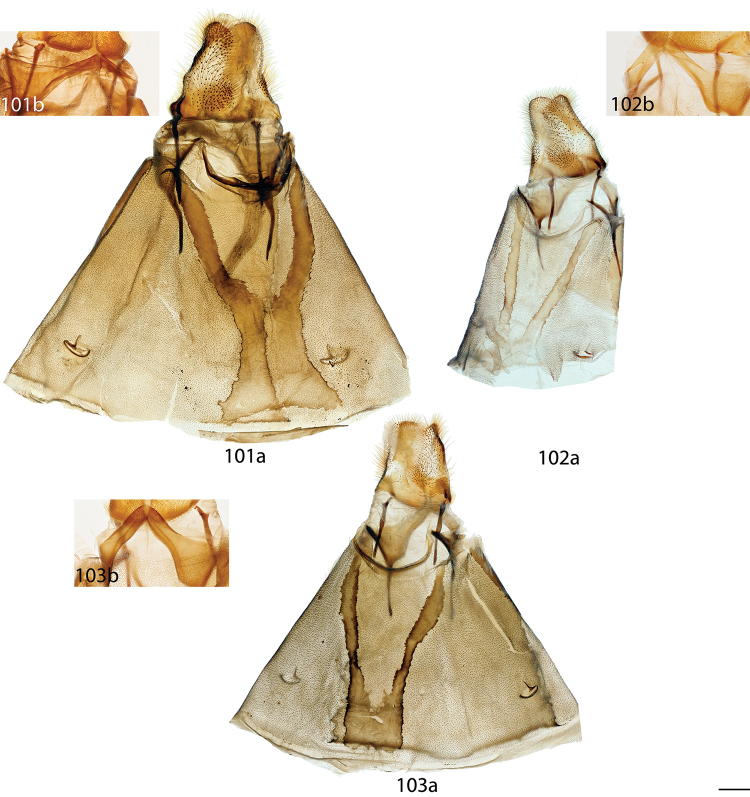
*Menevia* female genitalia, **a** ventral, **b** VIII tergite detail. **101** *Menevia vulgaris* paratype, French Guiana, St. Laurent du Maroni [St. Laurent diss.: 4-25-15:13] (USNM) **102** *Menevia vulgaricula* paratype, Brazil, Amazonas, Rio Madeira [St. Laurent diss.: 4-25-15:15] (USNM) **103** *Menevia delphinus* paratype, Brazil, Distrito Federal, Planaltina [St. Laurent diss.: 4-2-15:4] (CPAC). Scale bar = 1 mm.

###### Menevia franclemonti sp. n.

Taxon classificationAnimaliaLepidopteraMimallonidae

http://zoobank.org/2CB186E2-CBFA-46B9-94A4-4FA8A3056F9E

Figs 60 , 61 , 68 , 88 ; Map 5

####### Type material.

**Holotype**, ♂: **BRAZIL: Santa Catarina**: Jaragua [Jaraguá do Sul], Santa Catharina, Brazil, 10 Nov 1934, Fritz Hoffmann/ PARATYPE *Menevia falco* J. G. Franclemont/ St. Laurent diss.: 4-25-15:5/ Franclemont’s label refers to a MS name./ HOLOTYPE male *Menevia franclemonti* St Laurent and Dombroskie, 2016 [handwritten red label]/ (CUIC). Type locality: Brazil: Santa Catarina: Jaraguá do Sul.

**Paratypes**, 10 ♂, 1 ♀: **BRAZIL: Rio de Janeiro**: 1 ♂, Petrópolis, Independência, 900 m: 15.XI.1939, Gagarin leg., ex. col. Gagarin, DZ 32.717 (DZUP). 1 ♂, Petrópolis: 17.X.1960, Gagarin leg., ex. coll Gagarin, DZ 32.718 (DZUP). 2 ♂, Teresópolis, Barreira, 350 m: 30.X.1956–3.XI.1956, 1.XII.1956, Pearson H&G, HRP No. 1070, USNM-Mimal: 2272, 2273, St. Laurent diss.: 4-25-15:6 (USNM). 2 ♂, 1 ♀, Teresópolis, Barreira: 12.XI.1955, 17.XI.1955, 19.XI.1955, ex. col. Gagarin, DZ 32.719–32.721 (DZUP). **Santa Catarina**: 2 ♂, Jaraguá do Sul: 17.XI.1934, 20.XI.1934, Fritz Hoffmann, Franclemont diss.: 1760 (CUIC). **São Paulo**: 1 ♂, Juqueí: 20.III.1976, ex. coleção A. Cardoso, DZ 32.716 (DZUP). 1 ♂, Ubatuba, 10 m: 13-20.XII.1963, Prof. Heinz Ebert, St. Laurent diss.: 4-25-15:7 (CMNH). – All paratypes with the following yellow label: PARATYPE male/female *Menevia franclemonti* St Laurent and Dombroskie, 2016.

####### Diagnosis.

Very similar to *Menevia vulgaris*, but distinguished by the usually warmer brown hue rather than gray, more sharply falcate forewings, and a more smoothly curving postmedial line on the hindwing, which does not sharply bend toward the anterior wing margin. The male genitalia are also unique in that the phallus is thin and smooth, not irregularly shaped dorsally, with the sclerotized portion of the distal end of the phallus diagonal rather than vertical as in *Menevia vulgaris*. Furthermore, the base of the sclerotized terminus of the phallus, on the venter, has a prominent bump, angled away from the distal end of the phallus. *Menevia franclemonti* replaces *Menevia vulgaris* in the Brazilian Atlantic Forest.

####### Description.

**Male.** *Head*: Gray, eyes large comprising about two-thirds of head area, eyes bordered posteriorly by light brownish gray collar of scales reaching labial palpi, labial palpi very small, dorsally with darker scales contrasting with overall gray coloration. Scape and pedicel tufted. *Thorax*: As for genus. Light gray to brown. *Legs*: As for genus. Tibial spurs small, short, almost entirely scaled, except distal tip. *Forewing dorsum*: Forewing length: 25–27 mm, avg.: 26.1 mm, n = 6. Elongated, acutely triangular, apical half of outer margin highly concave, apex very falcate. Ground color gray or warm brown with darker gray, brown, or reddish brown suffusion, especially near interior edge of postmedial line and medial area; brownish red to blood red coloration near apex along apical interior of postmedial line, overall lightly speckled by dark petiolate scales. Discal spot faintly marked by light gray oblong shape, thin gray mark connecting discal spot to costa. Apex marked by black scales above extended apical dash. Postmedial line smooth, mostly straight, line black, strongly contrasting. Submarginal area light gray with small grayish-white suffusion mesally, postmedial lunule as white band originating from apical dash, white band follows postmedial line from apex to anal wing margin. Antemedial line faint, brown, curved outwards. *Forewing venter*: As in forewing dorsum but antemedial area lighter gray, more contrasting, blood-red suffusion more pronounced, antemedial line absent, small black discal mark occasionally present. *Hindwing dorsum*: Triangular, anal angle usually sharply accentuated, reddish coloration that bleeds into medial area originates near anal angle, overall similar coloration and patterning as forewings, antemedial line absent, postmedial line weakly curved toward anterior wing margin, not concave mesally. *Hindwing venter*: Following similar pattern as forewing venter, but red coloration near anal angle much darker, discal mark sometimes present. *Abdomen*: As for genus, but somewhat elongated, nearly sphingiform. Coloration a continuation of gray thoracic color. Midventral stripe absent. *Genitalia*: (Fig. 88) n = 3. Tegumen elongated, ovoid, constricted near base of gnathos. Vinculum broad, somewhat quadrate ventrally. Valves triangular, saccular edge of left and right valves with triangular tooth proximal to transtilla, both valves with smaller mesal costal projection originating from central ridge of valve, projection immediately above saccular edge teeth, apex of mesal costal projection pointed toward saccular edge. Valves rounded apically. Uncus truncated apically, apex somewhat quadrate. Gnathos as two prominent flattened, moderately sclerotized, flap-like, somewhat rectangular, outward facing extensions with highly truncated apices. Apices form fingerlike projections of moderate length. Juxtal processes roughly phallus length, lightly sclerotized, curving toward apex of phallus. Juxtal processes very thin, sclerotization fading to more membranous portion, covered in fine setae. Base of phallus with paired, backwards facing, very short, rounded, diverging lobes. Phallus cylindrical, smooth dorsum lacking an extensive dorsal ridge, covered in setae. Left edge of rolled phallus smooth, without ridge-like process, base of sclerotized terminus of phallus with prominent ventral bump, angled away from distal end of phallus, distal tip of phallus separated into two distinct points. Vesica somewhat elongated, covered in setae laterally, originating from progressively weakened sclerotization of diagonal edge of phallus. **Female.** *Head*: As in male. *Thorax*: As in male. *Legs*: As in male. *Forewing dorsum*: Forewing length: about 30 mm, n = 1. Maculation as in male, wing broader, less triangular, less falcate, antemedial line fainter. *Forewing venter*: As in forewing dorsum but with blood-red suffusion, antemedial line absent, thin black discal mark present. *Hindwing dorsum*: As in male but more rounded, less triangular, postmedial line straight, not concave mesally, but still only weakly curved toward anterior wing margin. *Hindwing venter*: Following similar pattern as forewing venter except lighter, reddish suffusion near anal angle much darker, contrasting. *Abdomen*: As in male but more robust. *Genitalia*: Not examined.

####### Distribution

(Map 5). *Menevia franclemonti* is found only in the Brazilian Atlantic Forest, in the states of Rio de Janeiro, São Paulo, and Santa Catarina. This species is likely endemic to this region, where it replaces the similar, more widespread *Menevia vulgaris*.

####### Etymology.

*Menevia franclemonti* is named after the lepidopterist J. G. Franclemont, who originally recognized the uniqueness of this species. He also wrote an important fascicle on the Mimallonidae of North America north of Mexico (Franclemont 1973).

####### Remarks.

This new species is the Brazilian Atlantic Forest component of the *vulgaris* subgroup of the *plagiata* species-group in much the same way that *Menevia mielkei* and *Menevia magna* are the Atlantic Forest components of the *lucara* and *lantona* species-groups respectively. Although *Menevia franclemonti* is not remarkably distinct from *Menevia vulgaris*, it is readily differentiated by the external and genitalia diagnostic characters presented above. Additionally, this species seems to be allopatric to all other species in this subgroup. Despite the differences, *Menevia franclemonti*, together with the similar *Menevia vulgaris*, have both been misidentified as *Menevia plagiata* for the reasons explained in the remarks of *Menevia plagiata* and *Menevia vulgaris*. The allopatry of *Menevia vulgaris* and *Menevia franclemonti* certainly affords that *Menevia plagiata* *sensu stricto* could not be applied to the Amazonian *Menevia vulgaris*, but it is certainly plausible that Walker’s (1855) description of *Menevia plagiata* could have applied to *Menevia franclemonti* due to its presence at the type locality of *Menevia plagiata*. However, due to the apparent rarity of *Menevia franclemonti* in collections, compared to the sympatric *Menevia plagiata* and the complete lack of *Menevia franclemonti* from the NHMUK, it is more plausible that the original material from Rio de Janeiro that Walker had at his disposal was in fact the species that we consider *Menevia plagiata* in this present work, and not *Menevia franclemonti*. We have resolved the ambiguity surrounding the application of the name *plagiata* by designating the neotype of this taxon above. If the holotype of *Menevia plagiata* is discovered in the future, and is found to be what we consider *Menevia franclemonti*, then it would be taxonomically simple to set aside the neotype under Article 75.8 of the ICZN (1999), synonymize this new species with *Menevia plagiata*, and redescribe what we currently consider to be *Menevia plagiata* as a new species.

As in the case of *Menevia australis*, J. G. Franclemont recognized this species as distinct and labeled specimens as holotype and paratypes (all from Jaraguá do Sul, Santa Catarina, Brazil) under his manuscript name *Menevia* ‡*falco*. Rather than naming this species *M.* ‡*falco* as he originally intended, we decided to honor J. G. Franclemont by naming this species after him for his work on *Menevia* and Mimallonidae in general.

###### Menevia vulgaricula sp. n.

Taxon classificationAnimaliaLepidopteraMimallonidae

http://zoobank.org/02587433-09B5-4632-AE4A-D40EB1D8651B

Figs 62 , 69 , 70 , 89 , 102 ; Map 5

####### Type material.

**Holotype**, ♂: **BRAZIL: Amazonas**: Hyutanahan [Huitanaã], Rio Purus, Brazil, S.M. Klages/ Jan. 1922/ Carn. Mus. Acc. 6963/ St. Laurent diss.: 6-19-15:1/ HOLOTYPE male *Menevia vulgaricula* St Laurent and Dombroskie, 2016 [handwritten red label]/ (CMNH). Type locality: Brazil: Amazonas: Huitanaã.

**Paratypes**, 2 ♂, 1 ♀: **BRAZIL: Amazonas**: 1 ♂, Hyutanahan [Huitanaã], Rio Purus: II.1922, S.M. Klages, Carn. Mus. Acc. 6963, St. Laurent diss.: 4-25-15:14 (CMNH). 1 ♂, Nova Olinda, Rio Purus: V.1922, S.M. Klages, St. Laurent diss.: Carn. Mus. Acc. 6962, St. Laurent diss.: 6-19-15:2 (CMNH). 1 ♀, Rio Madeira: Juillet-Août (Fassl), Dognin Collection, USNM-Mimal: 2595, St. Laurent diss.: 4-25-15:15 (USNM). – All paratypes with the following yellow label: PARATYPE male/female *Menevia vulgaricula* St Laurent and Dombroskie, 2016.

####### Additional questionable specimens examined.

(3 ♀ total) [not included in type series] **BRAZIL: Pará**: 1 ♀, Likely Belém: A.M. Moss, Rothschild Bequest 1939–1, St. Laurent 7-21-15:2, BMNH(E) 1378755 (NHMUK). **FRENCH GUIANA**: 1 ♀, RN 2, Km 48: 14.VIII.1985, J. Haxaire, Bc-Her 2991 (Collection of Daniel Herbin, France). 1 ♀, Saül: 1.VIII.2011, Ph. Collet leg. (RAS).

####### Diagnosis.

*Menevia vulgaricula* is similar to the previous two species, but much smaller, both males and females are notably smaller than the respective sexes of *Menevia vulgaris*. The female of *Menevia vulgaricula* is easily differentiated from females of *Menevia vulgaris* by the width of the submarginal area, which is broader and decreases in width toward the apex much less gradually than the rapidly narrowing submarginal area of *Menevia vulgaris*. The genitalia of both sexes can be used to differentiate *Menevia vulgaricula* from similar species (except from the unexamined female of *Menevia franclemonti*). In males, the phallus has a very prominent setae covered dorso-anterior bulge reminiscent of a dorsal phallic ridge, the tegumen, vinculum, and acutely triangular uncus are all very narrow and elongated, and the paired processes of the gnathos converge and bend upwards. Additionally, the divergent lobes at the proximal end of the phallus are not broad, but thin and peg-like. In the female, the lamella antevaginalis is very thin, unlike the usually broad, quadrate lamella of *Menevia vulgaris*.

####### Description.

**Male.** *Head*: Gray, eyes large comprising about two-thirds of head area, eyes bordered posteriorly by brownish gray collar of scales reaching labial palpi, labial palpi very small, segments weakly defined ventrally, dorsally with darker scales contrasting with overall gray coloration. Scape and pedicel tufted. *Thorax*: As for genus. Light gray. *Legs*: As for genus. Tibial spurs moderate length, thin, somewhat hooked distally. *Forewing dorsum*: Forewing length: 19–22 mm, avg.: 21 mm, n = 3. Acutely triangular, apical half of outer margin concave, apex falcate. Ground color gray with brown or reddish brown suffusion, especially near interior edge of postmedial line and medial area, reddish coloration near apex along apical interior of postmedial line, overall lightly speckled by dark petiolate scales. Discal spot faintly marked by light gray oblong shape, thin gray mark connecting discal spot to costa. Apex marked by black scales above extended apical dash. Postmedial line mostly straight except when approaching apex, line black, strongly contrasting. Submarginal area light gray with whitish suffusion mesally, sometimes appearing as a faint zigzag, postmedial lunule as white band originating from apical dash, white band follows postmedial line from apex to anal wing margin. Antemedial line faint, brown, curved outwards. *Forewing venter*: As in forewing dorsum but antemedial area lighter gray, more contrasting, blood red suffusion more expansive, antemedial line absent, small black discal spot occasionally present. *Hindwing dorsum*: Triangular, anal angle weakly accentuated, reddish coloration near anal angle, similar coloration and patterning as forewings, antemedial line absent, postmedial line moderately bent toward anterior wing margin, weakly concave mesally. *Hindwing venter*: Following similar pattern as forewing venter, but red coloration near anal angle much darker, discal mark absent or very faint. *Abdomen*: As for genus but somewhat elongated, nearly sphingiform. Coloration a continuation of gray thoracic color. Midventral stripe absent. *Genitalia*: (Fig. 89) n = 3. Tegumen elongated, narrow, somewhat constricted near base of gnathos. Vinculum elongated, narrow. Valves relatively narrow, saccular edge of left valve with large triangular tooth proximal to transtilla, right valve with tooth slightly reduced in size, both valves with smaller mesal costal projection originating from central ridge of valve, mesal costal projection immediately above saccular edge teeth, apex of projection pointed toward saccular edge. Valves rounded apically. Uncus very narrow, acutely triangular. Gnathos as two prominent, converging, flattened, sclerotized, flap-like, somewhat triangular, upward facing extensions with somewhat truncated apices. Juxtal processes shorter than phallus, curving toward apex of phallus. Juxtal processes thin, covered in fine setae. Base of phallus with paired, backwards facing, short, peg-like, diverging lobes. Phallus cylindrical, irregularly edged dorsum with accentuated bulging ridge-like projection situated anteriorly, covered in setae. Left edge of rolled phallus uneven but forming anteriorly situated bulge, base of the sclerotized terminus of phallus with prominent ventral bump, angled away from distal end of phallus, distal tip of phallus separated into two distinct points. Vesica elongate, covered in setae laterally, originating from progressively weakened sclerotization. **Female.** *Head*: As in male but with light brown tint. *Thorax*: As in male but with brownish tint. *Legs*: As in male, tibial spurs shorter or about same length. *Forewing dorsum*: Forewing length: 27 mm, n = 1. Maculation as in male, wing broader, barely more elongate, less triangular, less falcate, postmedial line straighter, nearly parallel to wing margin until just before apex, submarginal area rectangular not triangular, antemedial line fainter. *Forewing venter*: As in forewing dorsum but lighter gray, with pinkish suffusion, antemedial line absent, thin black discal mark present. *Hindwing dorsum*: As in male but more rounded, less triangular, postmedial line concave mesally, but still moderately bent toward anterior wing margin. *Hindwing venter*: Following similar pattern as forewing venter, reddish suffusion near anal angle much darker, contrasting. *Abdomen*: As in male but more robust. Sternite of VIII with pair of thin sclerotized bands converging near anterior margin of VIII forming a “V”. *Genitalia*: (Fig. 102) n = 1. Tergite of VIII forming posteriorly directed triangle, without membranous gap mesally. Apophyses anteriores about same length as apophyses posteriores. Lamella antevaginalis very thin, indistinct, curved. Ductus bursae short. Papillae anales elongated, covered in relatively long setae.

####### Distribution

(Map 5). This new species is restricted to the Amazon region, specifically in the vicinity of Rio Madeira and Rio Purus, but may be more widespread, see remarks. This species is sympatric with *Menevia vulgaris*.

####### Etymology.

*Menevia vulgaricula* is named for its appearance as a diminutive *Menevia vulgaris*.

####### Remarks.

*Menevia vulgaricula* is an interesting species due to its remarkable external resemblance to *Menevia vulgaris*, with which *Menevia vulgaricula* is sympatric. Despite notable similarity in external characters, *Menevia vulgaricula* is much smaller and displays distinct genitalia characters, in both the male and the female.

Both *Menevia vulgaris* and *Menevia vulgaricula* were collected by S. M. Klages during the same period at both Huitanaã and Nova Olinda, Amazonas, Brazil (CMNH). Therefore, not only are these two species sympatric, but are also apparently both active at the same time of year. Size and distinct differences in genitalia likely afford some form of a prezygotic barrier. The last two new species described below are very similar to *Menevia vulgaricula*, in that each species is very small relative to the larger *Menevia vulgaris* and *Menevia franclemonti*, and both display prominent anterior phallic bulges. These species however are widely allopatric. Therefore, the modes of isolation between sympatric *Menevia vulgaris* and *Menevia vulgaricula* certainly warrants future investigation.

Additional females from Pará, Brazil and French Guiana were examined, but cannot be included in the type series because we lack the more easily identifiable males from these localities. Also, these specimens display deeper red coloration and wavier forewing postmedial lines, characters not seen in all other examined *Menevia vulgaricula*. Although the small size of these females and the genitalia of the Pará specimen suggest that they are probably *Menevia vulgaricula*, Pará and French Guiana are distant from both Rio Madeira and Rio Purus, the two relatively nearby localities known to support *Menevia vulgaricula*.

###### Menevia cordillera sp. n.

Taxon classificationAnimaliaLepidopteraMimallonidae

http://zoobank.org/DE2B8D54-5671-4603-A9ED-7CC342CB46D5

Figs 63 , 64 , 90 ; Map 5

####### Type material.

**Holotype**, ♂: **PERU**: 1584, San Gaban [San Gabán], Peru, 2500 ft, March–April 1913/ Joicey Coll. Brit. Mus. 1925–157/ St. Laurent diss.: 6-29-15:8/ BMNH(E) 1378757/ HOLOTYPE male *Menevia cordillera* St Laurent and Dombroskie, 2016 [handwritten red label]/ (NHMUK). Type locality: Peru: Puno: Carabaya: San Gabán.

**Paratype**, 1 ♂: **BOLIVIA**: Rio Songo [Río Zongo], 750 m: Coll. Fassl, Dognin Collection, USNM-Mimal: 2599, St. Laurent diss.: 4-25-15:17 (USNM). – Paratype with the following yellow label: PARATYPE male *Menevia cordillera* St Laurent and Dombroskie, 2016.

####### Diagnosis.

This new species, like *Menevia vulgaricula*, is quite small in comparison with the widespread *Menevia vulgaris* and the southeast Brazilian *Menevia franclemonti*. Due to the small size of *Menevia cordillera*, it may be confused with the allopatric *Menevia vulgaricula* but can easily be differentiated by the deeper reddish brown coloration, more sharply acute apices of the more elongated forewings, straighter hindwing margins, and by the male genitalia. The phallus of *Menevia cordillera* is somewhat reminiscent of that of *Menevia vulgaricula*, but with a more triangularly shaped anterior dorsal bulge and rounded, not peg-like, lobes at the base of the phallus. Overall, the phallus of *Menevia cordillera* is broader than that of *Menevia vulgaricula*. No other *Menevia* species are known from the Cordillera Oriental, besides the clearly distinct *Menevia torvamessoria*.

####### Description.

**Male.** *Head*: Light brown-gray, eyes large comprising about two-thirds of head area, eyes bordered posteriorly by dark brown collar of scales reaching labial palpi, labial palpi large, robust for genus, dorsally with darker scales contrasting with overall gray coloration. Scape and pedicel weakly tufted. *Thorax*: As for genus. Light tan. *Legs*: As for genus. Tibial spurs short, stout. *Forewing dorsum*: Forewing length: 22–23 mm, avg.: 22.5 mm, n = 2. Very acutely triangular, apical half of outer margin deeply concave, apex very falcate. Ground color gray with deep reddish brown suffusion throughout medial area, brighter reddish coloration near apex along apical interior of postmedial line, overall lightly speckled by dark petiolate scales. Discal spot faintly marked by light gray oblong shape, gray mark connecting discal spot to costa. Apex marked by black scales above extended apical dash. Black postmedial line mostly straight except when approaching apex where sharply curved, strongly contrasting. Submarginal area light gray with whitish suffusion mesally, sometimes appearing as faint zigzag, submarginal area with distinct white band originating from apical dash, white band follows postmedial line from apex to anal wing margin. Antemedial line very faint, brown, curved outwards. *Forewing venter*: As in forewing dorsum but antemedial area lighter gray, more contrasting, antemedial line absent, small black discal mark present. *Hindwing dorsum*: Triangular, outer margin very straight, anal angle weakly accentuated, reddish coloration near anal angle, bleeding into medial area, similar coloration and patterning as forewings, antemedial line absent, postmedial line sharply bent toward anterior wing margin, weakly concave mesally. *Hindwing venter*: Following similar pattern as forewing venter, but red coloration near anal angle much darker, discal mark absent. *Abdomen*: As for genus, but elongated, nearly sphingiform. Midventral stripe absent. *Genitalia*: (Fig. 90) n = 2. Tegumen elongated, moderately narrow, weakly constricted near base of gnathos. Vinculum elongated, narrow, ovoid, somewhat rounded ventrally. Valves relatively narrow, rounded, saccular edge of left valve with large triangular tooth proximal to transtilla, right valve with tooth slightly reduced in size, both valves with smaller mesal costal tooth originating from central ridge of valve, mesal costal projection immediately above saccular edge teeth, apex of projection pointed toward saccular edge. Valves truncated apically. Uncus very narrow, acutely triangular, quadrate or rounded apically. Gnathos as two prominent, converging, flattened, sclerotized, flap-like, somewhat triangular, upward facing extensions with truncated apices. Juxtal processes shorter than phallus, curving toward apex of phallus. Juxtal processes thin, covered in fine setae. Base of phallus with paired, backwards facing, short, rounded, diverging lobes. Phallus cylindrical, dorsum with accentuated triangular bulging projection situated anteriorly, covered in setae. Left edge of rolled phallus uneven but forming anteriorly situated, setae covered, triangular bulge, base of sclerotized terminus of phallus with prominent ventral bump, angled away from distal end of the phallus, distal tip of phallus separated into two, elongated, distinct points. Vesica elongated, bag-like, covered in setae laterally, originating from progressively weakened sclerotization. **Female.** Unknown.

####### Distribution

(Map 5). *Menevia cordillera* is apparently restricted to the Cordillera Oriental of Peru and Bolivia at moderate elevations, from 750–760 m in elevation.

####### Etymology.

This new species is named for the Andean Cordillera Oriental, to which this species is endemic.

####### Remarks.

*Menevia cordillera* is very closely related to both *Menevia vulgaris* and *Menevia vulgaricula* based on general external characters and the genitalia morphology. These three species may represent taxa of a species complex that spans throughout northern South America.

An additional specimen from the Yungas of Bolivia, in the collection of Daniel Herbin (Bc-Her2532) as seen in the BOLD database, almost certainly belongs to this new species. However, we were unable to examine this specimen and thus it cannot be included in the type series, but we report it here as it provides additional distributional data.

###### Menevia delphinus sp. n.

Taxon classificationAnimaliaLepidopteraMimallonidae

http://zoobank.org/12D5AF97-8DE4-43EA-902A-26E96BB24065

Figs 65 , 66 , 71 , 91 , 103 ; Map 5.

####### Type material.

**Holotype**, ♂: **BRAZIL: Distrito Federal**: Coleção EMBRAPA-CPAC No. 9401/ Planaltina, DF, 1000 m, 15°35'S, 47°42'W, 12.XI.1982/ V.O. Becker Col./ St. Laurent diss.: 9-3-15:1/ HOLOTYPE male *Menevia delphinus* St Laurent and Dombroskie, 2016 [handwritten red label]/ (CPAC). Type locality: Brazil: Distrito Federal: Planaltina.

**Paratypes**, 6 ♂, 2 ♀: **BRAZIL: Distrito Federal**: 3 ♂, 2 ♀, Planaltina, 15°35'S, 47°42'W, 1000 m: 11.XI.1977, 15.XI.1977, V.O. Becker col., Col. Becker No. 22269, 22304, St. Laurent diss.: 4-25-15:16, USNM-Mimal: 2341, 2343 (USNM); 10.XI.1975, 3.XI.1982, 4.XI.1982, V.O. Becker col., Coleção EMBRAPA-CPAC No. 79, 9401, St. Laurent diss.: 4-2-15:4 (CPAC). 1 ♂, Planaltina, 15°36'S, 47°44'W, 960 m: 30.X1992, No. 2135, Coleção EMBRAPA-CPAC No. 12816, St. Laurent diss.: 9-2-15:3 (CPAC). **Goiás**: 1 ♂, Leop. Bulhoes [Leopoldo de Bulhões]: XI.1935, Coll. R. Spitz, Rothschild Bequest BM 1939–1, St. Laurent diss.: 6-29-15:10, BMNH(E) 1378753 (NHMUK). **Minas Gerais**: 1 ♂, Sabara-Bello Horizonte, Rio das Velhao [Sabará-Belo Horizonte, Rio das Velhas]: A.G.N. Chalmers, B.M. 1932–11, St. Laurent diss.: 6-29-15:11, BMNH(E) 1378756 (NHMUK). – All paratypes with the following yellow label: PARATYPE male/female *Menevia delphinus* St Laurent and Dombroskie, 2016.

####### Additional specimen examined from photo.

[not included in type series] **BRAZIL: Maranhão**: 1 ♂, Feira Nova do Maranhão, Retiro, 46°26'41"W, -07°00'31"S, 480 m: C. Mielke leg., Paratype, *Menevia alurca* Herbin & Mielke, 2014 (Collection of Daniel Herbin, France).

####### Diagnosis.

*Menevia delphinus*, like the previous two species, is quite small in comparison with the widespread *Menevia vulgaris* and the southeast Brazilian *Menevia franclemonti*, but males and females can easily be differentiated from all others belonging to the *plagiata* species-group by the relatively stout forewings and by the genitalia. The phallus of *Menevia delphinus* is most similar to that of *Menevia cordillera*, but with the dorsal bulge being situated more mesally along the length of the phallus and usually much more pronounced as a singular, blunt, protuberance, not a pointed or triangular bulge as in other species. Additionally, the lobes at the base of the phallus in *Menevia delphinus* are very elongated and tubular, almost fingerlike, not rounded or peg-like as in the previous similar species. In females, the VIII tergite is very robust, forming a distinct triangle, and is not rounded and arc-like as in some similar species. *Menevia delphinus* may also be confused with *Menevia alurca* due to the similar size and sympatry; however, *Menevia delphinus* can be straightforwardly recognized by the lack of a midventral abdominal stripe and a continuous white band along the outer edge of the postmedial line, which is discontinuous midway along the postmedial line in *Menevia alurca*. Additionally, the genitalia easily differentiate *Menevia delphinus* from *Menevia alurca*. The dorsal protuberance of the phallus of *Menevia delphinus* is much smaller and blunter in comparison with the extremely elongate, curved, and sharply pointed dorsal phallic protuberance of *Menevia alurca*. The only other sympatric species besides *Menevia alurca* is the much larger *Menevia vulgaris*.

####### Description.

**Male.** *Head*: Light brown or gray, eyes large comprising about two-thirds of head area, eyes bordered posteriorly by brownish collar of scales reaching labial palpi, labial palpi very small, short, covered in dark scales. Scape and pedicel weakly tufted. *Thorax*: As for genus. Light gray. *Legs*: As for genus. Tibial spurs moderate length, thin, scaled except for distal tip. *Forewing dorsum*: Forewing length: 17–23 mm, avg.: 20.5 mm, n = 6. Triangular, not overly elongated, apical half of outer margin concave, apex falcate. Ground color gray with reddish brown suffusion, especially near interior edge of postmedial line, reddish coloration near apex along apical interior of postmedial line, overall moderately speckled by dark petiolate scales. Discal spot very faintly marked by light gray oblong shape. Apex marked by black scales above apical dash. Black postmedial line mostly straight except when very near apex, strongly contrasting. Submarginal area light gray with whitish suffusion mesally, postmedial lunule as white band originating from apical dash, white band follows postmedial line from apex to posterior wing margin. Antemedial line brown, almost nonexistent. *Forewing venter*: As in forewing dorsum but antemedial area lighter gray, blood red suffusion present, especially along interior edge of postmedial line and near apex, antemedial line absent, small black discal mark occasionally present. *Hindwing dorsum*: Triangular, anal angle weakly accentuated with reddish coloration, similar coloration and patterning as forewings, antemedial line absent, postmedial line weakly bent toward anterior wing margin, weakly concave or straight mesally. *Hindwing venter*: Following similar pattern as forewing venter, but red coloration near anal angle much darker, discal mark absent or very faint. *Abdomen*: As for genus but somewhat elongated, nearly sphingiform. Coloration a continuation of gray thoracic color. Midventral stripe absent. *Genitalia*: (Fig. 91) n = 5. Tegumen ovoid, constricted near base of gnathos. Vinculum rectangular, somewhat quadrate ventrally. Valves somewhat triangular, narrow, saccular edge of left and right valves with triangular tooth proximal to transtilla, both valves with smaller mesal costal projection originating from central ridge of valve, mesal costal projection immediately above saccular edge teeth, apex of projection toward saccular edge. Valves rounded or nearly pointed apically. Uncus truncated apically, apex rounded. Gnathos as two prominent flattened, moderately sclerotized, flap-like, somewhat triangular, upward facing extensions with highly truncated apices. Apices form elongated fingerlike projections. Juxtal processes roughly phallus length, weakly sclerotized, curving toward apex of phallus. Juxtal processes thin, covered in fine setae. Base of phallus with paired, backwards facing, elongated, fingerlike, diverging lobes. Phallus broad, irregularly edged dorsum usually with accentuated, rounded mesal protuberance but sometimes much reduced and flattened, always covered in setae. Left edge of rolled phallus forming mesally situated protuberance, base of sclerotized terminus of phallus with weak ventral bump, angled ventrally or away from end of phallus, distal tip of phallus separated into two distinct points. Vesica somewhat elongated, bag-like, covered in setae laterally, originating from progressively weakened sclerotization of diagonal edge of phallus. **Female.** *Head*: As in male. *Thorax*: As in male. *Legs*: As in male. *Forewing dorsum*: Forewing length: 26 mm, n = 1. Maculation as in male, wing slightly broader, barely more elongate, less triangular, less falcate. *Forewing venter*: As in forewing dorsum but lighter gray, antemedial line absent, thin black discal mark present. *Hindwing dorsum*: As in male but more rounded, less triangular. *Hindwing venter*: Following similar pattern as forewing venter. *Abdomen*: As in male but more robust. Sternite of VIII as pair of thin sclerotized bands converging near anterior margin of VIII forming well-defined rectangular sternite. *Genitalia*: (Fig. 103) n = 1. Tergite of VIII robust, forming posteriorly directed triangle, membranous gap mesally. Apophyses anteriores about same length as apophyses posteriores, apophyses posteriores slightly thicker. Lamella antevaginalis thin, curved, slightly indented mesally near ostium bursae. Ductus bursae short. Papillae anales elongated, covered in relatively long setae.

####### Distribution

. This new species is a resident of the Brazilian Cerrado in the states of Goiás and Minas Gerais, as well as in Distrito Federal. An additional record from Maranhão will be discussed below in the remarks.

####### Etymology.

This new species is named for the phallus, which, due to the dorsal protuberance, bears the likeness of a dolphin (=delphinus Latin) when viewed laterally.

####### Natural history.

Dr. A. Camargo (CPAC) kindly provided additional information pertaining to one of the paratypes (specimen number 12816), and is thus the only available natural history information for this new species. The pupa of this specimen was collected on *Miconia albicans* (Melastomataceae) on 8.VI.1992 and the subsequent adult eclosed on 30.X.1992. This is the only record of *Menevia* found on Melastomataceae, but cannot be definitively considered a host record without determining if this species was feeding on the plant prior to pupation.

####### Remarks.

*Menevia delphinus* represents another species similar to the large, widespread *Menevia vulgaris* and its Brazilian Atlantic Forest counterpart, *Menevia franclemonti*. *Menevia delphinus*, like three other similar species described as new in the present work, is much smaller and with much more complicated phallic structures than *Menevia vulgaris* and *Menevia franclemonti*.

Due to the lack of data regarding the distribution of this species, other than it clearly being found in the Brazilian Cerrado, we consider it is necessary to report another state record for this species, despite our inability to gain access to the specimen in question. The recently described, and very distinct, *Menevia alurca*, was described from eight males (Herbin and Mielke 2014). The two authors of *Menevia alurca* were kind enough to supply us with specimens or photos of specimens such that the holotype and all seven paratypes could be examined. Upon close examination, it was discovered that a single undissected paratype, from the type locality of *Menevia alurca*, fits our concept of *Menevia delphinus* based on the continuous white band along the external edge of the postmedial line, and the lack of a ventral abdominal line. *Menevia alurca* has a discontinuous white band and a very prominent dark, ventral, abdominal line. We therefore consider *Menevia delphinus* to be present in Maranhão, which is highly likely given the Cerrado habitat there (Herbin and Mielke 2014).

The single reared specimen of *Menevia delphinus* was much smaller than other *Menevia delphinus* specimens and its genitalia differed in the shape of the dorsal protuberance of the phallus, which was flattened rather than distinctly raised. However, other genital characteristics, such as the highly elongated lobes at the base of the phallus, were consistent with *Menevia delphinus*. Given the consistency in other characters and the close proximity of this specimen’s locale to the type locality of *Menevia delphinus*, we include this specimen in the type series and attribute the different phallic structure to the overall small size.

###### Mimallo saturata

Taxon classificationAnimaliaLepidopteraMimallonidae

Walker, 1855, nomen dubium

####### Type material.

**Holotype**, ♀, presumed lost/destroyed. Type locality: Brazil: Rio de Janeiro (see remarks of *Menevia plagiata*).

The unknown taxon *Mimallo saturata* was briefly discussed above in the remarks relating to *Menevia plagiata* because some specimens of *Menevia* had previously been attributed to *Mimallo saturata* (USNM; BOLD database). Given that the original description of *Mimallo saturata* includes characters that are not known in any *Menevia*, or even in any Mimallonidae, such as a red abdomen with yellow hairs and an orange stripe along each side, we treat *Mimallo saturata* as a *nomen dubium* until specimens from near the type locality matching this description can be located.

## Additional discussion

*Menevia* is a wide-ranging genus displaying distinct patterns of speciation. For example, both the *lantona* and *lucara* species-groups are widely sympatric, with exceptions only in Central America. In both species-groups we discovered taxa distinct from the nominotypical species on the peripheries of those species’ distributions, in southeastern Brazil, Central America, and the Andean Cordillera Oriental. The *plagiata* species-group also showed similar patterns of speciation in the Andean Cordillera Oriental and southeastern Brazil. Additionally, the *plagiata* and *ostia* species-groups both have unique Brazilian Cerrado species. The broad overlap in the distributions of the four species-groups suggests that they may have originated from the same geographic region, with various degrees of parallel speciation.

The distinct dorsal phallic ridge or projection of the male genitalia is an important character used to differentiate species in this genus, and interestingly, all species from southeastern Brazil, except *Menevia franclemonti*, display this character, whereas all Amazonian and Central American species, except *Menevia ostia*, do not. Additionally, the midventral abdominal stripe is present only in central and southeastern Brazilian species, namely in *Menevia plagiata*, *Menevia alurca*, *Menevia australis*, and *Menevia magna*. This trait is not present in Amazonian or Central American species. Most species displaying the midventral abdominal stripe belong to a closely-knit section of the *Menevia plagiata* subgroup of the *plagiata* species-group, therefore its presence in *Menevia magna* of the *lantona* species-group implies that this trait may be plesiomorphic along with dorsal ridges/projections of the phallus which this species also displays. Furthermore, St Laurent and Dombroskie (2015) showed that *Eadmuna pulverula* (Schaus, 1896), also of southeastern Brazil, possesses a midventral abdominal stripe as well, which may be a character useful to aid in determining the phylogenetic relationships among these taxa.

A clearer understanding of the evolutionary origins of *Menevia*, and Mimallonoidea as whole, would be particularly interesting because recent higher-level phylogenetic studies of Lepidoptera continue to demonstrate not only the uniqueness of Mimallonidae as the only family in Mimallonoidea, but also its key phylogenetic role as a potential sister lineage to the Macrolepidoptera (Timmermans et al. 2014, Kawahara and Breinholt 2014).

## Supplementary Material

XML Treatment for Menevia

XML Treatment for Menevia lantona

XML Treatment for Menevia rosea

XML Treatment for Menevia torvamessoria

XML Treatment for Menevia magna

XML Treatment for Menevia lucara

XML Treatment for Menevia menapia

XML Treatment for Menevia mielkei

XML Treatment for Menevia ostia

XML Treatment for Menevia parostia

XML Treatment for Menevia pallida

XML Treatment for Menevia plagiata

XML Treatment for Menevia alurca

XML Treatment for Menevia australis

XML Treatment for Menevia vulgaris

XML Treatment for Menevia franclemonti

XML Treatment for Menevia vulgaricula

XML Treatment for Menevia cordillera

XML Treatment for Menevia delphinus

XML Treatment for Mimallo saturata

## References

[B1] Becker VO (1996) Mimallonidae. In: Heppner JB et al. (Eds) Atlas of Neotropical Lepidoptera, Checklist. Part 4B. Drepanoidea, Bombycoidea, Sphingoidea. Association for Tropical Lepidoptera & Scientific Publishers, Gainesville, Florida, 17–19.

[B2] Becker VO (2001) The identity of some unrecognized Neotropical Bombycoidea (Lepidoptera) described by Francis Walker. Revista Brasileira de Zoologia 18(1): 153–157. doi: 10.1590/S0101-81752001000100018

[B3] Barcode of Life [or BOLD or BOLDSYSTEMS] (2013) Advancing species identification and discovery by providing an integrated environment for the assembly and application of DNA barcodes. Barcode of life data systems. http://www.barcodinglife.org or www.boldsystems.org [10/25/2015]

[B4] Druce H (1891–1900) InsectaLepidoptera-Heterocera, Vol. II: (text). In: Biologia Centrali-Americana, Porter RH Publishers, London, 622 pp.

[B5] Dyar HG (1914) Report on the Lepidoptera of the Smithsonian biological survey of the Panama Canal Zone. Proceedings of the United States National Museum 47: 139–350. doi: 10.5479/si.00963801.47-2050.139

[B6] Fletcher DS Nye IWB (1982) Bombycoidea, Castnioidea, Cossoidea, Mimallonoidea, Sesioidea, Sphingoidea, Zygaenoidea. In: Nye IWB (Ed.) The generic names of moths of the world, vol. 4 Trustees of the BMNH, London, xiv + 192 pp.

[B7] Forbes WTM (1942) The Lepidoptera of Barro Colorado Island, Panama (No. 2). Bulletin of the Museum of Comparative Zoology, Harvard, 90(2): 265–406.

[B8] Franclemont JG (1973) Mimallonoidea (Mimallonidae) and Bombycoidea (Apatelodidae, Bombycidae, Lasiocampidae). In: Dominick RB Ferguson DC Franclemont JG Hodges RW Munroe EG (Eds) The moths of North America north of Mexico fasc. 20.1. E.W. Classey Ltd. and Richard B. Dominick Publ., London, 86 pp.

[B9] Herbin D (2012) Descriptions of a new genus and ten new species of Mimallonidae (Lepidoptera: Mimallonoidea). The European Entomologist 4(1): 1–31.

[B10] Herbin D (2015) Description de nouvelles espèces d’Hétérocères de Guyane française avec notes taxinomiques (Lepidoptera Mimallonidae et Apatelodidae). Antenor 2(1): 81–105.

[B11] Herbin D Mielke CG (2014) Preliminary list of Mimallonidae from Feira Nova do Maranhão, Maranhão, northern Brazil with descriptions of some new species (Lepidoptera Heterocera Mimallonoidea). Antenor 1(2): 130–152.

[B12] IBGE (2004) Mapa de Biomas e de Vegetação, Instituto Brasileiro de Geografia e Estatística http://www.ibge.gov.br/home/presidencia/noticias/21052004biomashtml.shtm [10/25/2014]

[B13] ICZN [International Commission on Zoological Nomenclature ed.] (1999) International Code of Zoological Nomenclature, fourth edition, adopted by the International Union of Biological Sciences International Trust for Zoological Nomenclature, NHMUK, London, xxix + 306 pp.

[B14] Janzen DH Hallwachs W (2009) Dynamic database for an inventory of the macrocaterpillar fauna, and its food plants and parasitoids, of Area de Conservacion Guanacaste (ACG), northwestern Costa Rica (nn-SRNP-nnnnn voucher codes) http://janzen.sas.upenn.edu

[B15] Janzen DH Hallwachs W Blandin P Burns JM Cadiou J Chacon I Dapkey T Deans AR Epstein ME Espinoza B Franclemont JG Haber WA Hajibabaei M Hall JPW Hebert PDN Gauld ID Harvey DJ Hausmann A Kitching I Lafontaine D Landry J Lemaire C Miller JY Miller JS Miller L Miller SE Montero J Munroe E Rab Green S Ratnasingham S Rawlins JE Robbins RK Rodriguez JJ Rougerie R Sharkey MJ Smith MA Solis MA Sullivan JB Thiaucourt P Wahl DB Weller SJ Whitfield JB Willmott KR Wood DM Woodley NE Wilson JJ (2009) Integration of DNA barcoding into an ongoing inventory of complex tropical biodiversity. Molecular Ecology Resources 9 (Supplement 1): 1–26. doi: 10.1111/j.1755-0998.2009.02628.x 21564960 10.1111/j.1755-0998.2009.02628.x

[B16] Janzen DH Hallwachs W (2011) Joining inventory by parataxonomists with DNA barcoding of a large complex tropical conserved wildland in northwestern Costa Rica. PLoS ONE 6(8): . doi: 10.1371/journal.pone.0018123 10.1371/journal.pone.0018123 PMC3156711 21857894

[B17] Kawahara AY Breinholt JW (2014) Phylogenomics provides strong evidence for relationships of butterflies and moths. Proceedings of the Royal Society B, 281: . doi: 10.1098/rspb.2014.0970 10.1098/rspb.2014.0970 PMC4083801 24966318

[B18] Lafontaine JD (1987) Noctuoidea: Noctuidae (part). In: Dominick RB Ferguson DC Franclemont JG Hodges RW Munroe EG (Eds) The moths of North America north of Mexico fasc. 27.2. Wedge Entomological Research Foundation, Washington, 237 pp.

[B19] Lemaire C Minet J (1999) The Bombycoidea and their relatives. In: Kristensen NP (Ed.) Lepidoptera: Moths and Butterflies. 1. Evolution, systematics, and biogeography. Part 35. Handbook of Zoology/Handbuch der Zoologie. IV, Walter de Gruyter, Berlin, New York, 321–353.

[B20] Lima AMC (1950) Insetos do Brasil. Lepidópteros, Tomo VI (Parte II). Escola Nacional de Agronomia, Rio de Janeiro, 422 pp.

[B21] Lucas R (1915) Jahresbericht für 1914, Insecta Lepidoptera für 1914, Systematik. Archiv für Naturgeschichte 81B, Heft 7: 249 pp.

[B22] Mecke R Galileo MHM Engles W (2000) Insetos e ácaros associados à *Araucaria angustifolia* (Araucariaceae, Coniferae) no sul do Brasil. Iheringia, Série Zoologia, Porto Alegre, Rio Grande do Sul 88: 165–172.

[B23] Mielke CG Rougerie R Decaëns T (2012) A new *Scolesa* Michener, 1949 from southeastern Brazil (Lepidoptera: Saturniidae, Ceratocampinae). Nachrichten des Entomologischen Vereins Apollo, Frankfurt am Main, N.F., 33(2): 81–86.

[B24] Monte O (1934) Borboletas que vivem em plantas cultivadas. Boletim de Agricultura, Zootecnica e Veterinária, Série Agrícola, No. 21 Belo Horizonte, Minas Gerais, 219 pp.

[B25] Pastrana JA (2004) Los Lepidópteros Argentinos. Sus plantas hospedadoras y otros sustratos alimenticios. Braun K Logarzo G Cordo HA Di Iorio OR (Coordinadores) Sociedad Entomológica Argentina ediciones Buenos Aires, 334 pp.

[B26] Pearson HR (1951) Contribuição ao conhecimento do gênero “*Mimallo*” Hübner,1920 (Lepidoptera, Mimallonidae). Revista Brasileira de Biologia, Rio de Janeiro 11: 315–332.

[B27] Pearson HR (1984) Sôbre o gênero *Tolypida* Schaus, 1928 (Lepidoptera, Mimallonidae) com descrição de nova espécie. Revista Brasileira de Entomologia, Rio de Janeiro 28: 459–464.

[B28] Raymundo B (1919) Noticia sôbre alguns Lepidopteros serígenos do Brasil. Typographia Revista dos Tribunaes, Rio de Janeiro, 55.

[B29] Schaus W (1905) Descriptions of new South American moths. Proceedings of the United States National Museum 29: 179–345. doi: 10.5479/si.00963801.1420.179

[B30] Schaus W (1928) Familie Mimallonidae. In: Seitz A (Ed.) Die Gross-Schmetterlinge der Erde. 6. Die amerikanischen Spinner und Schwärmer. A. Kernen, Stuttgart, 635–672.

[B31] Shorthouse DP (2010) SimpleMappr, an online tool to produce publication-quality point maps. http://www.simplemappr.net [date of access: 9/20/201]

[B32] Silva AGD Gonçalves CR Galvão DM Gonçalves AJL Gomes J Silva M do N Simoni L (1968) Quarto catálogo dos insetos que vivem nas plantas do Brasil: seus parasitas e predadores. Ministério da Agricultura, Rio de Janeiro 1(2): 622 pp.

[B33] Silva JO Carvalho-Filho FDS Esposito MC Reis GA (2012) First record of *Chrysomya rufifacies* (Macquart) (Diptera, Calliphoridae) from Brazil. Revista Brasileira de Entomologia 56(1): 115–118. doi: 10.1590/S0085-56262012000100019

[B34] St Laurent RA Dombroskie JJ (2015) Revision of the genus *Eadmuna* Schaus, 1928 (Lepidoptera, Mimallonidae) with a description of a new species from French Guiana. ZooKeys 494: 51–68. doi: 10.3897/zookeys.494.9208 25901114 10.3897/zookeys.494.9208 PMC4400377

[B35] Timmermans MJ Lees DC Simonsen TJ (2014) Toward a mitogenomic phylogeny of Lepidoptera. Molecular phylogenetics and evolution 79: 169–178. doi: 10.1016/j.ympev.2014.05.031 24910155 10.1016/j.ympev.2014.05.031

[B36] Walker F (1855) List of the Specimens of Lepidopterous Insects in the Collection of the British Museum (6): 1341. Printed by order of the Trustees, London, 1854–66. doi: 10.5962/bhl.title.58221

